# How serially homologous neuroblasts produce different temporal cohorts along the *Drosophila* larval body axis

**DOI:** 10.1242/dev.205389

**Published:** 2026-10-12

**Authors:** Deeptha Vasudevan, Yi-wen Wang, Hannah Carr, Elise Paniel, Sean Corcoran, Chris C. Wreden, Elaine Kushkowski, Conor Lee-Smith, Ellie S. Heckscher

**Affiliations:** ^1^ Department of Molecular Genetics and Cell Biology, University of Chicago, Chicago, IL 60637, USA; ^2^ Paris Diderot University, 75013 Paris, France; ^3^ Committee on Development, Regeneration and Stem Cell Biology, University of Chicago, Chicago, IL 60637, USA; ^4^ Institute for Neuroscience, University of Chicago, Chicago, IL 60637, USA

**Keywords:** Temporal cohorts, Neural stem cell proliferation, Segmental variations, Nerve cord, Neural differentiation, Somatosensory circuits

## Abstract

Neural stem cells are highly flexible, generating different neuronal lineages depending on context. In the *Drosophila* CNS, 30 types of neural stem cells, or neuroblasts, are repeated in each segment as serial homologs, but produce distinct circuits specialized for different body regions. The full *in vivo* heterogeneity in stem cell development is still poorly understood. This study aimed to uncover the cellular mechanisms behind the development of regionally specialized circuits from serially homologous neuroblasts, focusing on NB3-3 lineages as a model. Using lineage tracing, Notch manipulations, marker analysis, cell death blockade and circuit tracing, we mapped neurogenesis and circuit assembly for each NB3-3 lineage across all segments. Our results show that serially homologous neuroblasts in certain regions modify proliferation patterns, adding or omitting temporal cohorts, leading to extra neurons for specialized circuits. Four types of temporal cohorts are produced, but one type, which we term a ‘fundamental’ temporal cohort, appears in every region. Neurons from this cohort are further refined post-mitotically, enabling regional circuit specialization. Our study reveals a framework for the heterogeneous development of serially homologous stem cells.

## Teaser

**Summary:** Neural stem cells are highly flexible, generating different neuronal lineages depending on context. This study identifies constraints and heterogeneity in development and provides a new model for how serially homologous stem cells produce circuits specialized for different body regions.

## INTRODUCTION

Animals have regionalized bodies that require specialized circuits in the CNS, yet neurogenesis arises from axially repeating stem cell pools. Pools of progenitors are found from the rostral hindbrain through the caudal spinal cord (Goulding and Lamar, 2000; Francius et al., 2013). Limb versus trunk spinal cords have different numbers of motor neurons and interneurons (Dasen et al., 2005; Sweeney et al., 2018). In *Drosophila*, neuroblasts occur as serially homologous sets in each CNS segment (Birkholz et al., 2013; Technau et al., 2014; Kunz et al., 2012). In the thorax versus the abdomen, one type of neuroblast can generate lineages of varying size and cell types (Birkholz et al., 2013; Technau et al., 2006; Schmid et al., 1999; Homem and Knoblich, 2012; Kraft and Urbach, 2014; Lacin et al., 2009; Baumgardt et al., 2014; Bossing et al., 1996; Schmidt et al., 1997; Skeath and Thor, 2003). However, the cellular mechanisms by which regionally specialized circuits are produced from axially repeating stem cells remain poorly defined.

The *Drosophila* CNS is organized into four regions: the subesophageal zone (SEZ), thorax (Th), abdomen (A) and terminus (Te) (Fig. 1A). Circuits in these regions process somatosensory stimuli and control the movements of the mouthparts, head, midbody and tail, respectively (Diao et al., 2024). Each region comprises multiple segments, and each ‘hemisegment’, or half-segment, of the bilaterally symmetric CNS contains 30 distinct neuroblast types (Fig. 1A).

Herein, we study NB3-3 neuroblast lineages, a powerful model for studying stem cell-based circuit assembly. NB3-3 neuroblasts are found in 14 segments (Fig. 1A), and can proliferate differently (Tsuji et al., 2008; Baumgardt et al., 2014; Gunnar et al., 2016; Truman et al., 2010). In the thorax, NB3-3 neuroblasts divide using type 1 divisions to self-renew and produce ganglion mother cells (GMC), which divide to make a Notch ON/A and Notch OFF/B neuron (Fig. 1B); these neuroblasts enter quiescence midway through embryogenesis (Fig. 1B). In the abdomen, NB3-3 uses one type 1 division followed by type 0 divisions, where it divides to self-renew and to produce a single Notch OFF/B neuron (Fig. 1B); these neuroblasts divide throughout embryonic neurogenesis. The proliferation patterns of NB3-3 neuroblasts in other regions are unknown.

Temporal cohorts, a framework used in this study, are lineage fragments, or sets of five to seven neurons born within a tight time window*.* Temporal cohorts were first identified in the NB3-3 lineage (Wreden et al., 2017) and comprise early-born and late-born Even-skipped (Eve)-Lateral (EL) neurons. NB3-3 is the only lineage that produces ELs (Bossing et al., 1996; Gunnar et al., 2016). All EL neurons require somatosensory stimuli to fire, making them sensory processing neurons (Heckscher et al., 2015). Generally, ELs are commissural and excitatory, like their counterparts in the vertebrate spinal cord, the commissural excitatory EVX-expressing (Eve homolog) V0v interneurons (Heckscher et al., 2015; Jay and McLean, 2019). The initial assumption was that all ELs were similar, but this was challenged because only late-born ELs express an 11F02 enhancer fragment (Heckscher et al., 2014). Independently activating the early-born and late-born ELs triggered escape and postural control behavioral responses, respectively (Wreden et al., 2017). Early-born ELs respond to vibrational stimuli, whereas late-born ELs respond to proprioceptive stimuli (Wreden et al., 2017). Despite some morphological differences, all five early-born ELs in segment A1 receive direct synaptic input from chordotonal (vibration-sensitive) sensory neurons. All six late-born ELs in A1, despite some morphological differences, received direct synaptic input from proprioceptive sensory neurons (Wang et al., 2022). Thus, temporal cohorts are lineage fragments of similar, but non-identical, neurons that nonetheless share function and connectivity. Extending the idea of temporal cohorts to lineages beyond NB3-3, other groups of morphologically similar but not identical neurons united by similar birth timing, stem cell parent and Notch status exhibit significantly similar connectivity patterns (Mark et al., 2021). Temporal cohorts and temporal cohort-like units are found in the *Drosophila* nerve cord and mushroom body (Meng et al., 2019, 2020; Wang et al., 2022; Mark et al., 2021; Baek and Mann, 2009; Kunz et al., 2012), as well as the mouse spinal cord and neocortex (Stam et al., 2012; Gao et al., 2014). Generally, the importance of temporal cohorts lies in *Drosophila* embryonic neuroblasts populating larval circuits by assembling temporal cohorts into circuit motifs (Wang et al., 2022). A significant gap in temporal cohort biology is how their numbers and types vary across the body axis.

NB3-3 lineages are currently the only example of axial diversity in the production of temporal cohorts. In segment A1, NB3-3 produces an early-born Notch OFF/B cohort with five ELs and a late-born Notch OFF/B cohort with six ELs (Fig. 1C). In the thorax, NB3-3 neuroblasts produce an early-born Notch OFF/B cohort with ∼5 neurons and a presumed early-born Notch ON/A cohort with 5 neurons (Fig. 1C). NB3-3 lineages are less well-characterized elsewhere. Nonetheless, these data show that, between the thorax and segment A1, NB3-3 neuroblasts produce two different sets of neurons (i.e. temporal cohorts), the types of which differ in part. This finding raises the possibility that, along the body axis, development produces neurons in defined sets.

We used a combination of lineage tracing, Notch manipulations, marker analysis, cell death blockade and anterograde circuit tracing to identify patterns of neurogenesis and to track circuit assembly for each NB3-3 neuroblast lineage along the body axis. Our data advance the field by showing that by altering proliferation patterns, neuroblasts add or omit sets of five to seven neurons in various segments. One set, the early-born Notch ON/B neurons, is produced in all regions. Exclusive to this set of five to seven early-born Notch ON/B neurons, a variety of post-mitotic mechanisms refine both their number and connectivity.

## RESULTS

### Even-skipped Lateral numbers differ in different segments

To understand the variation in neurogenesis in NB3-3 lineages along the body axis, we counted the number of EL neurons in each hemisegment in larvae (Fig. 1D,E). In most hemisegments, like A1, ELs form a lateral cluster labeled with an anti-Even-skipped (Eve) antibody (Fig. 1D). In the larval thorax there are five or six ELs (Fig. 1E), as in embryos (Heckscher et al., 2014). In A1 there are 11 ELs, and in A2-A7 there are 12 ELs (Fig. 1E; Fig. S1). In the terminus, there are one to three ELs (Fig. 1E). SEZ tissue architecture is complex, with four EL clusters per two hemisegments (Fig. S2A). We additionally marked ELs with *EL-GAL4*-driven membrane GFP, and the SEZ border with anti-Scr (Fig. S2B,C). In the SEZ, there are 10-12 ELs (Fig. 1E). In summary, by the larval stage, the number of ELs varies across segments, ranging from one to 12, indicating profound differences in NB3-3 lineage development among body regions.

**Fig. 1.**
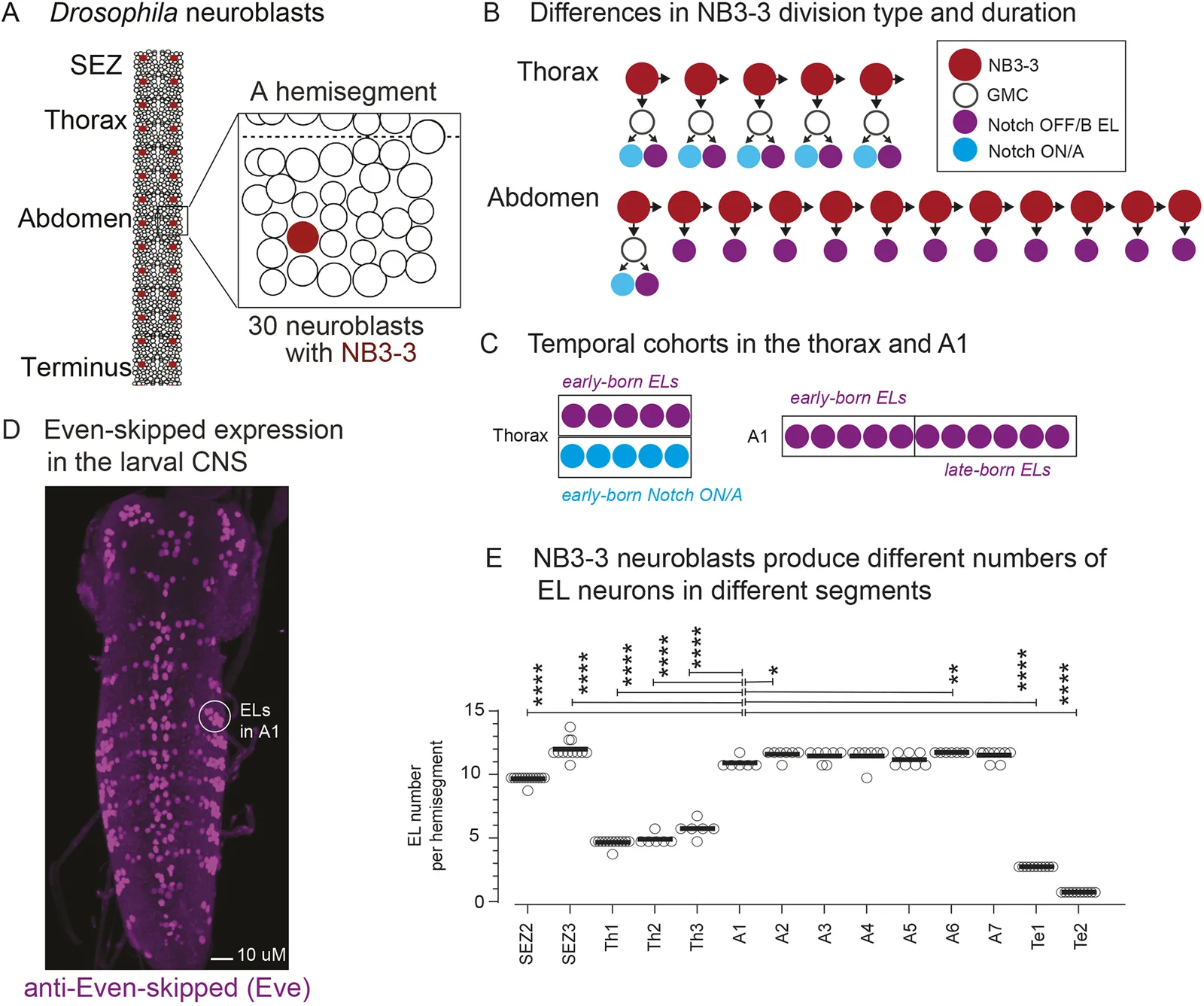
**Depending on the segment, NB3-3 produces a 10-fold difference in the number of EL neurons.** (A-C) Illustrations of *Drosophila* neuroblasts and neurons, in which one circle represents one cell. The *Drosophila* CNS is organized into four regions: the subesophageal zone (SEZ), thorax, abdomen and terminus (A). Each region comprises multiple segments, and each hemisegment contains 30 distinct neuroblast types. Schematic of lineage progression of NB3-3 in the thorax and abdomen. (B). Schematic of the NB3-3 lineage in the thorax and first abdominal segment organized as temporal cohorts. (C). Each arrow represents one cell division. Rectangles denote the groups of neurons comprising a temporal cohort. (D) Image of a wild-type larval CNS is shown with the anterior top. EL neurons in segment A1 are highlighted. (E) Quantification of EL number. Each circle represents the count in a hemisegment. Black lines denote mean. Ordinary one-way ANOVA (****P<0.0001; **P<0.01; *P<0.05). Full genotypes for all figures are found in Table S3.

### NB3-3 neuroblasts produce different sets of five to seven neurons in each region

Stem cell proliferation plays a significant role in regulating neuron number (Homem et al., 2015). Generally, stem cells vary their proliferation patterns by altering how many times they divide (herein, division duration), and/or changing whether they produce a proliferative or a post-mitotic daughter (herein, division type 1 or 0, respectively). NB3-3 neuroblasts in the thorax and abdomen exhibit varying proliferation patterns, resulting in different numbers and types of temporal cohorts (Fig. 1C). However, it is unknown what range of proliferation patterns any complete population of serially homologous neuroblasts uses. So, we needed to investigate NB3-3 neuroblast proliferation patterns across regions.

We used an inducible lineage-tracing approach, Twin-Spot Mosaic Analysis with a Repressible Cell Marker (ts-MARCM), to understand the dynamic production of EL during neurogenesis (Yu et al., 2009). In ts-MARCM, initially, every cell is heterozygous: one chromosome with an FRT site, UAS-GFP and UAS-RFP-RNAi, and the other with an FRT site, UAS-RFP and a UAS-GFP-RNAi. In heterozygous cells, neither GFP nor RFP can be produced (Fig. 2A′). Heat shocks are applied to the animal, which deliver FLP recombinase to random cell sets (Fig. 2A″). When a dividing cell recombines, daughter cells can become homozygous (Fig. 2A‴), with one daughter capable of producing GFP and the other RFP. All the descendant cells of each daughter cell will also be homozygous, generating the potential for ‘twin-spot clones’ (Fig. 2C). Herein, ‘clone’ refers to a group of cells labeled by one recombination event. Because GFP and RFP are under UAS control, they must be expressed using a GAL4 line (Fig. 2A⁗).

**Fig. 2.**
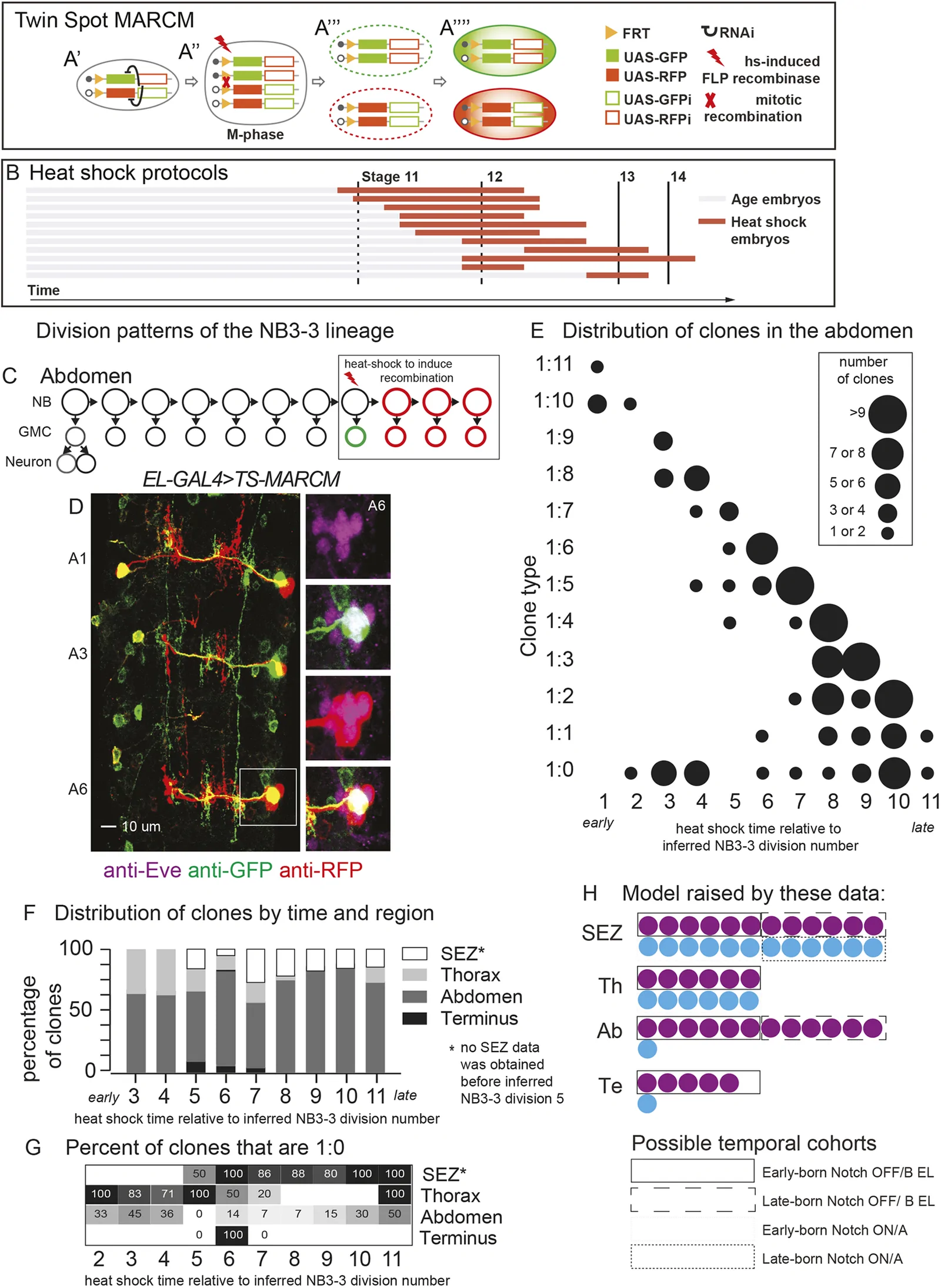
**NB3-3 neuroblasts exhibit distinct proliferation patterns across regions to produce distinct sets of neurons.** (A′-A⁗) Schematic of Twin Spot MARCM, showing how heat shock-induced recombination leads to different color fluorescent cells. (B) Illustration of the heat shock protocols and datasets used in this study. (C,D) Illustration and image of ts-MARCM labeling. (C) NB3-3 lineage progression with colors corresponding to the boxed clone in D. Each circle is one cell, and each arrow is one division. (D) A larval CNS with several ts-MARCM clones is shown with the anterior up. Segment A6 (boxed) is enlarged in the right panels. (E-G) Quantifications of the dataset in which the *x*-axis represents heat shock time relative to the inferred NB3-3 division round. (E) Quantification of the clones in abdominal segments, with rows representing clone type. #:# (e.g.1:0, 1:1, etc.) corresponds to the number of cells labeled in each color. For example, the box in C is a 1:3 clone. (F) Quantification of how clones are distributed across regions. (G) Quantification of the proportion of total clones that are small (i.e. 1:0) in each region. (H) Illustration of temporal cohorts likely produced by NB3-3 across regions. Each circle is a neuron. Ab, abdomen; GMC, ganglion mother cell; NB, neuroblast; SEZ, subesophageal zone; Te, terminus; Th, thorax.

In this study, we used ts-MARCM because of its ability to capture the dynamic process of neurogenesis. However, these data can be messy. First, the timing of heat-shock-induced recombination sets the upper limit on the number of cells within a clone. But the lower limit can depend on other factors. There may be cell-type specificity because the same RNAi constructs can have different efficacies in GMCs versus neuroblasts (Yu et al., 2009), and certain cell types could respond differently to externally applied heat shock. Moreover, the GAL4 driver line used in each experiment can set the lower limit. Herein, we use *EL-GAL4*, a small enhancer fragment (∼700 bp) that does not capture the full regulatory context of the *eve* locus. *EL-GAL4* drives highly specific, but somewhat variable, expression, sometimes labeling only a subset of cells (e.g. Manning et al., 2012). Thus, in some cases, clones detected with *EL-GAL4* may be smaller than expected.

We used ts-MARCM data produced by 11 different protocols (Fig. 2B). In each protocol, embryos were collected for 2-3 h and aged for 5-10 h at 25°C (Fig. 2B, gray bars). Then they were heat-shocked for 20-30 min (Fig. 2B, red bars). Embryos were grown to larval stages, then dissected and imaged. Part of our dataset (Table S1, experiments a-h) was produced for a previous study (Wang et al., 2022), which focused exclusively on segment A1. We re-analyzed them for this study and included all segments present in those images, which included from Th1 to Te2. However, this dataset lacked information about the SEZ. So, we produced a second dataset (Table S1, experiments i-k), In this new dataset, we imaged all segments from the SEZ to the terminus (Table S1). However, for this second dataset, we biased heat shocks towards later in embryogenesis because our main question (see below) was whether the SEZ was abdomen-like (producing late-born ELs) or thoracic-like (not producing late-born ELs). Therefore, in the data presented below, we lack ts-MARCM experiments that included the SEZ for early neuroblast divisions. Combined, our data comprise 80 CNS and 234 clones. Every clone is listed in Table S1. Despite this large dataset, there are notable gaps, which we discuss in more detail in the Materials and Methods.

We used an internal reference to estimate when heat shocks were provided to the embryos to induce recombinase expression and initiate the lineage trace. This internal reference system operates on the developmental principle that, in short-germ-band insects like *Drosophila*, segments develop synchronously. So, it is reasonable to assume that neuroblasts are dividing at approximately the same time along the anterior-posterior axis, and there are no differences between left and right hemisegments. We found that most CNSs had multiple clones (Fig. 2D). For each CNS, we assessed the location and size of each clone. Because we know NB3-3 in the abdomen divides for the duration of embryogenesis (Gunnar et al., 2016), the number of EL labeled in the abdomen is directly related to the number of NB3-3 divisions yet to occur. If one or more clones were found within the abdomen, we used them to calculate the maximum abdominal clone size per CNS; this serves as our internal reference. The maximum abdominal clone size is a proxy for the earliest time at which an embryo could have been heat-shocked. We sorted each clone into bins based on its maximum abdominal clone size in its CNS. This binning generated the *x*-axis in Fig. 2E-G and is labeled as ‘heat shock relative to inferred NB3-3 division number’. Notably, because we used this method, we excluded the rare clones that were SEZ-, thorax- or terminus-only from the analysis.

To determine the duration of division of NB3-3 in each region, we used this logic: a heat shock can generate a clone only when delivered to a dividing cell (Fig. 2A,C), and never when delivered to a non-dividing cell. We counted the number of clones at each time point and region. In the abdomen, ELs were labeled at every inferred division (Fig. 2E,F), as expected based on previous work (Gunnar et al., 2016). In the thorax and terminus, ELs were labeled only at early induction times, except for a single clone in the thorax produced in the inferred 11th division (see Materials and Methods) (Fig. 2F). This was an expected result for the thorax (Tsuji et al., 2008). In the SEZ, for which we have only late-induction data, ELs were labeled at all assayed time points (Fig. 2F). This fits a general model in which NB3-3s in non-adjacent regions (abdomen and SEZ) divide for longer than in other non-adjacent regions (thorax and terminus) (Fig. 2H).

To determine the division type of NB3-3 in each region, we used the following logic: if a lineage trace is initiated in a dividing GMC, the resulting clone will comprise two neurons (Notch ON/A; Notch OFF/B), regardless of induction time, and, of these, only the Notch OFF/B neuron will be detected by *EL-GAL4*. Thus, GMC clones should appear as a single cell (herein 1:0 clones) (Fig. 2E, bottom row). If, however, a lineage trace is initiated in a dividing neuroblast, the resulting clone will be large, with earlier inductions producing larger clones (Fig. 2E, diagonal from top left to bottom right). Therefore, the presence of 1:0 clones suggests that NB3-3 is dividing to generate a proliferative GMC, or that induction occurred during the final neuroblast division. However, we cannot exclude heterogeneity in GAL4 expression. We calculated the percentage of clones that were 1:0 at each heat-shock time and for each region (Fig. 2G). For the abdomen, for all but the latest induction time, the percentage of 1:0 clones was in the minority (Fig. 2E,G). This is consistent with the idea that NB3-3 is directly generating ELs rather than GMCs, which is expected based on previous work (Baumgardt et al., 2014). For the thorax and SEZ, most clones were 1:0 regardless of induction time (Fig. 2F). For the terminus, our data were ambiguous (Fig. 2F), most likely due to the small number of clones and ELs in this region. In broad strokes, these data fit a model in which NB3-3 in more anterior segments generate proliferative daughters, whereas those in more posterior segments (herein the abdomen) directly produce neurons.

Taken together therefore, these data suggest that the two major proliferation decisions – division type and division duration – are implemented combinatorially in the NB3-3 lineage to produce four different proliferation patterns, one in each CNS region. In the SEZ, we propose NB3-3s divide throughout neurogenesis, producing proliferative GMCs at each division and generating a lineage of ∼24 neurons, 12 Notch ON/A and 12 Notch OFF/B (Fig. 2H). In the thorax, NB3-3s divide only early in neurogenesis, producing proliferative GMCs at each division, thereby generating a lineage of ∼12 neurons, 6 Notch ON/A and 6 Notch OFF/B (Fig. 2H). In the abdomen, NB3-3s divide throughout embryogenesis, usually directly producing neurons. The lineage size is ∼12 neurons; all except one are Notch OFF/B ELs (Fig. 2H). In the terminus, we propose NB3-3s divide only early in neurogenesis (Fig. 2H). The lineage size might be ∼6 neurons, most of which are early-born Notch OFF/B ELs (Fig. 2H). We pose this as a general model that we tested with additional experiments.

Taking this model and extrapolating from what we know about NB3-3 temporal cohorts in segment A1 and the thorax, our data raise the possibility that, depending on the region, NB3-3 neuroblasts exhibit distinct proliferation patterns to generate different sets of temporal cohorts in each region (Fig. 2H). Specifically, the SEZ might be comprised of four temporal cohorts – early-born Notch OFF/B (ELs), early-born Notch ON/A, late-born Notch OFF/B (ELs) and late-born Notch ON/A (Fig. 2H). The thorax would have two temporal cohorts – early-born Notch OFF/B (ELs) and early-born Notch ON/A (Fig. 2H). We already know that A1, and likely the rest of the abdomen, has early-born Notch OFF/B and late-born Notch OFF/B temporal cohorts (Wang et al., 2022; Fig. 2H). In the terminus, there would only be an early-born Notch OFF/B temporal cohort (Fig. 2H). In this scenario, NB3-3 would have the early-born Notch OFF/B temporal cohort in every segment and combinations of other types of temporal cohorts on a region-by-region basis.

### NB3-3 neuroblasts produce different numbers of Notch ON/A neurons across regions

NB3-3 neuroblasts make EL and Notch ON/A neurons. So far, we have focused on ELs (Figs 1,2) and have inferred rather than demonstrated the presence of Notch ON/A neurons (Fig. 2). To begin to test the idea presented in Fig. 2H, we needed to directly examine the Notch ON/A neurons in each segment.

To count the number of Notch ON/A neurons, we stained for the neural Notch reporter, Hey (Monastirioti et al., 2010). Among all Hey-expressing neurons, we identified those in the NB3-3 lineage using a second lineage-tracing method, GAL4 Technique for Real-time And Clonal Expression (G-TRACE) (Evans et al., 2009) (Fig. 3A). In G-TRACE, transient GAL4 expression is converted into permanent nuclear-localized RFP labeling of descendant cells. We used the G-TRACE with *Eagle-GAL4*, which is transiently expressed in four classes of neuroblasts (Higashijima et al., 1996). The NB3-3 lineage can be identified by position in all segments except SEZ2 (Fig. 3C). In the *eg>G-TRACE* background, in addition to anti-Hey, we stained with a pan-neural marker, anti-ElaV, to distinguish neurons from neuroblasts, and with anti-Eve to confirm NB3-3 lineage identity (Fig. 3A,B). With this combination, we can identify Notch ON/A (RFP, ElaV, Hey) and Notch OFF/B (RFP, ElaV, Eve) neurons (Fig. 3A,B). SEZ3 possesses 12 Notch ON/A neurons, and the thorax possesses five or six Notch ON/A neurons (Fig. 3C). The number of Notch ON/A neurons in SEZ3-Th3 exactly matches the number of Notch OFF/B/EL neurons in those segments (Fig. 1E), supporting the idea that these NB3-3s produce GMCs on every division. In contrast, each segment in the abdomen and terminus possesses one Notch ON/A neuron (Fig. 3C), suggesting these NB3-3s only produce one GMC.

**Fig. 3.**
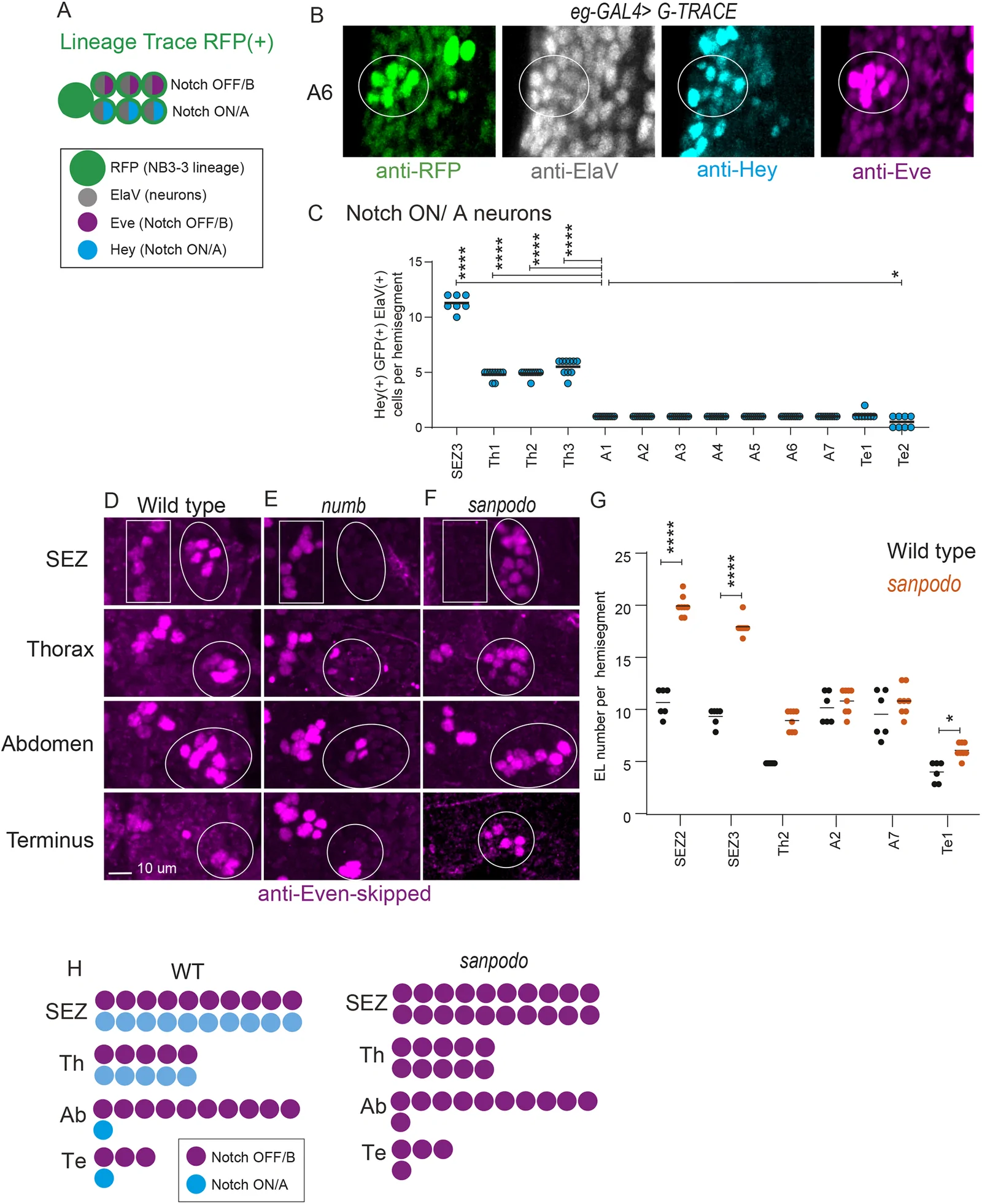
**In different segments, NB3-3 generates different numbers of Notch ON/A neurons.** (A) Illustration of NB3-3 lineage markers showing how the lineage is labeled using G-TRACE. (B) Images of a lineage-tracing experiment. The late embryonic nerve cord is shown with the anterior up. The circle denotes cells in the NB3-3 lineage. (C) Quantification of the number of Notch ON/A neurons at the end of embryonic neurogenesis. (D-F) Images of Notch pathway manipulations. Embryonic nerve cords are shown with anterior up. U motor neurons are boxed. EL neurons are circled. (G) Quantification of EL number in control and *sanpodo* mutants. (H) Illustration of Notch ON/A and Notch OFF/B neurons in the NB3-3 lineage in wild type and *sanpodo* mutants, organized by region. Each circle is a neuron. Black lines denote mean. Ordinary one-way ANOVA (****P<0.0001; *P<0.05). Ab, abdomen; SEZ, subesophageal zone; Te, terminus; Th, thorax.

We also assayed Notch ON/A neurons in the NB3-3 lineage by manipulating the Notch pathway, using *sanpodo* and *numb* mutants. In *sanpodo* mutants, all neurons adopt a Notch OFF/B fate, whereas in *numb* mutants they adopt the Notch ON/A fate (Skeath and Doe, 1998). We stained mutant embryos with Eve to assay phenotypes as in the previous work. First, these mutants functioned as previously reported: the Notch ON/A U motor neurons from the NB7-1 lineage were missing in *sanpodo* and doubled in *numb* (Fig. 3D-F) mutants. Next, we examined the NB3-3 lineage in mutants. Across segments, the Notch OFF/B ELs were nearly absent in *numb* mutants, confirming previous reports and extending them to the SEZ, thorax and terminus (Skeath and Doe, 1998) (Fig. 3E). In *sanpodo* mutants, the effect on EL number varied dramatically across regions, so we counted ELs (Fig. 3F,G). Also, the SEZ and thorax had nearly twice as many ELs as controls (Fig. 3F,G), which is consistent with a model in which all daughters of a GMC division become Notch OFF/B ELs, rather than half becoming Notch ON/B and the other half becoming Notch ON/A (Fig. 3H). In the abdomen and terminus, there was only a small increase in EL production (Fig. 3F,G), which is consistent with a model in which there is only one GCM division in these regions (Fig. 3H).

In summary, using two independent methods, we assayed Notch ON/A neurons in the NB3-3 lineage across segments. In the SEZ and thorax, NB3-3 neuroblasts make equal numbers of Notch OFF/B and Notch ON/A neurons, whereas in the abdomen and terminus, NB3-3 neuroblasts make one Notch ON/A neuron. These data provide support for the division patterns shown in the model in Fig. 2H.

### Temporal cohorts are molecularly similar, but not identical, across regions

The model in Fig. 4A shows differences in the numbers and types of neurons across regions and suggests that these neurons could be grouped into four temporal cohorts. Previous analysis of A1 showed that Kruppel (Kr) and Knot (Kn; also known as Collier) label many Notch OFF/B early-born ELs, and Lin29 and *11F02-GAL4* label most Notch OFF/B late-born ELs (Fig. 4A) (Heckscher et al., 2014, 2015; Tsuji et al., 2008; Demilly et al., 2011; Wreden et al., 2017). The early-born Notch OFF/B temporal cohort is found in all regions, but the late-born Notch OFF/B temporal cohort is found only in the SEZ and abdomen (Fig. 2H). Here, we asked if temporal cohorts are molecularly similar across regions, so we needed to assay the expression of the A1 temporal-cohort markers. First, we crossed *11F02-GAL4* to a nuclear-localized GFP and stained late-stage embryos with anti-GFP and anti-Eve (Fig. 4B). In the abdomen, *11F02-GAL4* labels a maximum of five or six ELs, depending on the segment (Fig. 4C). In the SEZ, ELs are organized into four clusters of approximately five or six neurons each (Fig. S2A), and each cluster could correspond to a temporal cohort. Consistent with this, one of the two EL clusters in SEZ3 was labeled by *11F02-GAL4* (Fig. 4C; Fig. S2D). Notably, 11F02 expression in SEZ2 was hard to assess. Thus, we counted Lin29 ELs across regions and found that SEZ2 had approximately four or five (Fig. 4D; Fig. S2E). Second, we counted the number of Kr-expressing ELs per hemisegment. Kr labeled between one and four ELs in all segments, except Te2 (Fig. 4E). Third, we found that Kn-expressing ELs were present in all segments (Fig. 4F).

**Fig. 4.**
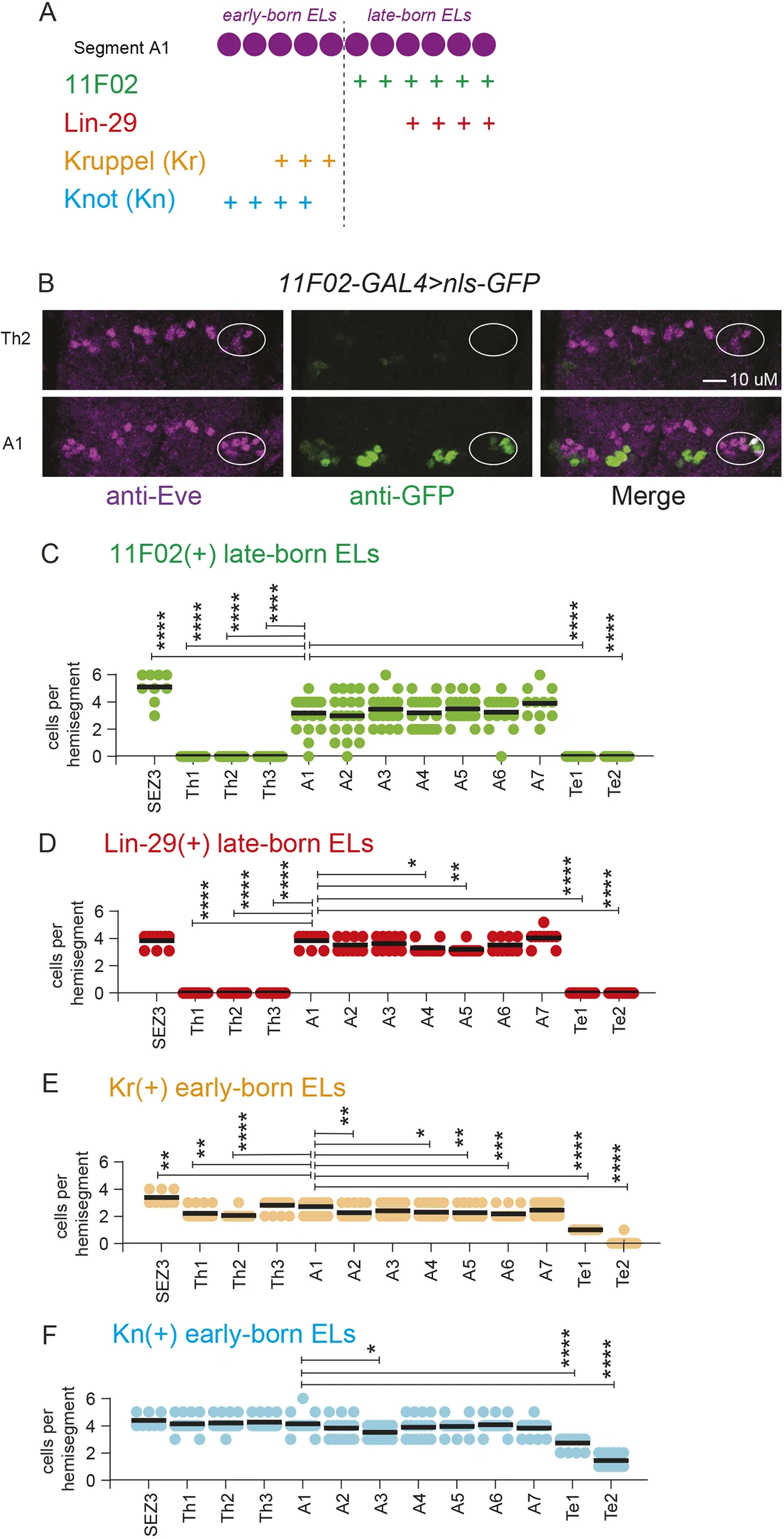
**Temporal cohort molecular markers are consistent across regions.** (A) Illustration of markers for early-born ELs and late-born ELs in segment A1. (B) Image of 11F02 expression in late-stage embryonic nerve cord with the anterior up. The EL neurons on the right side are circled in each image. (C-F) Quantification of the number of ELs expressing the given marker. Black lines denote mean. Ordinary one-way ANOVA (****P<0.0001; ***P<0.001; **P<0.01; *P<0.05).

In summary, our data support the idea that EL temporal cohorts across different regions are molecularly similar, but not identical. The observation that 11F02 does not label ELs in SEZ2 leaves room for molecular divergence of temporal cohorts across regions. Furthermore, the molecular differences in the number of Kr^+^ and Kn^+^ early-born ELs suggest that, within a temporal cohort, neurons are heterogeneous. Indeed, although previous connectome analysis showed statistically significant connectivity among early-born ELs of A1, the morphology of each early-born EL in A1 and its connectivity is distinct (Wang et al., 2022). Motivated by the observation of heterogeneous expression, we next probed for post-mitotic differences between the ELs across segments.

### Programmed cell death limits the number of Notch OFF/B neurons in terminal segments

Neuron number is regulated by a balance between proliferation and cell death. So far, we have examined neuronal production via proliferation. Here, we examine cell death. Cell death occurs within the NB3-3 lineage: blocking cell death increases the number of neurons in the NB3-3 lineage in segment Te2 (Birkholz et al., 2013). However, which cells die – NB3-3 neuroblast, Notch OFF/B and/or Notch ON/A neurons – and if cell death occurs in other regions, is unknown. We needed to understand the dynamic production of NB3-3 neurons and how manipulation of neural and neuroblast cell death alters EL number.

To assess the dynamics of neurogenesis in the NB3-3 lineage, we began by counting EL across segments starting at mid-neurogenesis (Fig. 5A-C). By stage 13, there were already five to seven ELs in all segments, except Te2, which had three ELs (Fig. 5D). Thus, most segments produce the neurons that populate the early-born EL temporal cohort. At stage 15, the EL number in the thorax and Te1 remained the same, but increased in the SEZ and abdomen (Fig. 5E). At stage 17, the EL number in the SEZ and abdomen continued to increase, the EL number in the thorax plateaued, and the EL number in Te1 and Te2 decreased (Fig. 5F). These counts show how the dynamics of neurogenesis vary by region, and represent the integration of proliferation and cell death.

**Fig. 5.**
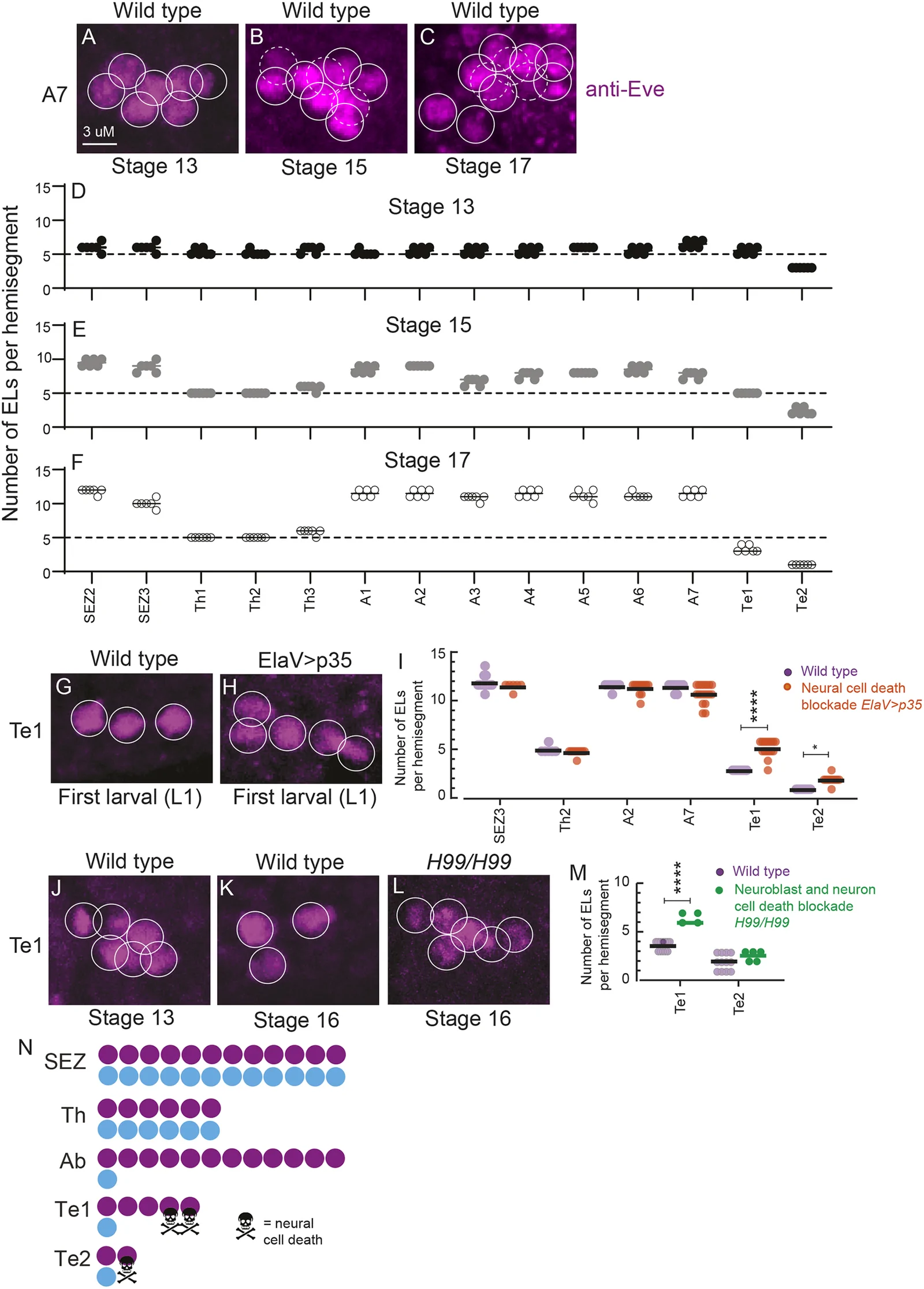
**A subset of ELs dies in the terminus.** (A-C) Images showing how EL number increases over embryonic neurogenesis. (D-F) Quantification of ELs in each segment. The dashed line at five ELs is shown for reference. (G,H) Images of larval nerve cords show an increase in EL number in the terminus with neural cell death blockade. (I) Quantification of the number of ELs comparing control to neural cell death blockade in representative hemisegments. (J-L) Images of ELs in the terminus, showing fewer at later stages (compare J and K) and when cell death is blocked using H99 (compare K and L). (M) Quantification of EL number in terminal segments comparing control to H99-mediated cell death blockade. (N) Illustration summarizing cell death of ELs. Each circle represents one cell. For images (A-C,G,H,J-L) each circle denotes one EL, with dashed circles indicating the ELs obscured by the image stack. For quantifications (I,M) black lines denote mean. Ordinary one-way ANOVA (****P<0.0001; *P<0.05). Ab, abdomen; SEZ, subesophageal zone; Te, terminus; Th, thorax.

We assayed neural cell death by inhibiting apoptosis using a pan-neural driver to drive the p35 Caspase inhibitor (Clem et al., 1991) (herein *Elav>p35*; Fig. 5G-I). We counted the larval EL number in representative segments in each region. For segments SEZ3-A7, there was no difference in EL number (Fig. 5I), indicating that once produced the ELs survive. For segments Te1 and Te2, the EL number significantly increased (Fig. 5I), indicating that two or three ELs are killed in each terminal hemisegment. We conclude that EL cell death is localized to the terminus.

Next, we assayed the contribution of neuroblast versus neural cell death to EL number. The logic was that if EL number increased substantially when both neuroblast and neural cell death were blocked, compared with neural blockade alone, then NB3-3 likely undergoes programmed cell death under wild-type conditions. Alternatively, if EL number is similar when both neuroblast and neural cell death were blocked compared to neural blockade only, then NB3-3 is unlikely to undergo programmed cell death in wild-type conditions. We blocked cell death in both neuroblasts and neurons by removing multiple cell death genes using the *Df(3L)H99* deletion. We counted the number of ELs in stage 16 embryos, because *H99* mutants do not survive to larval stages (Fig. 5J-M) (White et al., 1994). The effect of blocking both neuroblast and neural cell death was similar to blocking only neural cell death (Fig. 5M). Thus, the EL number in the terminus is primarily regulated by neural cell death.

Finally, we assayed Notch ON/A neurons in the terminus. To assess the dynamic production and loss of Notch ON/A, we counted neurons using the G-TRACE dataset. We compared two late-embryonic stages (i.e. stages 15 and 17) because this period is when Notch OFF/B/EL numbers are reduced (Fig. 5E,F). Notch ON/A neuron numbers were constant from segment Th1 to Te2 (Fig. 3C). In SEZ3, the number of Notch ON/A neurons increased (Fig. 3C), showing that neurons were still being added. Thus, there is no evidence that Notch ON/A neurons die.

We conclude that in the terminus, but not elsewhere, EL neurons are killed by programmed cell death after birth (Fig. 5N).

### In A1 and the terminus, a subset of early-born Notch OFF/B neurons fails to express Eve

Cell death is one of many cellular mechanisms that can limit the ability of a neuron to integrate into a circuit. Another mechanism that restricts circuit entry is that a neuron might fail to express post-mitotic identity genes, such as *eve*. Without Eve, EL neurons cannot fully differentiate or incorporate into circuits. *Drosophila*, *Caenorhabditis elegans* and mouse Eve homologs are required for proper neuronal morphogenesis (Moran-Rivard et al., 2001; Fujioka et al., 2003; Landgraf et al., 1999; Fetcho and McLean, 2010). Moreover, in the NB3-3 lineage, Eve expression is required for the sensory-processing functions of both early- and late-born ELs (Marshall and Heckscher, 2022). Hence, one mechanism limiting the ability of a neuron to become an EL and properly enter a circuit is failure to express Eve. There is a first-born Notch OFF/B neuron in segment A1 that is thought to fail to express Eve (Fig. 6A) (Tsuji et al., 2008; Wang et al., 2022). Therefore, we needed to assay Notch OFF/B neurons that fail to express Eve and their birth order across segments.

**Fig. 6.**
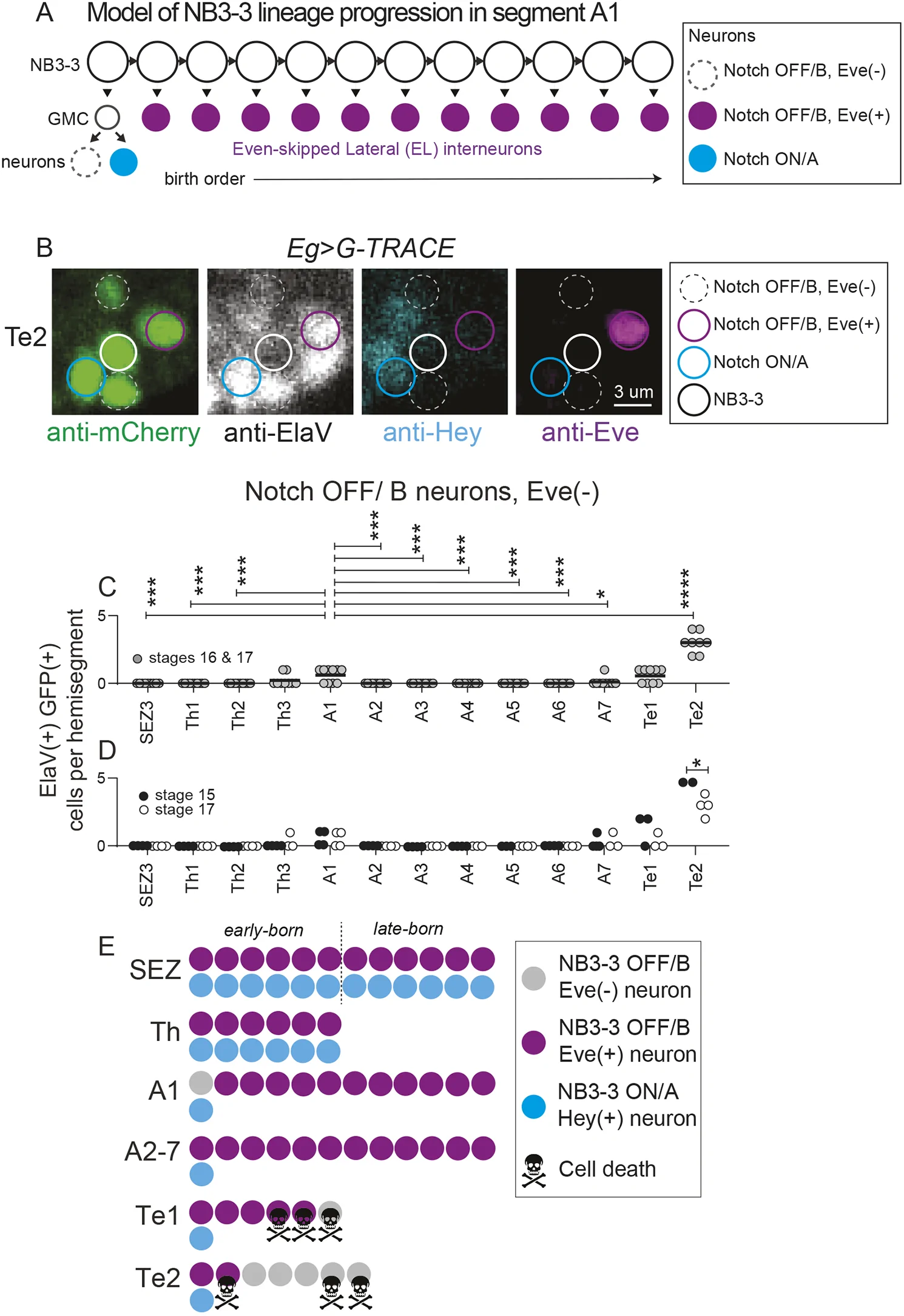
**In segments A1, Te1 and Te2, a subset of neurons in the early-born Notch OFF/B temporal cohort does not express Eve.** (A) Illustration of the model for NB3-3 lineage progression in segment A1. (B) Image of a G-TRACE experiment showing each circle corresponding to a neuron of the 3-3 lineage in the terminus. (C,D) Quantification of NB3-3B/OFF neurons that fail to express Eve. (C) Stages 16 and 17 were pooled and compared for statistical analysis. (D) Stages 15 and 17 are color-coded separately to show changes over time. (E) Illustration summarizing Notch OFF/B neurons, their Eve expression, and cell death. Black lines denote mean. Ordinary one-way ANOVA (****P<0.0001; ***P<0.001; *P<0.05). Ab, abdomen; GMC, ganglion mother cell; SEZ, subesophageal zone; Te, terminus; Th, thorax.

We used our G-TRACE dataset (Fig. 3A-C) to identify Notch OFF/B neurons that failed to express Eve (i.e. RFP^+^, ElaV^+^, Hey^−^, Eve^−^ cells− (Fig. 6B-E). In SEZ, thorax and most of the abdomen, all Notch OFF/B neurons expressed Eve (i.e. none failed to express Eve; Fig. 6C). In A1, most often, segments contained one Notch OFF/B neuron that failed to label with Eve (Fig. 6C). In the terminus, one to three Notch OFF/B neurons failed to express Eve (Fig. 6C). Thus, failure of Notch OFF/B neurons to become ELs due to lack of Eve expression occurs both in A1 and the terminus.

Because we had found cell death in the terminus (Fig. 5), we also asked whether Notch OFF/B Eve^−^ neurons die. We counted Notch OFF/B Eve^−^ neurons at stages 15 and 17. In the SEZ, thorax and abdomen, the Notch OFF/B Eve^−^ number was consistent (Fig. 6D), but in the terminus, it decreased by one to two neurons (Fig. 6D). Our data suggest that a subset of Notch OFF/B Eve^−^ neurons in the terminus dies (Fig. 6E).

Finally, we asked about the birth order of the neurons that fail to express Eve. In the terminus, NB3-3 produces only early-born Notch OFF/B, so, they must be within the early-born temporal cohort. For segment A1, the model (Fig. 6A) suggests that the first-born NB3-3 division generates Eve^−^ neurons, but the supporting evidence is weak. Our ts-MARCM data revealed a difference between NB3-3 lineages in A1 and A2-A7 (Fig. S3). When both A1 and another abdominal segment were labeled in a single CNS, a majority of the CNSs had smaller clones in A1 compared to A2-A7 (Fig. S3A). We also counted EL numbers in A1 and A2 in the CNS of developing WT embryos. A majority of embryonic CNSs had fewer ELs in A1 compared to A2 (Fig. S3B-E). These observations are consistent with the idea that in A1, the first-born Notch OFF/B neuron fails to express Eve, but in other abdominal segments the first-born Notch OFF/B neuron does express Eve (Fig. 6E).

### A subset of early-born ELs in the thorax fails to differentiate

A potential third cellular mechanism to restrict neurons from entering a circuit is that they could express identity genes but nevertheless fail to differentiate. In the *Drosophila* nerve cord, there are two morphological hallmarks of differentiated neurons. First, differentiated neurons have soma sizes >3 µm (Heckscher et al., 2014), whereas undifferentiated neurons are smaller (∼1.5 µm) (Lacin et al., 2009). Second, differentiated neurons form elaborate axon and dendritic arbors, whereas undifferentiated neurons have stick-like projections (Lacin et al., 2009; Wang et al., 2022). We visualized the morphology of NB3-3 neurons using our ts-MARCM data (Fig. 2). In the thorax, we found a small but consistent number of Eve-expressing ELs with small soma (Fig. 7A-C), which we never saw in other regions (Fig. 7D). To confirm that these neurons are undifferentiated, we characterized their arbor morphology, finding stick-like neurites (Fig. 7E,F). We conclude that, in the thorax, a subset of early-born Notch OFF/B ELs expresses Eve protein but fails to differentiate (Fig. 7G).

**Fig. 7.**
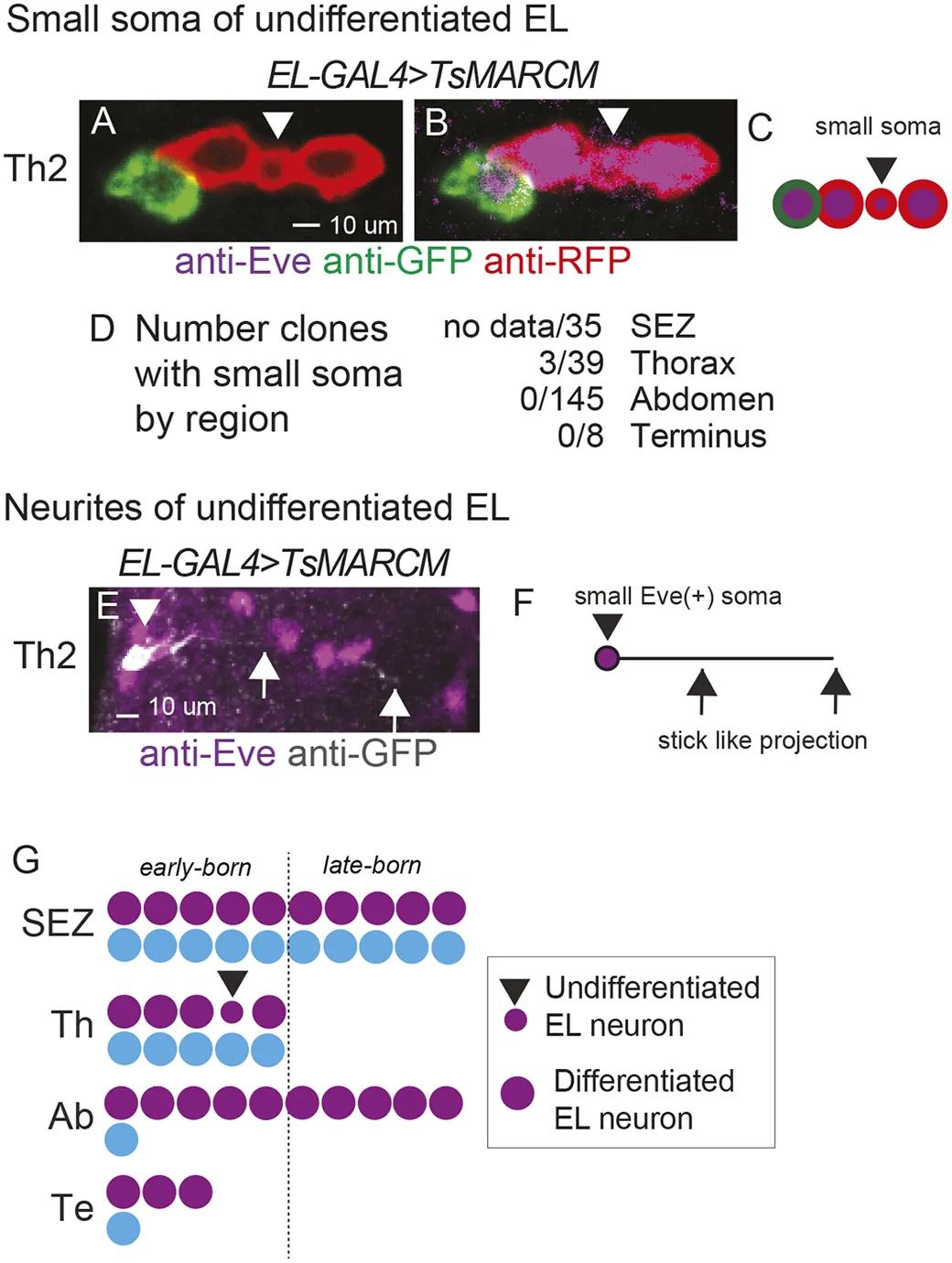
**In the thorax, a subset of ELs fails to differentiate.** (A-C) Images and an illustration of a thoracic clone containing a small soma (arrowhead). (D) Quantification of the distribution of clones with small soma size. (E,F) Image and illustration of a thoracic clone with a stick like neurite. (G) Illustration summarizing the distribution of undifferentiated neurons. Ab, abdomen; SEZ, subesophageal zone; Te, terminus; Th, thorax.

### Early-born ELs can contribute to different circuits depending on the region

Early-born Notch OFF/B ELs are present across all segments (Fig. 2). They express similar, but not identical, molecular markers across regions (Fig. 4), and a variety of post-mitotic mechanisms reduce the number of neurons that can enter circuits (Figs 5–7). This raises the question of how the circuit mapping of early-born ELs across segments is structured. We asked which circuits the remaining neurons in the early-born EL temporal cohort wire into in various regions. More specifically, we address the following: (1) In segment A1, all early-born ELs get direct synaptic input from chordotonal sensory neurons (CHOs) (Wang et al., 2022). Both CHOs and early-born ELs are found in every segment (Fig. 8A; Bodmer and Jan, 1987). To what extent do ELs in all segments receive synaptic input from CHOs? (2) In segment A1, all late-born ELs get direct synaptic input from various proprioceptive sensory neurons, including dorsal bi-polar dendrite (DBD) (Wang et al., 2022). We newly showed that DBD is present from segments Th2 to Te1 by analyzing the expression of the DBD line, *0165-GAL4* (Fig. 8A,B). However, late-born ELs are missing from the Th2, Th3 and Te1 (Fig. 2H). Combined, this raises the question: to what extent do ELs in different segments receive synaptic input from proprioceptors?

**Fig. 8.**
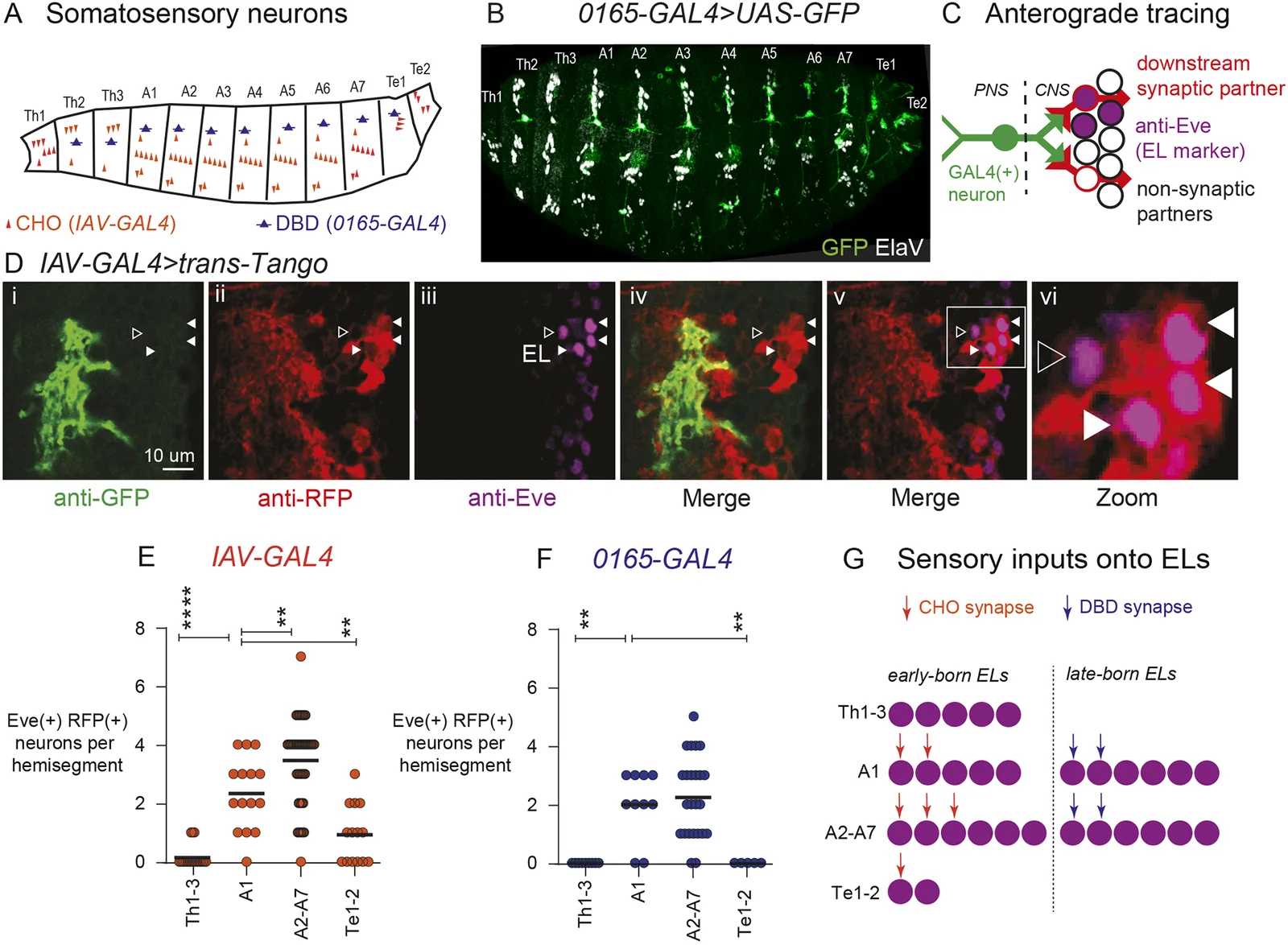
**In the abdomen and terminus, but not the thorax, ELs receive inputs from CHO and DBD neurons.** (A) Illustrations of larval sections and their sensory neuronal inputs. (B) Image of an embryo at stage 17 showing the peripheral nervous system (ElaV) with GFP-labeled DBD neurons. Anterior is to the left, and dorsal is up. (C) Illustration of the trans-Tango anterograde labeling system. Neurons that receive direct synaptic input from GAL4^+^ neurons (green) become RFP^+^ (red). Purple circles represent anti-Eve staining of nuclei of EL interneurons. (D) Image of an example trans-Tango experiment. A single abdominal hemisegment third-larval instar CNS is shown in dorsal view, with anterior up and midline to the left, showing RFP^+^ ELs (solid arrowheads) and RFP^−^ ELs (hollow arrowheads). The box in panel v is shown enlarged in vi. (E,F) Quantification of the number of ELs labeled by trans-Tango. (G) Illustration summarizing the inputs to ELs by region. Black lines denote mean. Ordinary one-way ANOVA (****P<0.0001; **P<0.01).

To identify somatosensory inputs onto ELs, we used the trans-Tango anterograde circuit tracing technique (Talay et al., 2017). In trans-Tango, the starting GAL4-expressing neurons are labeled with GFP, and their downstream synaptic partners are labeled with RFP (Fig. 8C,D). To identify downstream targets of CHOs and DBDs, we used *IAV-GAL4* and *0165-GAL4*, respectively (Marshall and Heckscher, 2022). We counted the number of RFP^+^ ELs in each segment to identify those that received input. First, we calibrated trans-Tango for use in ELs, focusing on segment A1, for which connectome data are available (Wang et al., 2022). For connectivity to CHOs, of the five early-born ELs in A1, three are strongly connected to CHOs (>15 synapses), two are weakly connected (<15 synapses) and late-born ELs are unconnected (0 synapses). In trans-Tango, in A1, approximately two ELs are labeled using *IAV-GAL4* (Fig. 8E). For connectivity to DBDs, of the six late-born ELs in A1, three are strongly connected to proprioceptors, three are weakly connected, and the early-born ELs are unconnected. In trans-Tango, in A1, approximately two ELs are labeled using *0165-GAL4* (Fig. 8F). Together, these data suggest that the trans-Tango method identifies ELs that are strongly synaptically connected to CHO or DBD neurons.

Next, we examined trans-synaptic labeling of CHOs to ELs from the thorax to the terminus. In Te1-Te2, trans-Tango labeled one EL (Fig. 8E). In A2-A7, trans-Tango labeled three ELs per segment, which is significantly higher than in A1 (Fig. 8E). Thus, more ELs may be strongly connected to CHO in A2-A7. In the thorax, trans-Tango tracing failed to label any ELs (Fig. 8E), suggesting an absence of strong synaptic connectivity.

We examined trans-synaptic labeling data of DBD to ELs from the thorax to the terminus*.* In the terminus and in the thorax, trans-Tango tracing from *0165-GAL4* did not label ELs (Fig. 8F). A lack of DBD-to-EL synapses might be expected given the lack of late-born ELs in those segments. In A1-A7, trans-Tango tracing labeled the same number of ELs in each segment (Fig. 8F). This result suggests that the number of strong DBD-to-EL synapses does not differ in segments where late-born ELs are present.

Our results are consistent with the following model (Fig. 8G): although CHO sensory neurons and early-born ELs are present in every segment, they are only strongly synaptically coupled in a subset of segments (i.e. within the abdomen and terminus). Across abdominal segments, the number of neurons receiving strong CHO input varies. DBD sensory neurons are present in segments Th2-Te1 (Fig. 8B), but only late-born ELs in the abdomen get their input. When synapses do occur, they are similar in coupling strength across abdominal segments. Although we did not examine inputs onto ELs in the SEZ, SEZ ELs are likely mapped to different circuits because the SEZ does not receive projections from CHO or DBD body wall somatosensory neurons. Most broadly, we find evidence that neurons in the early-born Notch OFF/B EL temporal cohort can be mapped into different circuits depending on segment.

## DISCUSSION

The objective of this study was to begin to understand the cellular mechanisms by which regionally specialized circuits are produced from serial homologous neuroblasts. We used NB3-3 lineages as a model because they generate EL neurons in every segment, yet EL numbers vary by more than an order of magnitude (Fig. 1), indicating that neurogenesis in this lineage is highly variable across regions. Across regions, NB3-3 neuroblasts vary in their division duration and division type in a combinatorial manner (Figs 2 and 3). The ultimate impact is that regional differences in neurogenesis enable NB3-3 neuroblasts to generate distinct sets of five to seven neurons, herein temporal cohorts (Figs 2–4). These sets of temporal cohorts include one type produced by NB3-3 in every segment (early-born Notch OFF/B neurons), which we term a fundamental temporal cohort, and from none to three other temporal cohorts. Indeed, NB3-3 neuroblasts must produce neurons of the fundamental temporal cohort if it is to make any other type of neuron. Uniquely, after their birth, subsets of neurons in the fundamental cohort are excluded from circuit membership (Figs 5–7). The cell-biological mechanisms underlying circuit elimination vary by region and include neuronal cell death (Fig. 5), failure to express identity markers (Fig. 6) and/or failure to differentiate (Fig. 7). Also uniquely, when neurons in the fundamental cohort to enter circuits, their membership can vary across regions (Fig. 8). These data are summarized in Fig. 9.

**Fig. 9.**
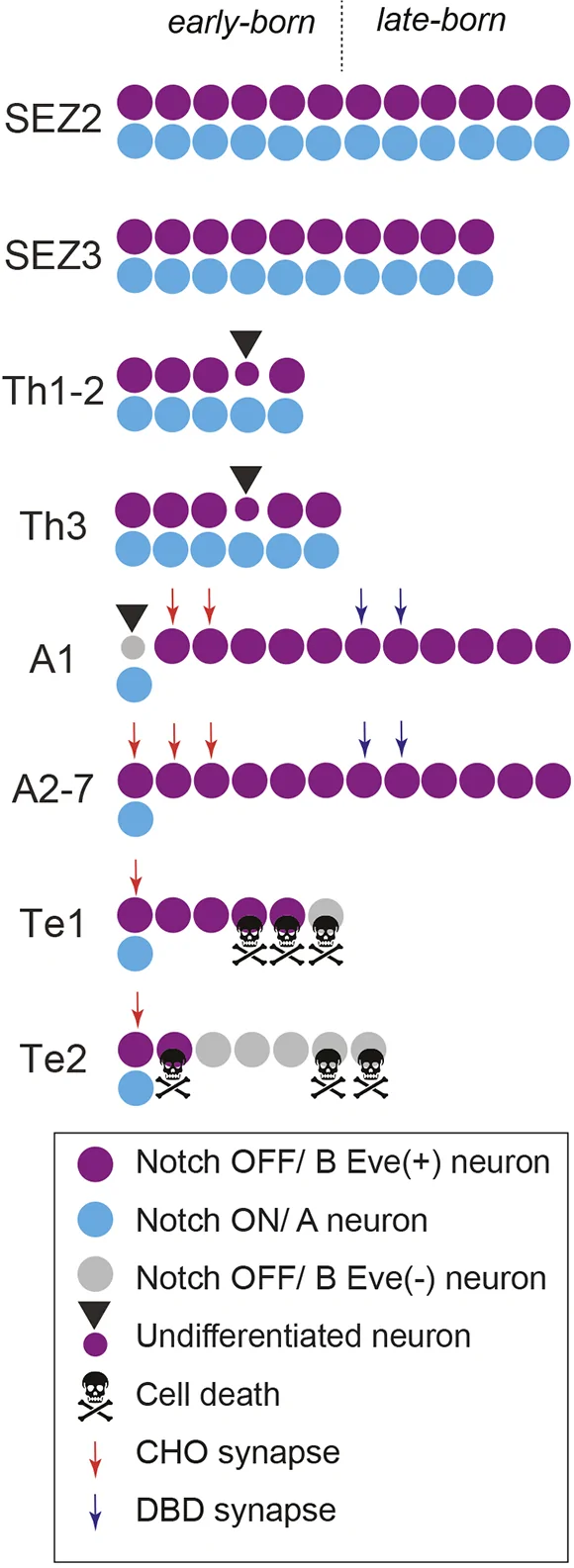
**Summary of NB3-3 lineage progression in the *Drosophila* nerve cord from anterior to posterior segments.** Illustration of neurons produced by NB3-3 in different regions of the body axis. Every circle represents one neuron and is quantitatively accurate with the number of NB3-3 progeny produced per hemisegment. A, abdomen; SEZ, subesophageal zone; Te, terminus; Th, thorax.

### To generate diverse circuits, serially homologous neuroblasts modulate their proliferation and modify neurons of the fundamental temporal cohort post-mitotically

Our data support a model in which two modes are used in parallel to enable serially homologous stem cells to generate regionally specialized circuits. The first mode involves varying proliferation patterns of the stem cell: neuroblasts alter proliferation patterns, adding or omitting temporal cohorts across regions, such that some regions have extra neurons that can be wired into specialized circuits.

The second mode occurs post-mitotically and is used only by neurons within the fundamental temporal cohort. Given that NB3-3 neuroblasts always make a fundamental temporal cohort, there were two possibilities about the impact for circuit wiring. First, neurons of a fundamental cohort might make a fundamental circuit, the function of which is similar in every segment. For example, in the spinal cord, neurons of the medial motor column are distributed along its length, always innervating axial musculature (Agalliu et al., 2009). Second, neurons of a fundamental temporal cohort might contribute to axially specialized circuits. Our data clearly support the second possibility (Figs 5–9). Thus, even neurons of the fundamental temporal cohort should be able to generate regionally specialized circuits.

### Serially homologous neuroblasts proliferate according to a multimodal production model

Stem cell proliferation is tightly regulated, as defects can cause microcephaly or megaloencephaly, tumors or neurodevelopmental disorders (Liszewska and Jaworski, 2018; Homem et al., 2015; Ossola and Kalebic, 2022). In theory, any stem cell has an extremely large number of possible proliferation patterns, with random and unitary production being two example patterns (Llorca et al., 2019; Gao et al., 2014). We newly show that the 14 NB3-3 serially homologous neuroblasts use a small subset of the possible proliferation patterns, producing numbers of neurons in multiples of five to seven. These data document a new multimodal production model.

### Relationships between Hox genes and axial specialization of NB3-3 lineage progression

Hox genes are major molecular regulators of patterning along the body axis and nervous system (Philippidou and Dasen, 2013; Estacio-Gómez and Díaz-Benjumea, 2014; Dasen et al., 2005; Tsuji et al., 2008; Cobeta et al., 2017). Broadly, *Drosophila* Hox expression domains correlate with variance in NB3-3 lineage progression (Fig. 9), and this simple model has been partially tested by other studies (Tsuji et al., 2008; Baumgardt et al., 2014; Gunnar et al., 2016; Birkholz et al., 2013). However, our data demonstrate that the NB3-3 lineage employs a complex combination of convergent and divergent cell-biological mechanisms, that cannot yet be explained by our understanding of Hox gene biology in this lineage. By converging, for example, Eve is not expressed in Notch OFF/B neurons in the Ubx^+^ A1, AbdB.m^+^ Te1, and AbdB.r^+^ Te2 segments. Such convergence correlates with the Hox-specificity paradox, in that different Hox genes likely have similar molecular targets. By diverging, for example, all Notch OFF/B neurons are viable in the Ubx^+^ A1 segment, but not in the AbdB.m^+^ and AbdB.r^+^ terminal segments. So, Ubx should not regulate the same repertoire of cell-biological processes as AbdB does, even though both likely promote programmed cell death in the NB3-3 lineage. Thus, other factors such as dynamic Hox expression, chromatin context, Hox co-factors or post-transcriptional regulation (Slattery et al., 2011; Mazzoni et al., 2013; Johnston and Desplan, 2008; Crocker et al., 2015) likely provide additional layers of lineage diversification.

### Conclusion and outlook

In summary, we identified cell-biological processes that act in both stem cells and neurons, generating different numbers of neurons and neural connectivities that can help diversify the neural circuits generated by serially homologous neuroblasts. The impact of this work is to advance our thinking on stem cell-based circuit assembly, surfacing concepts that can be tested next in other *Drosophila* lineages and other species. We note how the NB3-3 lineage exhibits trends observed in other phyla. (1) In the mouse spinal cord, there can be ∼10-fold differences in molecular neuronal subtypes (Sweeney et al., 2018). Similar fold changes are found in various neuron types in the NB3-3 lineage (Fig. 9). (2) In certain regions of the spinal cord, up to half the motor neurons are lost due to neural cell death (Okado and Oppenheim, 1984; Homma et al., 1994; Frade and Barde, 1999). In the terminus, half of the Notch OFF/B neurons from the NB3-3 lineage are lost due to neural cell death. Together, this work provides a framework for understanding the axially specialized development of serially homologous stem cells *in vivo.*

Flexible circuit development from homologous pools of stem cells occurs even more widely across biology. In many species, the initial pool of stem cells is identical in animals of different sexes, and yet, sexually dimorphic behavior requires specialized circuits (Tong et al., 2026). Frogs and chicks both have the same set of progenitors in the spinal cord (Fetcho and McLean, 2010), and yet, the circuits that control frog jumping or chick wing flapping must be highly specialized. Thus, our work provides a new framework for how developmental variation in stem cells might generate specialized behavior.

## MATERIALS AND METHODS

### *Drosophila* strains and culture

Fly stocks were maintained at 25°C on a standard cornmeal molasses medium. Experimental crosses were set up at the indicated temperatures. The fly lines used can be found in Table S4. Embryos and early-stage larvae were used. At these developmental stages, flies have no distinguishing sexual characteristics. Thus, all experiments were conducted in a manner that was unaware of sex.

### Tissue preparation

Three tissue preparations were used: (1) late-stage whole mount embryos, in which antibodies can still penetrate the cuticle; (2) late-stage dissected embryos, in which the neuromuscular tissue and cuticle are dissected away from the CNS, allowing for superb immuno-labeling and visualization of the entire axial nerve cord from SEZ to terminus; (3) isolated larval CNSs, in which the CNS is removed from other larval tissue so that antibodies reach the CNS. For all preparations, standard methods were used for fixation in fresh 3.7% formaldehyde (Sigma-Aldrich). Embryos were staged for imaging based on standard morphologic criteria.

### Immunohistochemistry

Tissue was washed three times with phosphate-buffered saline containing 0.1% Triton X-100 (PBT), blocked for 1 h in PBT containing 2% normal goat serum and stained with primary antibody in PBT at 4°C overnight. After washing, samples were stained with secondary antibody at room temperature for 1 h, washed again, and applied with an increasing ethanol series (30%, 50%, 70%, 95%, 100%) to replace PBT. Samples were then immersed into xylene for clearance and mounted in DPX (Sigma-Aldrich). Details of primary antisera used can be found in Table S5. Secondary antibodies were obtained from Jackson ImmunoResearch and were used at a 1:400 dilution (see Table S6).

### Hybridization chain reaction

Hybridization chain reaction was performed for the *lin-29* gene as described by Henderson et al. (2025). The oligonucleotide probes used are listed in Table S7. The *Drosophila lin-29* oligonucleotide sequences were generated as described by Kuehn et al. (2022), and are used with a B2-Alexa 564 amplifier (HCR version 3, Molecular Instruments).

### Imaging, statistics and figure assembly

Images were acquired either on a Zeiss 800 confocal microscope with a 40× objective or a Zeiss 880 with Airyscan processing. Images were processed and analyzed using ImageJ (FIJI). Test and descriptive statistical analyses were performed using Prism (GraphPad). Figures were assembled in Adobe Illustrator.

### Cell counts

This procedure is for counting cells in a *z*-stack, particularly cells in a cluster that are challenging to count by eye. Using the Multi-point tool in FIJI, it is possible to label each cell with a numbered point to show the presence of all cells in a cluster. This works well for images taken at 2048×2048 with ∼0.5 µm slices. (1) Open the .czi file in FIJI and select the channel of interest. (2) Go to Process/Enhance Contrast… and change Saturated Pixels to 0.1%. Select Normalize and Process all slices before pressing OK. (3) Go to Process/Noise/De-speckle to further process the image before analysis. (4) Save the changes as a .tif. (5) Open the .tif, scroll to the first slice where a cell appears, and zoom in on the cell cluster. Use the+and−keys to zoom to avoid moving points erroneously. (6) Select Multi-point in the FIJI toolbar and double-click to open the Point tool window. Set Type to Hybrid, Size to Tiny, and select Label points. Set counter to 1. Press OK. (7) With Multi-point tool selected, click on the center of a cell to drop a point. Scroll through the stack and drop a point on each slice until the cell is no longer visible. With slices of ∼0.5 µm cells should be present in 6-8 slices. (9) Return to the Point tool window and set counter to 2. Repeat to mark the remaining cells. (1) Notes specific to counting ELs: label U neurons to ensure that Us are not counted as ELs, using the 0 counter.

### ts-MARCM experiments

We used an internal reference to precisely estimate when heat shocks were provided to the embryos to induce recombinase expression and initiate the lineage trace. This is because heat shocks were provided to embryos for 2- to 3-h windows, it takes several hours for FLP to induce a genetic change (Wang et al., 2022) and neuroblasts divide every 45 min. This internal reference system works on the developmental principle that, unlike vertebrates and long-germ band insects, where axial development is temporally offset, with the anterior developing before the posterior, in short-germ band insects like *Drosophila*, segments develop synchronously. So, it is reasonable to assume that neuroblasts are dividing at approximately the same time along the anterior-posterior axis, and there are no differences between left and right hemisegments. We found that most CNSs had multiple clones (Fig. 2C). For each CNS, we assessed the location and size of each clone. Because we know NB3-3 divides for the duration of embryogenesis (Gunnar et al., 2016), the number of ELs labeled in the abdomen is directly related to the number of NB3-3 divisions yet to occur. If one or more clones were found within the abdomen, we used it to calculate the maximum abdominal clone size per CNS; using the maximum abdominal clone size serves as our internal reference. The maximum abdominal clone size is a proxy for the earliest time at which an embryo could have been heat-shocked. We sorted each clone into bins based on its maximum abdominal clone size in its CNS. This binning generates the *x*-axis in Fig. 2D-F and is labeled as ‘heat shock relative to inferred NB3-3 division number’.

We combined two datasets in this study (Fig. 2A). Although Twin Spot MARCM is a powerful, inducible lineage-tracing strategy, the data are inherently messy and can be challenging to interpret. Here, we analyzed a large ts-MARCM dataset comprising >200 clones. Every clone is listed in Table S1. Despite this large dataset, there are nonetheless some notable holes:

#### No experiments that included the SEZ were conducted before inferred division five.

We combined a published ts-MARCM dataset (Wang et al., 2022) with a new dataset. In the Wang dataset (Table S1, experiments a-h), all heat shock time points were included, and the CNS was imaged at high resolution. But imaging was carried out only for the nerve cord; thus, a limitation was the lack of SEZ clones. So, we generated a second dataset (Table S1, experiments i-k) in which both the SEZ and the nerve cord were imaged. As these experiments are labor-intensive, we focused our efforts on the later time points, using heat shocks that began in the NB3-3 fifth division. Consequently, Fig. 2 does not have any data for SEZ before inferred division 5, whereas there is data in other regions. This was a strategic decision because we were trying to distinguish between two possible scenarios: (1) In the SEZ, NB3-3 divided early, made GMCs, but both daughters expressed Eve. (2) In the SEZ, NB3-3 divides throughout embryonic neurogenesis, giving rise to GCMs, with only the Notch OFF daughters expressing Eve. Only late heat shocks were needed to distinguish between these possibilities. Our data support scenario 2.

#### Uneven sampling across time points

Because of the imprecise nature of ts-MARCM, our experiments do not have even clonal coverage across developmental time points. (1) For example, the latest time point-inferred division 12 was not sampled. In Fig. 2E, one might expect the 12 divisions to be 100% 1:0 clones in the abdomen; however, we cannot definitively say this. (2) Ideally, the analysis in Fig. 2F, which estimates the timing of neuroblast divisions, should rely only on (large) neuroblast clones but not (1:0) GMC clones. Because the timing of division for neuroblasts and GMCs is different, with GMCs dividing after their neuroblast parent, including both might lead to a longer estimate of the neuroblast proliferation window. Unfortunately, we did not have enough data to analyze with only neuroblast clones. So, our analysis method could overrepresent the division time by one cell division. (3) Fig. 2F shows that a thoracic clone is produced at the inferred 11th NB3-3 division, which is unexpected based on the idea that NB3-3 goes into quiescence mid-embryonic neurogenesis (Tsuji et al., 2008). The clone in question is an *n*=1 observation (Table S1, row f). We found that the cell in the thoracic clone is morphologically similar to ELs in A1, suggesting it is a bona fide EL. We have no way of determining whether it was derived from a GMC division. This may be produced by infrequent reactivation of the thoracic NB3-3 division or a technical anomaly. To address this, we tracked EL number over developmental time (stages 13, 15, 17) for segments SEZ3 to Te2 (Fig. 5). In this secondary dataset, we do not see an increase in EL number in any thoracic segment after stage 13 (i.e. after the production of early-born ELs). Thus, we think the one late-born thoracic EL is a technical anomaly.

#### We excluded the clones that were SEZ-, thorax- or terminus-only from the analysis in Fig. 2

Because we used clone size in the abdomen to estimate the timing of neuroblast division (see Materials and Methods), we excluded a small subset of our data from the analysis. We have no reason to believe these clones differ from those in the analyzed dataset.

#### Stochasticity of heat-shock-induced recombination

In ts-MARCM, heat shocks are applied to developing embryos to induce recombinase expression in a temporally controlled manner. However, different cells, depending on size, location and age, may experience temperature differently in both timing and amplitude compared with others. Further, there is a degree of spatial heterogeneity, as heat exposure will NOT induce recombinase in every cell. Stochastic recombinase expression helps generate sparse clones, which are easier to analyze. However, ts-MARCM experiments are both temporally and spatially imprecise, leading to ‘noise’ in clonal production.

#### Variably effective RNAi

ts-MARCM relies on RNAi cassettes to knock down GFP and RFP protein expression in cells that have not undergone recombination. The original ts-MARCM paper reports that RNAi can be regulated differently in GMCs versus neuroblasts (Yu et al., 2009). One way this might influence our results is that, outside the abdomen, large clones produced by neuroblast recombination are rare; instead, many GMCs appear to be recombined.

#### Idiosyncrasies of the GAL4 line used to detect recombination events

ts-MARCM uses GAL4 lines to detect recombination events. Thus, clonal production is only as good as the GAL4 line: heat-shock-induced recombination timing sets the upper limit on the number of cells within a clone. However, if the GAL4 driver detects only a subset of cells within a clone, one would see artificially small clones. One way this might influence our results is that, to detect ts-MARCM recombination we used the *EL-GAL4* driver. The *EL-GAL4* driver is a small enhancer fragment (∼400 kb), meaning it does not capture the full regulatory context of the *eve* locus. In our experience (e.g. Manning et al., 2012), drivers using small enhancers tend to give highly specific, but somewhat variable, expression, and *EL-GAL4* sometimes labels only a subset of ELs. Thus, in some cases, clones detected with *EL-GAL4* may be smaller than expected, for example, having multiple (i.e. 1:0) clones in divisions 3 and 4 is not precisely what would be predicted by the model in Fig. 2E. Many of the assertions we made rely on ts-MARCM data combined with other data, both published and in this article.

#### In the SEZ, NB3-3 neuroblasts continually generate GMCs

In Fig. 2F, we see that in the SEZ, the majority of clones in inferred divisions 6-11 are 1:0, indicating NB3-3 divided to produce a GMC. Because there are no SEZ clones from inferred divisions 1-5, we must use other data to determine whether earlier NB3-3 divisions give rise to GMCs. First, we note that Gunnar et al. (2016) suggests that, in general, lineages transition from making GMCs early in lineage progression and only later switch to directly producing neurons. If this logic applies to the NB3-3 lineage in the SEZ, then NB3-3 should produce GMCs on every division. In support of that idea, our G-TRACE (Fig. 3C) and EL counts (Fig. 1E) show that the number of Notch ON/A neurons in SEZ3 is equal to the number of ELs in that segment, which is expected if NB3-3 produces a GMC on every division. Second, in *sanpodo* mutants, the number of ELs in the SEZ doubles, as it does in the thorax (Fig. 3G). These data also support the idea that in the SEZ NB3-3 neuroblasts usually produce both Notch OFF/B and Notch ON/A neurons in wild type, and that in *sanpodo* mutant Notch ON/A neurons express Eve.

#### In the abdomen, NB3-3 directly generates Notch OFF/B cells that assume EL identity for all but one of their divisions

In our ts-MARCM, at most inferred division times, NB3-3 in the abdomen produces both large (neuroblast) and small (1:0 GMC) clones; however, large clones are produced at most time points. This implies that NB3-3 directly generates neurons rather than GMCs in most divisions. In agreement with this, our G-TRACE data (Fig. 3C) show that only one NB3-3 Notch ON/A neuron is produced in each abdominal segment, suggesting that only one GMC is present during lineage progression. This is further supported by *sanpodo* mutant analysis (Fig. 3D-G). However, Fig. 3C does not show localization of the GMC to any particular division. The GMC could be generated either randomly or at a reproducible time throughout lineage progression. According to Baumgardt et al. (2014), in the abdomen, NB3-3 should generate one GMC in the first division and, starting in the second division, directly generate Notch OFF/B neurons.

### Use of artificial intelligence tools

PhoenixAI (The University of Chicago's Generative AI platform) was used to shorten the abstract to the word limit of 180 words.

## Supplementary Material



10.1242/develop.205389_sup1 Supplementary information

Table S6. Secondary antibodies

Table S7. *lin-29* HCR oligos
