# Supplementary material for: How serially homologous neuroblasts produce different temporal cohorts along the Drosophila larval body axis
Source: Development. 2026 Oct 12;153(19):dev205389. doi: 10.1242/dev.205389 (PMC13723392; doi:10.1242/dev.205389)
Supplement: Supplementary file 1 [file develop-153-205389-s1.pdf]

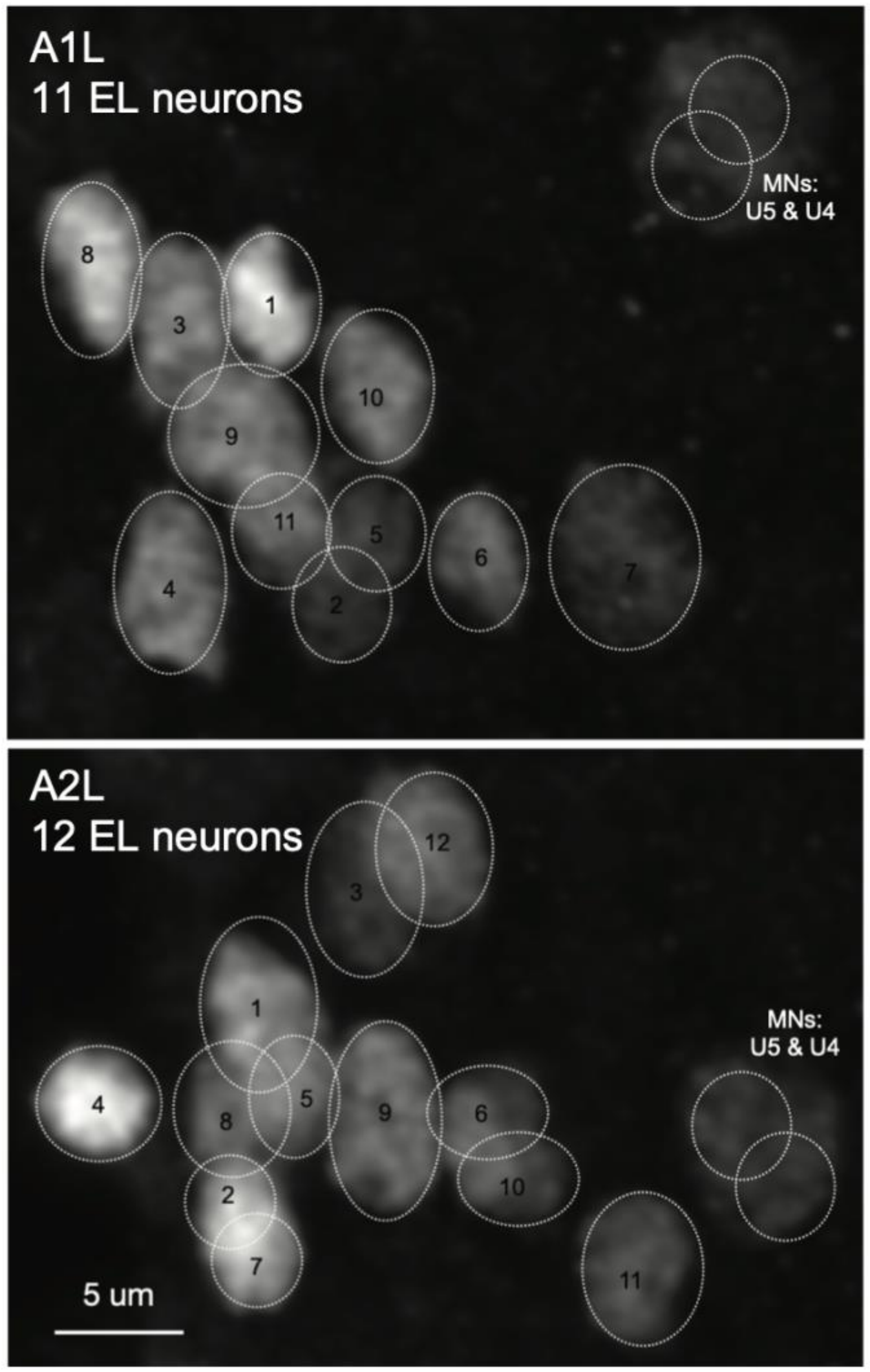

Fig. S1. At the larval stage, there are 12 ELs in segment A2 compared to 11 in A1.

Images of laterally-located Eve(+) neurons in segments A1 and A2 of a larval CNS taken with high-resolution Airyscan microscopy. Images are Z-projections of the confocal stack stained with anti-Eve antibody. Each neuron is outlined by a circle. Two U motor neurons are located medially (right in these images). Each EL is numbered with lower numbers representing more neurons located more dorsally within the cluster. In segment A1, there are 11 EL neurons, and in segment A2, there are 12 EL neurons.

Development • Supplementary information

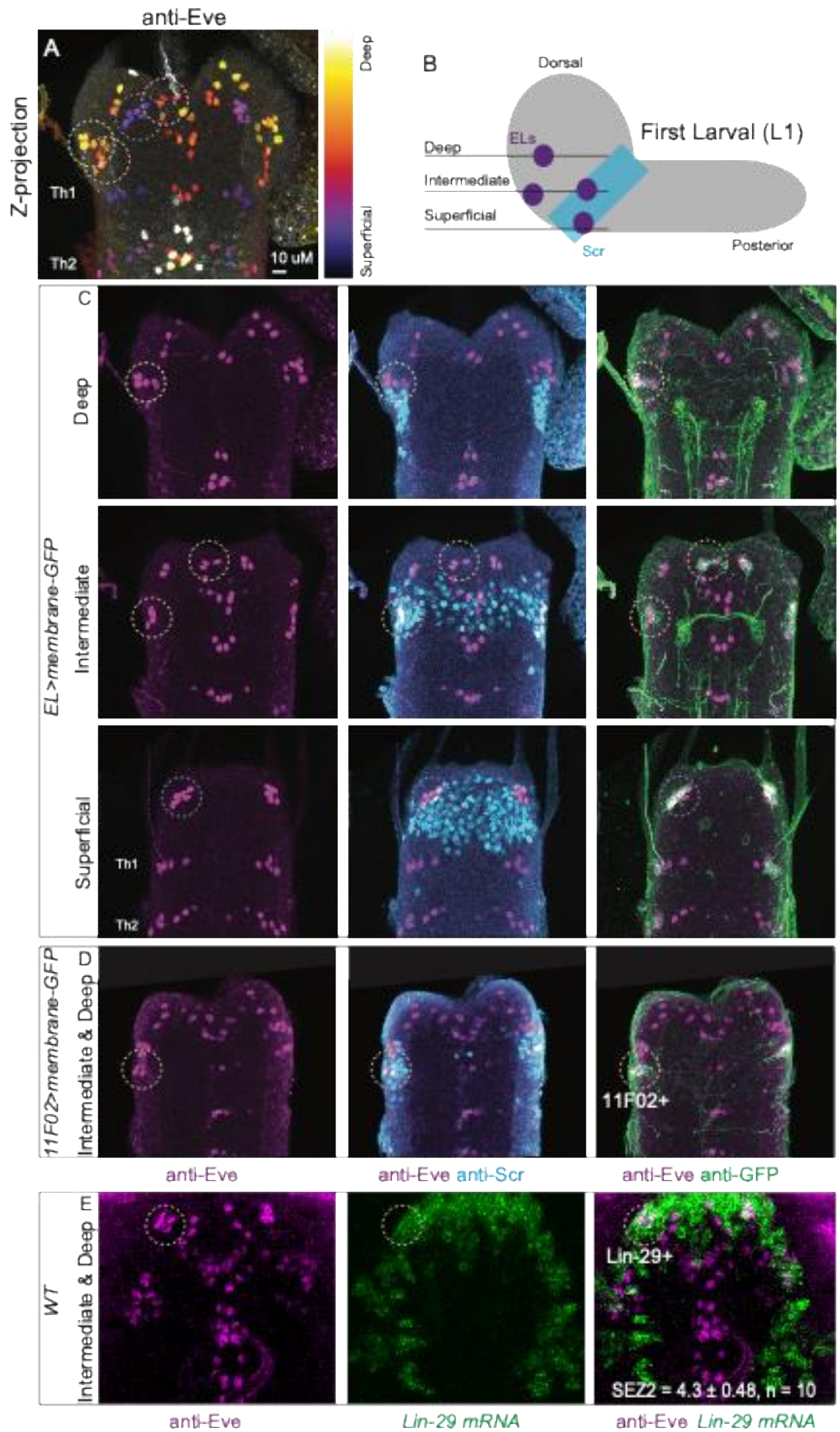

Development • Supplementary information

Fig. S2. There are four clusters of ELs in the SEZ (A, C-D) Images of Eve(+) neurons of the SEZ and thorax. The dashed circles show the ELs on the left side of the SEZ.

(A) The image is a depth-coded Z-projection of anti-Eve staining. Warm colors (yellow) represent deeper slices, and cool (purple) colors represent superficial slices. Th1 and Th2 are the first two segments of the thorax. (B) Illustration of the CNS with approximate positions of EL clusters within the SEZ. (C-D) Eve is in magenta. Scr, in cyan, marks the SEZ-nerve cord border (Diao et al., 2024). Green shows anti-GFP staining. (C) The same CNS as in (A). GFP marks the ELs with *EL-GAL4>UAS-CD4-GFP*. (D) GFP marks 11F02(+) (late-born) neurons with *11F02-GAL4>UAS-CD4-GFP*. (C) Projections of the larval CNS's superficial, intermediate, and deep thirds. (D) Projection of the intermediate and deep thirds of the late-stage embryonic SEZ and thorax. (E) Images of the SEZ, with dashed circle showing ELs in SEZ2. Eve is in magenta and Lin-29 mRNA in in green.

Development • Supplementary information

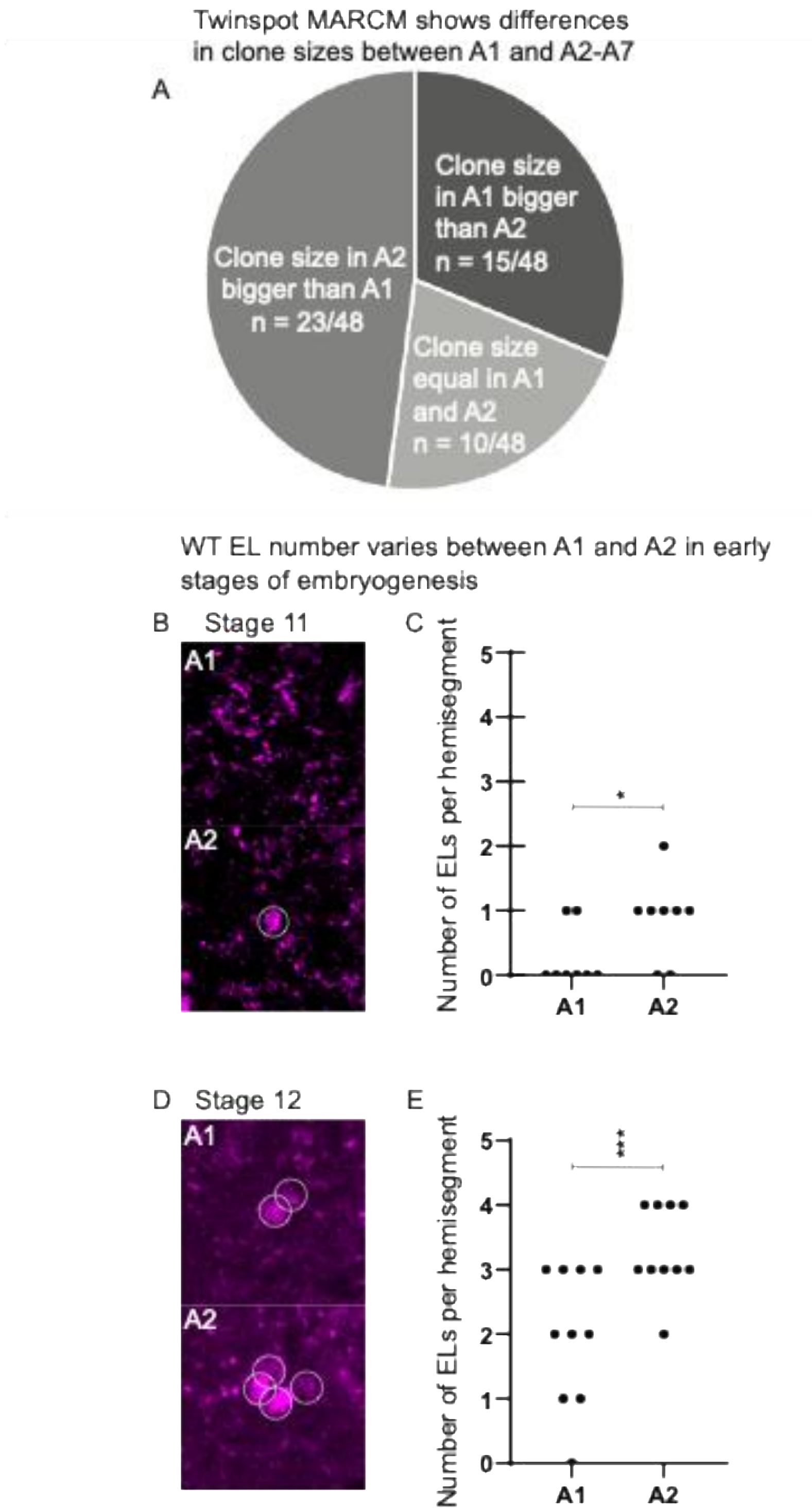

Fig. S3. (A) Pie chart showing instances of clone size differences in segments A1 compared to segments A2-A7. (B, C) Image and quantification of ELs in A1 and A2 at stage 11. (C, D) Image and quantification of ELs in A1 and A2 at stage 12.

Table S1. Clones per CNS

Heat shock protocol	CNS identifier	Segment and clone compostion												
		SEZ 2&3	T1	T2	T3	A1	A2	A3	A4	A5	A6	A7	A8	A9
a	Jan23-2	ND	1:1			3:1	1:1	1:1						
a	Jan23-7	ND		3:1		1:0	6:1							1:0
a	Jan23-1	ND					1:0	6:1		5:1				
a	Jan23-5	ND				1:1			2:1					
a	Jan23-4	ND										4:1, 4:1		
a	Jan23-8	ND				5:1			5:1					
a	Jan23-3	ND				1:0, 2:1		2:1			4:1			
a	Jan23-6	ND				10:1								
b	Jan27-4	ND		1:0	1:0		7:1				6:1	5:1		
b	Jan27-5	ND		1:0	1:0	7:1			1:0					
b	Jan27-6	ND			1:0	7:1	1:0			1:0	8:1			
b	Jan27-1	ND				10:1				8:0				
b	Jan27-2	ND				10:1								
b	Jan27-3	ND				2:1								
c	Feb3-1	ND			3:1	11:1							2:1, 1:0	1:0
c	Feb3-3	ND			3:1						8:1			
c	Feb3-9	ND											1:0	
c	Feb3-8	ND					5:1			5:1				
c	Feb3-6	ND						6:1						
c	Feb3-7	ND									7:1			
c	Feb3-2	ND						8:1						
c	Feb3-4	ND				8:1								
c	Feb3-5	ND							8:1					
d	Nov22-2	ND	1:0	1:0										
d	Nov22-4	ND					2:1				1:0	2:1		
d	Nov22-5	ND							2:1					
d	Nov22-3	ND				1:0, 2:1		2:1	1:1	3:1				
e	Jan12-1	ND				4:1								
f	Jan16-1	ND		1:0		5:1		8:1						
f	Jan20-3	ND			1:0	1:0			1:0			9:1		
f	Jan20-4	ND			1:0	8:1				9:1				
f	Jan20-2	ND			1:0				10:1	10:1	11:0	1:0		
f	Jan20-1	ND			3:1				5:1		1:0			
f	Jan19-2	ND								1:1		1:1		
f	Jan19-1	ND				3:1								
f	Jan19-3	ND						4:1						
f	Jan19-5	ND				5:1								
g	Nov22-3	ND	1:0		1:0									
g	Nov22-6	ND		1:0				1:0						
g	Nov22-10	ND			1:0						6:1			
g	Nov22-7	ND										7:1	1:1	
g	Nov22-5	ND				3:1						1:0		
g	Nov22-8	ND							5:1					
g	Nov18-1	ND					6:1							
h	Jan10-3	ND				3:1								
h	Jan10-4	ND				1:1			2:1	3:1				
h	Jan10-5	ND										4:1		
h	Jan10-2	ND					5:1							
h	Jan10-6	ND						5:1						
h	Jan10-1	ND					6:1							
i	Mar2-2-10	ND			1:0		1:1							
i	Mar2-2-8	1:0		1:0										
i	Mar2-2-3	1:1							1:0					
i	Mar2-2-13	1:0, 2:2				1:0								
i	Mar2-2-20	1:0, 1:0				1:1		1:1	1:0	1:0				
i	Mar2-2-21	1:0, 1:0				3:1				2:1	2:1	2:1		
i	Mar2-2-7	1:0				1:1	2:1				2:1			

i	Mar2-2-9					1:0, 1:1				1:0	1:0	2:1		
i	Mar2-2-12	1:0					2:1							
i	Mar2-2-19							2:1		2:1		2:1, 1:0		
i	Mar2-2-1	1:4, 1:0							3:1					
i	Mar2-2-2							3:1		3:1	3:1	3:1		
i	Mar2-2-5	1:0, 1:0					3:1		1:0					
i	Mar2-2-14	1:0				1:1					1:1	3:1, 2:1		
i	Mar2-2-4	1:1							1:0	2:1, 4:1				
i	Mar2-2-11	1:0						4:1	4:1					
i	Mar2-2-16	1:0							4:1	3:1				
i	Mar2-2-18	1:0, 1:0, 1:0				3:1			3:1, 4:1					
i	Mar2-2-15	1:0									5:1			
i	Mar2-2-6	1:0, 1:0					2:1	5:1						
j	Nov18-2	ND				2:1						4:1		
j	Nov26-2	ND										5:1		
k	Mar2-1-7		1:0	2:1	1:0						9:1, 1:0	9:1, 1:0		
k	Mar2-1-6	1:0, 4:1	1:0	2:1				5:1						
k	Mar2-1-8	2:0	1:0	3:1	1:1	1:0		1:0			8:1			
k	Mar2-1-5	1:0	2:0, 1:0	1:0	1:0, 1:0									1:0, 1:0
k	Mar2-1-2	1:0, 1:0		1:1	2:1				5:1				1:1	
k	Mar2-1-3	1:0, 1:0, 1:0		2:1, 1:0							1:0			
k	Mar2-1-4	5:0, 1:0			2:1	1:1			6:1					
k	Mar2-1-1	1:0, 1:1				5:2				4:1				

Table S2.Ts-MARCM dataset

Heat shock protocol	Time of heat shock (minutes post fertilization) start	stop	CNSs	Clones number
a	300	480	8	22
b	315	495	6	18
c	345	495	9	15
d	360	480	4	11
e	360	540	1	1
f	375	495	9	23
g	420	540	7	12
h	420	645	6	8
i	480	600	2	3
j	420	480	20	73
k	540	600	8	48

Table S3. Complete genotypes by figure

**Figure #**	**Genotypes**
Figure 1	w1118
Figure 2	*hsFLP; UAS-rCD2.RFP, UAS-GFPi, FRT40A/ UAS-mCD8-GFP, UAS-rCD2i; EL-GAL4/ pPL[KD]*
Figure 3	*UAS-RedStinger, UAS-FLP, Ubi-FRT.STOP-Stinger/+; Eagle-GAL4/+*
Figure 4	*R11F02-GAL4/UAS-nls-GFP*
Figure 5	(A-G, J, K): wild-type (w1118)<br>(H-I): *ElaV-GAL4/+; UAS-p35/+*<br>(L-M): *Df(3L)H99/Df(3L)H99*
Figure 6	*UAS-RedStinger, UAS-FLP, Ubi-FRT.STOP-Stinger/+; Eagle-GAL4/+*
Figure 7	*hsFLP; UAS-rCD2.RFP, UAS-GFPi, FRT40A/ UAS-mCD8-GFP, UAS-rCD2i; EL-GAL4/ pPL[KD]*
Figure 8	(B): *UAS-GFP/+; 0165-GAL4/+*<br>(D, E): *UAS-myrGFP.QUAS-mtdTomato-3xHA; trans-Tango/IAV-GAL4*<br>(F): *UAS-myrGFP.QUAS-mtdTomato-3xHA; trans-Tango/+; 0165-GAL4/+*

Development • Supplementary information

Table S4. *Drosophila* lines used.

Name	Genotype	Location	Source
Wild type	w1118	X	(Marshall and Heckscher 2022) (Heckscher et al. 2015)
H99	Df(3L)H99	3	BDSC #1576
p35	UAS-p35	2	BDSC #5072
Elav-GAL4	Elav-GAL4	X	BDSC #5144
G-TRACE	P{w[+mC]=UAS-RedStinger}4, P{w[+mC]=UAS-FLP.D}JD1, P{w[+mC]=Ubi-p63E(FRT.STOP)Stinger}9F6	2	Evans et al., 2009; BDSC #28280
EL-GAL4	EL-GAL4	3	Heckscher et al., 2015
Twin Spot MARCM	UAS-rCD2.RFP.UAS-GFPi/CyO; TM3/TM6B	2	BDSC #56184
Twin Spot MARCM	UAS-mCD8.GFP.UAS-rCD2i/CyO; TM3/TM6B	2	BDSC #56185
11F02-GAL4	P{R11F02-Gal4 (w+)}attP2	3	BDSC #49828
UAS-nls-GFP	P{UAS-GFP::lacZ.nls}30.1, (w+)	3	BDSC #6452
UAS-CD4-GFP	y[1] w[*]; P{w[+mC]=UAS-CD4-tdGFP}8M2	2	BDSC #35839

Development • Supplementary information

TransTango	y[1] w[*] P{y[+t7.7] w[+mC]=UAS-myrGFP.QUAS-mtdTomato-3xHA}su(Hw)attP8; P{y[+t7.7] w[+mC]=trans-Tango}attP40	1,2	Talay et al., 2017; BDSC #77124
IAV-GAL4	IAV-GAL4	2	BDSC #36360
0165-GAL4	0165-GAL4	3	Marshall et al., 2022
sanpodo	+/+ ; Spdo-c55 / TM6B, Tb, Sb, Hu, DFD-YFP	2	BDSC #4374 rebalanced by the Doe lab
numb	+/+; P{hsneo}numb[1]/ CyO	2	BDSC #4096

Development • Supplementary information

Table S5. Primary antibodies used.

Antibody	Species	Dilution factor	Source
anti-Eve	rabbit	1:1000	Meng, et al., 2019
anti-GFP	guinea pig	1:2000	Rick Fehon, UChicago
anti-mCherry	chicken	1:1000	Novus NBP2-25158
anti-Hey	guinea pig	1:1000	Christos Delidikis, University of Crete
anti-Scr	Mouse	1:400	DSHB
anti-Kruppel	guinea pig	1:500	John Reinitz, UChicago
anti-Knot	guinea pig	1:500	Adrian Moore, Riken Center for Brain Science
anti-ElaV	rat	1:25	DHSB

Table S6. Secondary antibodies

Available for download at
https://journals.biologists.com/dev/article-lookup/doi/10.1242/dev.205389#supplementary-data

Table S7. *lin-29* HCR oligos

Available for download at
https://journals.biologists.com/dev/article-lookup/doi/10.1242/dev.205389#supplementary-data
