# Supplementary material for: How serially homologous neuroblasts produce different temporal cohorts along the Drosophila larval body axis
Source: Development. 2026 Oct 12;153(19):dev205389. doi: 10.1242/dev.205389 (PMC13723392; doi:10.1242/dev.205389)
Supplement: Supplementary file 2 [file develop-153-205389_review_history.pdf]

# How serially homologous neuroblasts produce different temporal cohorts along the *Drosophila* larval body axis

Deeptha Vasudevan, Yi-wen Wang, Hannah Carr, Elise Paniel, Sean Corcoran, Chris C. Wreden, Elaine Kushkowski, Conor Lee-Smith and Ellie S. Heckscher
DOI: 10.1242/dev.205389

**Editor**: James Briscoe

**Review timeline**

| | |
|---|---|
| Submission to Review Commons: | 23 July 2025 |
| Submission to Development: | 20 November 2025 |
| Editorial decision: | 25 November 2025 |
| First revision received: | 2 April 2026 |
| Editorial decision: | 12 May 2026 |
| Second revision received: | 17 August 2026 |
| Accepted: | 18 August 2026 |


---

**Reviewer 1**

**Evidence, reproducibility and clarity**

Summary: The study by Vasudevan et al intends to address how serially homologous neural progenitors generate different numbers and types of neurons depending on their neuronal progeny making it extremely difficult in vertebrate paradigm. The authors used NB3-3 in the developing fly embryo as a model to investigate the full extent of the flexibility in neurogenesis from a single type of serially homologous stem cell. Previous work showed NB3-3 generates neurons including lateral interneurons that can be positively labeled by Even-skipped, but detailed characterization of the NB3-3 lineage mainly focused on 3 segments during embryogenesis. The authors defined the number of EL neurons in all segments of the central nervous system in early larvae after the completion of circuit formation and carried out clonal analyses to determine the proliferation pattern of NB3-3. They described the failure to express Eve in Notch OFF/B neurons as a new mechanism for controlling the number of EL neurons and PCD limits EL neurons in terminal segments.

Major comments: The authors performed careful analyses of the NB3-3 lineage using EL neurons. My main concerns are limited applicability of their findings and lack of mechanisms as how NB3-3 generate various numbers of EL neurons. Their findings are exclusively relevant to the NB3-3 lineage despite their effort in highlighting that other NB lineages also generate temporal cohorts of EL neurons. I disagreed with their conclusion that failure to express Eve as a mechanism for controlling EL neuron numbers when Eve serves as the marker for these neurons. Are there any other strategy to assess the fates and functions of these cells beside relying solely on Eve expression? I am not familiar with the significance of Eve expression on the functions of these neurons. Is it possible to perform clonal analyses of NB3-3 mutant for Eve and see if these neurons adopt different functionalities/identities? If NB3-3 in the SEZ continually generate GMCs based on the interpretation of clonal analyses and depicted in Fig. 2A, why is the percent of clones that are 1:0 virtually at or near 100% from division 6-11 shown in 2G? The authors also indicate that NB3-3 in the abdomen directly generate Notch OFF/B cells that assume EL neuronal identity. In this scenario, shouldn't the percent of 1:0 clones be 100% in later divisions in Fig. 2G? Based on the number of clones in abdomen shown in Fig. 2E, I cannot seem to understand how the authors come to the percent of 1:0 clones shown in Fig. 2G

© 2026. Published by The Company of Biologists under the terms of the Creative Commons Attribution License (https://creativecommons.org/licenses/by/4.0/).

There are many potentially interesting questions related to this study that can significantly broaden the impact of this study. For example, are other NB lineages that also generate distinct temporal cohorts of EL neurons display similar proliferation patterns (type 1 division in SEZ, early termination of cell division in thoracic segments and type 0 division in abdomen)? Why does NB3-3 in the thoracic segment become quiescence so much sooner than SEZ and abdominal segments? The authors' observations suggest that NB3-3 in SEZ and abdomen generate a similar number of EL neurons despite the difference in their division patterns (type 1 vs type 0). Are the mechanisms that promote EL neuron generate in NB3-3 in SEZ and abdomen the same? Anything else is known beside Notch OFF?

Minor comments:

The authors' writing style is highly unusual especially in the result section. There is an overwhelming large amount of background information in the result section but very thin description on their observations. The background information portion also includes previously published observations. Since the nature of this study is not hypothesis-driven, it is very confusing to read in many places and difficult to distinguish their original observations from previously published results and making. One easily achievable improvement is to insert relevant figure numbers into the text more often.

**Significance**

The study by Vasudevan et al intends to address how serially homologous neural progenitors generate different numbers and types of neurons depending on their location along the body axis. Investigation of full repertoire of neurogenesis for these progenitors necessitates a precise ability to track the fates of both progenitors and their neuronal progeny making it extremely difficult in vertebrate paradigm. The authors used NB3-3 in the developing fly embryo as a model to investigate the full extent of the flexibility in neurogenesis from a single type of serially homologous stem cell. Previous work showed NB3-3 generates neurons including lateral interneurons that can be positively labeled by Even-skipped, but detailed characterization of the NB3-3 lineage mainly focused on 3 segments during embryogenesis. The authors defined the number of EL neurons in all segments of the central nervous system in early larvae after the completion of circuit formation and carried out clonal analyses to determine the proliferation pattern of NB3-3. They described the failure to express Eve in Notch OFF/B neurons as a new mechanism for controlling the number of EL neurons and PCD limits EL neurons in terminal segments.

**Reviewer 3**

**Evidence, reproducibility and clarity**

In this manuscript, Vasudevan et al provide a detailed characterisation of the different numbers and temporal birthdates of Even-skipped Lateral (EL) neurons produced at in different segments from the same neuroblast, NB3-3. The work highlights the differences in EL neuronal generation across segments is achieved through a combination of different division patterns, failure to upregulate EL marker Eve and segment-specific program cell death. For neurons born within the same window and segment, the authors describe additional heterogeneity in their circuit formation. The work underscores the large diversity that the same neuroblast can generate across segments.

Major comments:

- Based on the ts-MARCM 1:0 clones representing 100% of the SEZ clones at any given inferred cell division, the authors conclude "NB3-3 neuroblasts generate proliferative daughter GMCs in the SEZ and thorax on most divisions". Figure 2G does not have any data for SEZ before inferred division 5, whereas there is data in other regions. The authors also state "In the SEZ and abdomen, ELs were labelled regardless of induction time." In reference to Fig 2F, which seems inaccurate given there are no SEZ clones before inferred division 5.
There is no comment on this fact, which is surprising give their focus on temporal cohorts.

© 2026. Published by The Company of Biologists under the terms of the Creative Commons Attribution License (https://creativecommons.org/licenses/by/4.0/).

The authors should explain this discrepancy, if known, or modify their statements to reflect the data.
- The temporal cohort (early-born vs late-born) identity is exclusively examined based on markers. Given the absence of SEZ clones from early NB3-3 divisions, a time course showing that the SEZ generate early-born EIs or some other complementary method would be desirable.
- The authors repeatedly refer to their work as showing how a stem cell type can have "flexibility". Flexibility would imply that NB3-3 from one segment could adopt a different behaviour (different division pattern, or cell death or connectivity) if it were placed in a different segment. This is not what is being shown. In my opinion, "heterogeneity" of the same neuroblast across different segments would be more appropriate.

Minor comments:

- Figure 2A depicts a combination of known data and conclusions from their own (mainly SEZ). The authors might consider editing the figure to highlight what is new. A possibility would be for figure A to be a diagram of the experimental design and their summary division pattern to be shown after the new data instead of being panel A.
- The authors state that they combined published ts-MARCM with their new one, which differed in a number ways that they list, but they don't specify which limitations are associated with the published vs new dataset. Could the authors please clarify?
- The title refers exclusively to "temporal cohorts", which in the manuscript are defined quite narrowly and do not seem to apply to all segments.
- Several cited references are missing from the Reference list at the end. Could the authors please double check this? (e.g. Matsushita, 1997; Sweeney et al., 2018)
- Legend for figure 2 is a bit confusing, there is a "(A)" within the legend for (D), which indicates that segments A1-A7 are shown (this seems inaccurate, as it only goes to A6).

**Significance**

This study provides a comprehensive analysis of different cell biological scenarios for a neuroblast to generate distinct progeny across repeating axial units. The strength is the detailed and systematic approach across segments and possible scenarios: different division patterns, cell death, molecular marker expression. While it focuses on one specific neuroblast of the ventral nerve cord of Drosophila, the authors have done extensive work to place their findings and interpretation in the context of other cell types and across model organisms both in the introduction and discussion. This makes the work of interest for developmental biologists in general, neurodevelopment research in particular and those interested in circuit assembly, beyond their specialised community. This point of view comes from someone working in vertebrate CNS development.

**Reviewer 4**

**Evidence, reproducibility and clarity**

Summary

This manuscript addresses the question of how the number of neurons produced by each progenitor in the nervous system is determined. To address this question the authors use the Drosophila embryo model. They focus on a single type of neural stem cell (neuroblast), with homologues in each hemisegment along the anterior-posterior axis.

Using a combination of clonal labelling, antibody stainings, and blockade of programmed cell death, they provide a detailed description of segment-specific differences in the proliferation patterns of these neuroblasts, as well as in the fate and survival of their neuronal progeny. Furthermore, by employing trans-synaptic labelling, they demonstrate that neurons derived from the same progenitor type receive distinct patterns of synaptic input depending on their segmental origin, in part due to their temporal window origin.
Overall this work shows that different mechanisms contribute to the final number and identity of the neuronal progeny arising from a single progenitor, even within homologous progenitors along

© 2026. Published by The Company of Biologists under the terms of the Creative Commons Attribution License (https://creativecommons.org/licenses/by/4.0/). 

the anterior posterior body axis.

Major Comments

I would suggest adding line numbers to the text for future submissions, this massively helps providing comments.

The authors propose that all neuroblasts produce the same type of temporal cohort (early born) and that, by changing the pattern of cell division, different temporal cohorts can be added. The way this this presented in the abstract sounds like an obvious thing, what would be the alternative scenario/s? Here it's the late born neurons that lack in thoracic segments because of early NB quiescence, but it cannot be excluded that different neuroblast types adopt a different strategy.

I found the ts-MARCM results confusing for 2 reasons:

1. It's not clear to me why there are so many single cell clones in div 3 and 4 in abdominal segments. This is not compatible with the division model depicted for abdominal segments, unless GMCs are produced in those division window and the MARCM hits the GMC, as also mentioned in the legend for G. This aspect is important because, either the previous model by Baumgardt et al. - please correct cit. currently Gunnar et al. 2026 - is wrong, or something strange happens in this experiment, or the relative temporal order is incorrect.
2. In segments other than abdomen, it is quite rare to hit proper clones, it appears that only GMCs are hit by recombination, with very few exceptions. Could the author please provide an explanation for this or at least mention this aspect?

It is also unclear whether in F the graph includes all types of clones (including 1:0 clones). This is important, because the timing of division for NBs and GMCs is different, and inclusion of 1:0 might lead to a wrong estimate of the NB proliferation window (longer than it actually is because GMCs divide for longer). This is particularly important for the SEZ, where most clones in normalised division 10 and 11 are with ratio 1:0, thus compatible with both terminal division as well as GMC division.

To obtain an estimate of the timing of division, the authors normalise clone size to the size of the bigger clone in the abdomen. What happened to those samples where no abdominal clones were hit? Were they simply excluded from the analysis?

It is proposed that in the thorax late temporal cohort neurons are not produced, yet the ts-MARCM experiment detects some 1:0 clones. What is the fate of these cells? Are they all derived from GMC division and therefore decoupled from the temporal identity window? Or is this a re-activation of division?

"in A1, a majority of segments had one Notch OFF/B neuron that failed to label with Eve" does "the majority" in this sentence mean that there were cases where all B neurons were labelled with Eve? If yes, where would this stochasticity come from? Additionally, there is no evidence that it's the first born NotchOFF neuron in A1 that does not express Eve. The authors should clarify where this speculation comes from.

When discussing trends shared with other phyla:

A- "In the mammalian spinal cord, more neurons are present in regions that control limbs (Francius et al., 2013). Analogously, EL numbers do not smoothly taper from anterior to posterior; instead, the largest number of ELs is found in two non-adjacent regions, SEZ and the abdomen." It's unclear what is the link between the figure in the mammalian spinal cord and the Drosophila embryo. The embryo doesn't even have limbs and the number of neurons measured here refer only to a single lineage, while there could be (and in fact there are) lineage-to-lineage differences that could depict a different scenario.

B- The parallelism between V1 mouse neurons and EL Drosophila neurons is also unclear to me. The similarity in fold change across segments could be a pure coincidence and, from what I understand, the two cell types are not functionally linked.

© 2026. Published by The Company of Biologists under the terms of the Creative Commons Attribution License (https://creativecommons.org/licenses/by/4.0/).

Minor comments:

I found the manuscript somewhat difficult to follow, even though I am familiar with both the model and the topic. For non-specialist readers, I expect it will be even more challenging. The presentation of the results often feels fragmented, at times resembling a sequence of brief statements rather than a continuous narrative. I would encourage the authors to provide more synthesis and interpretation, for example by summarising key findings, rather than listing in detail the number of neurons labelled in each segment for every experiment. This would make the results more accessible and easier to digest.

From the way the MS is written it's not clear from the beginning that the work focuses exclusively on embryonic-born neurons. Since in Drosophila neuronal stem cells undergo two rounds of neurogenesis, one in the embryo and one in the larva, this omission could lead to confusion.

In the abstract, what would be the other temporal cohorts generated in specific regions? (ref to: "In specific regions, NB3-3 neuroblasts produce additional types of temporal cohorts, including but not limited to the late-born EL temporal cohort.")

This sentence in the introduction is inaccurate: "The Drosophila CNS is organized into an anterior hindbrain-like subesophageal zone (SEZ) and a posterior spinal cord-like nerve cord". The anterior hindbrain-like portion of the CNS is in fact the supraesophageal ganglion (or cerebrum), while the SEZ is a posterior-like region.

Fig 1E: the encoding of the significance is not immediately clear. In the legend the 4 stars could also be arranged in the same way for clarity.

Fig 2E legend: it is mentioned that B corresponds to a 1:4 clone, however the MARCM example is shown for C and it's a 1:5.

The occurrence of "undifferentiated" neurons in Th segments is in less than 10% of the clones, I wonder if this a stochastic or deterministic event and to what extent small cell bodies could just be the consequence of local differences in tissue architecture.

Fig 2I: it's unclear what the purple means (I suppose it might be Eve expression) and why in J there should be one purple cell not labelled by the ts-MARCM when this is not present in H and I.

"When synapses do occur, they are numerically similar from segment to segment". It's unclear where the evidence for this statement comes from, please clarify or remove the sentence.

"In SEZ2, NB3-3 divides 10 times (Figure 2F)". Figure 2F does not support this statement and Figure 7 shows 12 divisions. Possibly SEZ2 and 3 have been inverted in this statement, please clarify.

Referees cross-commenting

I agree with most of the comments/suggestions provided by the other two reviewers. In particular:
I agree with reviewer #1's comment about failure to express Eve being a mechanism for controlling neurons number, as this is a circular argument.
I agree with reviewer #2's concern about the use of the word "flexibility"; "heterogeneity" would be a more appropriate term, as I would associate the word "flexibility" to the ability of a single neuroblast in a single segment to produce neurons with different fates under, for example, unusual growth conditions. Here no genetic/epigenetic manipulations were performed to address flexibility and the observed (stereotypical) differences result from axial patterning.
As a note, Reviewer #1 asks about other temporal cohorts of EL neurons produced by other lineages, but these neurons are specifically generated from NB3-3. To generalise the observations reported in this study, the authors would need to focus on other molecularly defined temporal cohorts or, more generally, on other lineages, which, however, are likely to adopt different combinations of mecahnisms to tune progeny number across segments.

© 2026. Published by The Company of Biologists under the terms of the Creative Commons Attribution License (https://creativecommons.org/licenses/by/4.0/).

**Significance**

In Drosophila melanogaster, the relationship between neural progenitors and their neuronal progeny has been studied in great detail. This work has provided a comprehensive description of the number of progenitors present in each embryonic segment, their molecular identities, the number of neurons they produce, and the temporal transcriptional cascades that couple progenitor temporal identity to neuronal fate.
This work adds to the existing knowledge a detailed characterisation of intersegmental differences in the pattern of proliferation of a single type of neuronal progenitor as well as in post-divisional fate depending on anterior-posterior position in the body axis (i.e. programmed cell death and Notch signalling activation). This is a first step towards understanding the cellular and molecular mechanisms underlying such differences, but it's not disclosing them.

That homologous neuroblasts can generate variable numbers of progeny neurons depending on their segmental position has been established previously. What this manuscript adds is the demonstration that these differences arise through a combination of altered division patterns and differential programmed cell death, thereby revealing a more complex and less predictable scenario than could have been anticipated from existing knowledge in other contexts. The advance provided by this study is therefore incremental, refining rather than overturning our understanding of how segmental diversity in neuroblast lineages is achieved. By touching on the general question of how progenitors generate diversity, this work could be of broad interest to developmental neuroscientists beyond the fly field. However, the way it is currently written does not make it very accessible to non-specialists.

My expertise: Drosophila neurodevelopment, nerve cord, cell types specification

---

**Author response to reviewers' comments**

**Manuscript number:** RC-2025-03130
**Corresponding author(s):** Ellie S. Heckscher

*[The "revision plan" should delineate the revisions that authors intend to carry out in response to the points raised by the referees. It also provides the authors with the opportunity to explain their view of the paper and of the referee reports.*

*The document is important for the editors of affiliate journals when they make a first decision on the transferred manuscript. It will also be useful to readers of the reprint and help them to obtain a balanced view of the paper.*

*If you wish to submit a full revision, please use our "Full Revision" template. **It is important to use the appropriate template to clearly inform the editors of your intentions**.]*

1. General Statements [optional]

We thank all three reviewers for their feedback on the paper. Reviewers stated that the paper was of broad interest to developmental biologists and neurobiologists. However, we want to ensure that our two key conceptual contributions are clear. We clarify in the following paragraph and include a revised abstract. We will update the introduction and paper to better reflect these advances. We also attach a supplemental table 1, which was inadvertently omitted from the previous submission due to our error.

The first advance is that serially homologous neuroblasts follow a multimodal production model: In principle, stem cells can divide any number of times, from once to throughout the entire lifetime

© 2026. Published by The Company of Biologists under the terms of the Creative Commons Attribution License (https://creativecommons.org/licenses/by/4.0/). 

of the animal. And, on each division, a stem cell can generate either a proliferative daughter cell or a post-mitotic neuron. Together, therefore, there is a vast potential number of neurons any given stem cell could produce. From the literature on the vertebrate neocortex, we had the following models: (1) “random production” model, in which any number of neurons could be made by a stem cell; or (2) “unitary production” model, in which the same number of neurons (~eight) is produced by a stem cell regardless of context. Our data revealed an entirely new “multi-modal production” model, which could not have been predicted by prior literature. In the context of serially homologous neuroblasts arrayed along the *Drosophila* larval body axis, sets of five to seven neurons are produced in increments of one, two, or four. These increments correspond to units called temporal cohorts. Temporal cohorts are lineage fragments, or small set of neurons that share synaptic partners, making them lineage-based units of circuit assembly. Thus, in a multimodal production model, serially homologous stem cells produce different numbers of temporal cohorts depending on location. Our data advance the field by showing that stem cells produce circuit-relevant sets of neurons by adding or omitting temporal cohorts from a region, to meet regional needs.

Key to understanding the second advance is that there are multiple types of temporal cohorts: early-born Notch OFF, early-born Notch ON, late-born Notch OFF, and late-born

Notch ON. One temporal cohort type, the early-born Notch OFF, is found in every segment, which we term the “ubiquitous” temporal cohort. The other temporal cohort types can be produced in various combinations depending on the stem cell division pattern and segmental location. In a result that could not have been predicted, we found that the ubiquitous temporal cohorts are refined both in terms of the number of neurons and their connectivity, depending on body region. In contrast, when other temporal cohort types are produced, they are not refined to the same degree.

The impact of this work is to advance how we think about stem cell-based circuit assembly.

2. Description of the planned revisions

*Reviewer #1 (Evidence, reproducibility and clarity (Required)):*

*Summary: The study by Vasudevan et al intends to address how serially homologous neural progenitors generate different numbers and types of neurons depending on their location along the body axis.*
*Investigation of full repertoire of neurogenesis for these progenitors necessitates a precise ability to track the fates of both progenitors and their neuronal progeny making it extremely difficult in vertebrate paradigm. The authors used NB3-3 in the developing fly embryo as a model to investigate the full extent of the flexibility in neurogenesis from a single type of serially homologous stem cell. Previous work showed NB3-3 generates neurons including lateral interneurons that can be positively labeled by Even-skipped, but detailed characterization of the NB3-3 lineage mainly focused on 3 segments during embryogenesis. The authors defined the number of EL neurons in all segments of the central nervous system in early larvae after the completion of circuit formation and carried out clonal analyses to determine the proliferation pattern of NB3-3. They described the failure to express Eve in Notch OFF/B neurons as a new mechanism for controlling the number of EL neurons and PCD limits EL neurons in terminal segments.*

Thank you! In addition to the contributions highlighted by the reviewer, we also showed that all segments have ELs with early-born molecular identities, but only a subset have ELs with late-born identities (Figure 5). And we showed that early-born temporal cohorts can be mapped into different circuits depending on the axial region (Figure 6).

*Major comments: The authors performed careful analyses of the NB3-3 lineage using EL neurons. My main concerns are limited applicability of their findings and lack of mechanisms as how NB3-3 generate various numbers of EL neurons. Their findings are exclusively relevant to the NB3-3 lineage despite their effort in highlighting that other NB lineages also generate temporal cohorts of EL neurons.*

Thank you for raising these points. First, to clarify, as Reviewer 4 also mentioned, NB3-3 is the

© 2026. Published by The Company of Biologists under the terms of the Creative Commons Attribution License (https://creativecommons.org/licenses/by/4.0/). 

only lineage to produce EL neurons. We will ensure that this is clearly stated in the revised text.

We agree that our findings might not apply beyond the NB3-3 lineage. However, as this is the first study of its kind, it is impossible to know a priori to what extent the concepts surfaced here are generalizable. In our opinion, this speaks to the novelty and impact of the study. A contribution is to motivate a need for future studies. We will make this explicit in our updated manuscript in the Discussion section.

Our manuscript provides cell biological mechanisms that explain how stem cells give rise to different numbers of EL neurons in different regions, including stem cell division duration and type, neural cell death, identity gene expression, and differentiation state. If the reviewer is interested in genetic or molecular mechanisms, this is an interesting point. Several prior studies using NB3-3 as a model (e.g., Tsuji et al., 2008, Birkholz et al., 2013, Baumgardt et al., 2014) have elucidated the genetic regulation of specific cell biological processes. However, these studies provided fragmentary insight with regard to serially homologous stem cell development along the body axis. A comprehensive understanding of how the NB3-3 lineage, or any other serially homologous lineage, develops was missing. This is what makes our study both novel and needed. Without an analysis that both examines every segment and assays multiple cell biological processes, we would have missed key insights: that there is a ubiquitous type of temporal cohort, and that neurons within the ubiquitous temporal cohort are selectively refined post-mitotically (See General Statements for more details).

*I disagreed with their conclusion that failure to express Eve as a mechanism for controlling EL neuron numbers when Eve serves as the marker for these neurons. Are there any other strategy to assess the fates and functions of these cells beside relying solely on Eve expression? I am not familiar with the significance of Eve expression on the functions of these neurons. Is it possible to perform clonal analyses of NB3-3 mutant for Eve and see if these neurons adopt different functionalities/identities?*

We agree that if Eve were only a marker, our logic would be circular. The Eve homolog, Evx1/2 is crucial for vertebrate interneuron cell fate (Moran-Rivard et al., 2001). Eve is essential for motor neuron morphology in *Drosophila* (Fujioka et al., 2003). Eve is critical in Even-skipped for both the morphology and function of Even-skipped interneurons (Marshall et al., 2022). Hence, ELs cannot fully differentiate or incorporate into circuits without Eve. Thus, we use the failure to express Eve as a mechanism for controlling EL number. Furthermore, our prior study (Wang et al., 2022) showed that NB3-3 Notch OFF neurons in A1 that fail to express Eve have small soma and “stick-like” neurite projections that are typical of undifferentiated neurons. We will be sure to add this context to the revised manuscript.

*If NB3-3 in the SEZ continually generate GMCs based on the interpretation of clonal analyses and depicted in Fig. 2A, why is the percent of clones that are 1:0 virtually at or near 100% from division 6-11 shown in 2G?*

Admittedly, the ts-MARCM heat-shock-based lineage tracing experiments are inherently messy. This is part of the reason why we included the G-TRACE lineage tracing experiments in Figure 3. In Figure 3E, one can see that the number of Notch ON/A neurons in SEZ3 is equal to the number of ELs in that segment (Figure 1E). This is a second independent method that supports the assertion that in SEZ, NB3-3 stem cells continually generate GMCs. Given this independent observation, it leads us to believe that this question is most likely explained by technical issues inherent in ts-MARCM. These issues include but are not limited to: cell-type specific accessibility/success of heat-shock induced recombination; variably effective RNAi; and idiosyncrasies of the *EL-GAL4* line used to detect recombination events. If the question is why the data is only reported for division 6-11, the answer is that the ts-MARCM dataset, which included SEZ clones only used later heat-shock time points (line from the paper “for the SEZ-containing dataset, inductions started at NB3-3’s 5th division”). Along with this revision plan, we will include Supplemental Table 1, which was inadvertently omitted from the previous submission due to our error. This table shows all of the clonal data. We will include a section in the discussion to describe limitations in ts-MARCM.

*The authors also indicate that NB3-3 in the abdomen directly generate Notch OFF/B cells that assume EL neuronal identity. In this scenario, shouldn't the percent of 1:0 clones be 100% in later divisions in Fig. 2G? Based on the number of clones in abdomen shown in Fig. 2E, I cannot seem to understand how the authors come to the percent of 1:0 clones shown in Fig. 2G*

We agree that one might expect the 12th division to be 100% 1:0 clones in the abdomen. Unfortunately, we didn’t sample that late in our dataset, and even when we sampled the inferred

© 2026. Published by The Company of Biologists under the terms of the Creative Commons Attribution License (https://creativecommons.org/licenses/by/4.0/).

11th division, we had a small sample size (Figure 2E). Other studies suggest that NB3-3 in the abdomen directly generates Notch OFF/B neurons (Baumgardt et al., 2014), which served as our starting point. We will revise the text to make this clearer. As you can see from Figure 3E, there is only one NB3-3 Notch ON/ A neuron produced in each abdominal segment in comparison to the number of NB3-3 Notch OFF/B/EL neurons (Figure 1E). According to two independent assessments, Figure 3 and Baumgardt et al., 2014, the data support the conclusion that NB3-3 in the abdomen directly generates Notch OFF/B cells that assume EL identity for all but one of their divisions. Again, we believe technical issues make the ts-MARCM dataset messy. We will include a section in the discussion to describe limitations in ts-MARCM.

*There are many potentially interesting questions related to this study that can significantly broaden the impact of this study. For example, are other NB lineages that also generate distinct temporal cohorts of EL neurons display similar proliferation patterns (type 1 division in SEZ, early termination of cell division in thoracic segments and type 0 division in abdomen)?*

NB3-3 is the only lineage that makes ELs; Many lineages switch proliferation fates along the body axis. Previous studies have described how this switch in division patterns produces the wedge-shaped CNS: Cobeta et al., 2017. In the revision, we will be sure to clarify both points.

*Why does NB3-3 in the thoracic segment become quiescence so much sooner than SEZ and abdominal segments?*

NB3-3 in the thorax enters quiescence due to Hox genes and temporal transcription factors (Tsuji et al., 2008). In the revision, we will be sure to clarify this point.

*The authors' observations suggest that NB3-3 in SEZ and abdomen generate a similar number of EL neurons despite the difference in their division patterns (type 1 vs type 0). Are the mechanisms that promote EL neuron generate in NB3-3 in SEZ and abdomen the same? Anything else is known beside Notch OFF?*

We agree this is an interesting point. Previous work has detailed NB3-3 division patterns, showing Type 1 divisions in the thorax, and Type 1 to Type 0 switch in the abdomen (Baumgardt et al., 2014). However, the proliferation pattern of NB3-3 in the SEZ had not been addressed until our study. Figures 2 and 3 suggest the following (1) SEZ proliferates for the duration of embryonic neurogenesis; (2) It produces a GMC on each division; (3) the GMC divides to produce one EL Notch OFF neuron and one Notch ON neuron. In our revision, we will manipulate the Notch pathway using two mutants, *sanpodo*, which produces two Notch OFF cells, and *numb*, which produces two Notch ON cells (Skeath et al., 1998), to specifically test how ELs in the SEZ are regulated by Notch signaling. The other difference we know of between the SEZ, and abdomen is Hox gene expression. In Figure S2, we show that a subset of ELs in the SEZ express the anterior Hox genes, Sex combs reduced (Scr). The role of Hox genes in this lineage is an interesting question, as addressed in the discussion. This is an important future direction that merits in- depth study and is beyond the scope of what of this study is trying to accomplish.

*Minor commentsThe authors' writing style is highly unusual especially in the result section. There is an overwhelming large amount of background information in the result section but very thin description on their observations. The background information portion also includes previously published observations. Since the nature of this study is not hypothesis-driven, it is very confusing to read in many places and difficult to distinguish their original observations from previously published results and making. One easily achievable improvement is to insert relevant figure numbers into the text more often.*

Thank you for this comment. It is invaluable. In the revision, we will expand the background into a more comprehensive introduction and present the results more clearly. We will certainly insert relevant figure numbers. In responding to the reviewer’s comments above, we can see where our writing lacked clarity and will improve these areas. Thank you again.

*Reviewer #1 (Significance (Required)):*

*The study by Vasudevan et al intends to address how serially homologous neural progenitors generate different numbers and types of neurons depending on their location along the body axis. Investigation of full repertoire of neurogenesis for these progenitors necessitates a precise ability to track the fates of both progenitors and their neuronal progeny making it extremely difficult in vertebrate paradigm. The authors used NB3-3 in the developing fly embryo as a model to*

© 2026. Published by The Company of Biologists under the terms of the Creative Commons Attribution License (https://creativecommons.org/licenses/by/4.0/). 

*investigate the full extent of the flexibility in neurogenesis from a single type of serially homologous stem cell. Previous work showed NB3-3 generates neurons including lateral interneurons that can be positively labeled by Even-skipped, but detailed characterization of the NB3-3 lineage mainly focused on 3 segments during embryogenesis. The authors defined the number of EL neurons in all segments of the central nervous system in early larvae after the completion of circuit formation and carried out clonal analyses to determine the proliferation pattern of NB3-3. They described the failure to express Eve in Notch OFF/B neurons as a new mechanism for controlling the number of EL neurons and PCD limits EL neurons in terminal segments.*

Because this text is the same as the summary, please see our response to that section.

*Reviewer #3 (Evidence, reproducibility and clarity (Required)):*

*In this manuscript, Vasudevan et al provide a detailed characterisation of the different numbers and temporal birthdates of Even-skipped Lateral (EL) neurons produced at in different segments from the same neuroblast, NB3-3. The work highlights the differences in EL neuronal generation across segments is achieved through a combination of different division patterns, failure to upregulate EL marker Eve and segment-specific program cell death. For neurons born within the same window and segment, the authors describe additional heterogeneity in their circuit formation. The work underscores the large diversity that the same neuroblast can generate across segments.*

Thank you!

*Major comments:*

*- Based on the ts-MARCM 1:0 clones representing 100% of the SEZ clones at any given inferred cell division, the authors conclude "NB3-3 neuroblasts generate proliferative daughter GMCs in the SEZ and thorax on most divisions". Figure 2G does not have any data for SEZ before inferred division 5, whereas there is data in other regions. The authors also state "In the SEZ and abdomen, ELs were labelled regardless of induction time." In reference to Fig 2F, which seems inaccurate given there are no SEZ clones before inferred division 5. There is no comment on this fact, which is surprising give their focus on temporal cohorts. The authors should explain this discrepancy, if known, or modify their statements to reflect the data.*

Thank you for raising this point. The reason is because we produced two ts-MARCM datasets. One had SEZ clones, the other did not. The dataset with SEZ clones used heat shock protocols only for later time points, because those were most informative. The text from the paper is “We combined a published ts-MARCM (Wang et al., 2022) dataset with a new one (Table S1). The differences between the datasets are (1) CNSs were imaged either at low resolution for all regions (SEZ to terminus) or higher resolution for nerve cords (thorax to terminus); (2) for the SEZ-containing dataset, inductions started at NB3-3’s 5th division. The combined data includes ~12 different heat shock protocols, 80 CNS, and 234 clones (Table S2)”. In response to this comment, however, we will further clarify this point. In addition, we are submitting Supplemental table 1, which contains all the clonal data, as you can see experiments a-h lack SEZ data and experiments i-k contain SEZ data.

*- The temporal cohort (early-born vs late-born) identity is exclusively examined based on markers. Given the absence of SEZ clones from early NB3-3 divisions, a time course showing that the SEZ generate early-born Els or some other complementary method would be desirable.*

Thank you for raising this point. We show early-born versus late-born identity using markers in Figure 5. We conducted the time-course experiment as suggested and can confirm that there are early-born ELs in the SEZ at stage 13. We will include a new Supplemental Figure that includes a time course of EL number at stages 11, 13, 15, and 17 for segments SEZ3 to Te2 in the revision. See figure below.

© 2026. Published by The Company of Biologists under the terms of the Creative Commons Attribution License (https://creativecommons.org/licenses/by/4.0/).

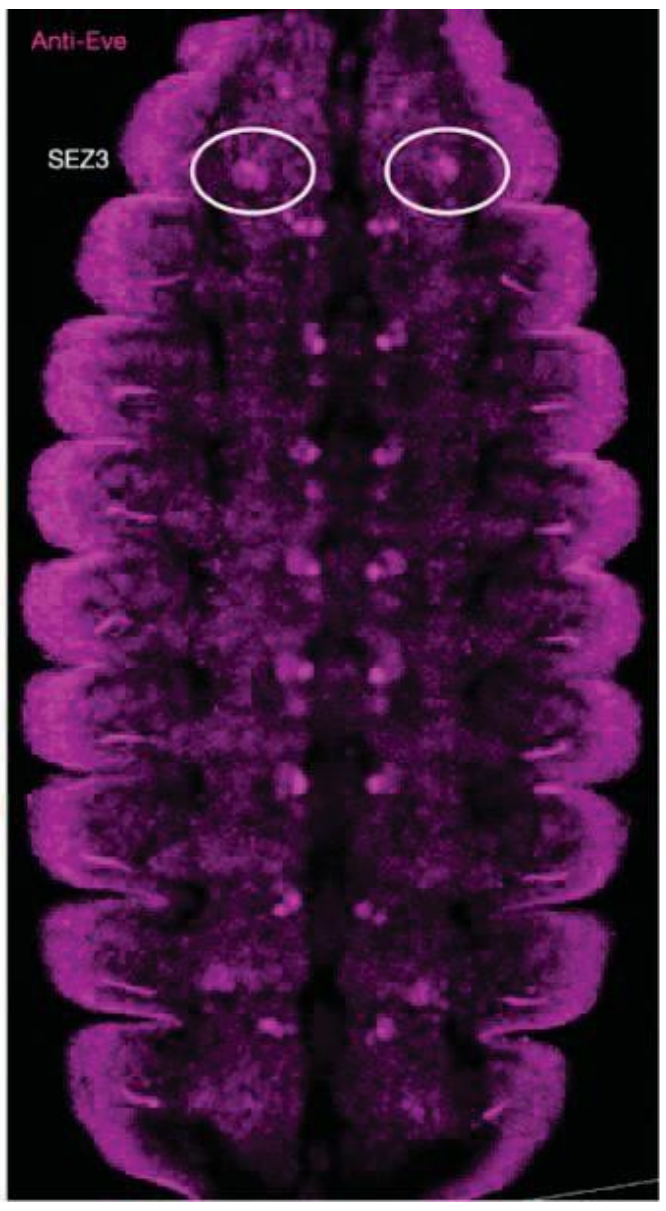


**Figure: Many EL neurons in the SEZ are present at the mid-embryonic neurogenesis stage.** The image shows a stage 13 embryo stained with anti-Eve antibody. The 5 early-born ELs are present per hemisegment in the SEZ at stage 13. Projection image of ~15 microns of a confocal stack showing ELs in SEZ3 (Circles). Anterior to the top, dorsal view.

*- The authors repeatedly refer to their work as showing how a stem cell type can have "flexibility". Flexibility would imply that NB3-3 from one segment could adopt a different behaviour (different division pattern, or cell death or connectivity) if it were placed in a different segment. This is not what is being shown. In my opinion, "heterogeneity" of the same neuroblast across different segments would be more appropriate.*

Thank you for this comment. We will change the wording to heterogeneity in the revision.

*Minor comments:*
*- Figure 2A depicts a combination of known data and conclusions from their own (mainly SEZ). The authors might consider editing the figure to highlight what is new. A possibility would be for figure A to be a diagram of the experimental design and their summary division pattern to be shown after the new data instead of being panel A.*

Thank you for this suggestion. We will make the suggested change.

*- The authors state that they combined published ts-MARCM with their new one, which differed in a number ways that they list, but they don't specify which limitations are associated with the published vs new dataset. Could the authors please clarify?*

We now include Supplemental Table 1, which shows the complete combined datasets. In the first dataset, experiments a-h, the CNS was imaged at high resolution, but in a smaller region. The limitation is that the SEZ is missing. In the second dataset, i-k, inductions started at NB3-3's 5th division. The limitation is that we fail to sample early time points. This was a strategic decision. There were two possible scenarios: (1) in the SEZ, NB3-3 divided early, made GMCs, but both daughters expressed Eve. (2) in the SEZ, NB3-3 divided for the entirety of the embryonic neurogenesis, making GMCs, with only the Notch OFF daughters expressing Eve—our data support (2). Only late heat shocks were needed to distinguish between these possibilities. As these experiments are labor-intensive, we focused our efforts on the later time points. We will make this clearer in our revised text.

*- The title refers exclusively to "temporal cohorts", which in the manuscript are defined quite narrowly and do not seem to apply to all segments.*

Thank you! This, in our opinion, is a central, not a minor point to raise, because the impact of this study involves temporal cohort biology. We outlined the essential concepts in Part 1 "general statements" section of this revision plan. We did not mean to use "temporal cohort" in a limited sense, and we can see how the writing of our results section led to this comment. We will revise to make this clear.

*- Several cited references are missing from the Reference list at the end. Could the authors please double check this? (e.g. Matsushita, 1997; Sweeney et al., 2018)*

Thank you, we will remedy this!

© 2026. Published by The Company of Biologists under the terms of the Creative Commons Attribution License (https://creativecommons.org/licenses/by/4.0/).

*- Legend for figure 2 is a bit confusing, there is a "(A)" within the legend for (D), which indicates that segments A1-A7 are shown (this seems inaccurate, as it only goes to A6).*

Thank you, we will remedy this!

*Reviewer #3 (Significance (Required)):*

*This study provides a comprehensive analysis of different cell biological scenarios for a neuroblast to generate distinct progeny across repeating axial units. The strength is the detailed and systematic approach across segments and possible scenarios: different division patterns, cell death, molecular marker expression. While it focuses on one specific neuroblast of the ventral nerve cord of Drosophila, the authors have done extensive work to place their findings and interpretation in the context of other cell types and across model organisms both in the introduction and discussion. This makes the work of interest for developmental biologists in general, neurodevelopment research in particular and those interested in circuit assembly, beyond their specialised community. This point of view comes from someone working in vertebrate CNS development.*

Thank you!

*Reviewer #4 (Evidence, reproducibility and clarity (Required)):*

*Summary*
*This manuscript addresses the question of how the number of neurons produced by each progenitor in the nervous system is determined. To address this question the authors use the Drosophila embryo model. They focus on a single type of neural stem cell (neuroblast), with homologues in each hemisegment along the anterior-posterior axis.*
*Using a combination of clonal labelling, antibody stainings, and blockade of programmed cell death, they provide a detailed description of segment-specific differences in the proliferation patterns of these neuroblasts, as well as in the fate and survival of their neuronal progeny.*
*Furthermore, by employing trans-synaptic labelling, they demonstrate that neurons derived from the same progenitor type receive distinct patterns of synaptic input depending on their segmental origin, in part due to their temporal window origin.*
*Overall this work shows that different mechanisms contribute to the final number and identity of the neuronal progeny arising from a single progenitor, even within homologous progenitors along the anterior posterior body axis.*

Thank you!

*Major Comments*
*I would suggest adding line numbers to the text for future submissions, this massively helps providing comments.*

Thank you for this comment. We will definitely add line numbers to the revised manuscript. We also thank you for providing comments despite this oversight on our part. We appreciate your time, and did not mean to make extra work.

*The authors propose that all neuroblasts produce the same type of temporal cohort (early born) and that, by changing the pattern of cell division, different temporal cohorts can be added. The way this this presented in the abstract sounds like an obvious thing, what would be the alternative scenario/s?*

Thank you for raising the point that the abstract should be updated. We have included a revised abstract. The things that are obvious are: (1) changing a neuroblast’s division pattern will change the number of neurons produced, and (2) if you have late-born neurons, the stem cell must at some point, have made early-born neurons. However, within those bounds is an extremely large parameter space. Each stem cell can choose to divide or not, and it can also choose to produce a proliferative daughter or not. The stem cell must navigate these choices at every division. The field had two models for what a stem cell might do – a “random production” model and a “unitary production” model. Our data support a third “multimodal production” model, which could not have been predicted based on prior literature or data.

We had raised these points in the discussion as follows–“Under a null model, the durations and types

© 2026. Published by The Company of Biologists under the terms of the Creative Commons Attribution License (https://creativecommons.org/licenses/by/4.0/).

of proliferation would vary stochastically across segments, resulting in a continuous and unstructured distribution of neuron numbers (Llorca et al., 2019). In a unitary production model, based on the vertebrate neocortex, there is a fixed neurogenic output of ~8–9 neurons per progenitor (Gao et al., 2014). However, our data support a third model, a multimodal production model. In a multimodal model, serially homologous neuroblasts generate different numbers of neurons depending on the segment."
We will now update the text to address this concern.

*Here it's the late born neurons that lack in thoracic segments because of early NB quiescence, but it cannot be excluded that different neuroblast types adopt a different strategy.*

True. Neural development is complex. Other lineages could easily employ alternative strategies. Our study presents a new conceptual framework that should inspire future research.

*I found the ts-MARCM results confusing for 2 reasons:*
*1- It's not clear to me why there are so many single cell clones in div 3 and 4 in abdominal segments. This is not compatible with the division model depicted for abdominal segments, unless GMCs are produced in those division window and the MARCM hits the GMC, as also mentioned in the legend for G. This aspect is important because, either the previous model by Baumgardt et al. - please correct cit. currently Gunnar et al. 2026 - is wrong, or something strange happens in this experiment, or the relative temporal order is incorrect.*

Thank you for raising this point. Having multiple single-cell (i.e., 1:0) clones in divisions 3 and 4 is not precisely what would be predicted by the model in Figure 2C. In part because heat-shock-based recombination methods in fly are stochastic and inherently "messy", we also conducted a second set of lineage tracing experiments, as shown in Figure 3, using G- TRACE. Figure 3E shows one Notch ON/A neuron in each abdominal segment, suggesting there is only one GMC present during lineage progression. But Figure 3E's result does not localize the GMC to any particular division. One possibility is that the GMC is generated once, but randomly throughout lineage progression. This possibility is consistent with the idea that the relative temporal order is incorrect and suggests that Baumgardt is erroneous. However, the Baumgardt data are strong, so we do not favor this idea. A second possibility, which we favor, is that something strange happened in this experiment. Here is how we envision the strange occurrence: heterogeneity in the EL driver. Ts-MARCM's recombination timing dictates the upper limit for the number of cells within a clone. However, recombination is detected by GAL4. So, if the GAL4 driver for some reason detects fewer cells than one expects, then one would see unusually small clones as is the case in question. To detect Ts-MARCM recombination in Figure 2, we used the *EL-GAL4* driver. The *EL-GAL4* driver is an enhancer fragment, ~400KB, meaning that it does not capture the full regulatory context of the *eve* locus. In our experience (e.g., Manning et al., 2012), drivers using small enhancers tend to give highly-specific, but somewhat variable expression, and this is the case for *EL-GAL4* in our experience. We will update the discussion to discuss the ts-MARCM dataset and its limitations. And, we will correct the citation to Baumgardt et al., 2014, not Gunnar. Thank you!

*2- In segments other than abdomen, it is quite rare to hit proper clones, it appears that only GMCs are hit by recombination, with very few exceptions. Could the author please provide an explanation for this or at least mention this aspect?*

This is true. We cannot explain it. It could have something to do with the RNAi cassettes that are used in ts-MARCM, because in the original paper they mention that RNAi can be differently regulated in GMCs versus neuroblasts (Yu et al., 2009). We will mention it in the revised discussion about ts-MARCM limitations.

*It is also unclear whether in F the graph includes all types of clones (including 1:0 clones). This is important, because the timing of division for NBs and GMCs is different, and inclusion of 1:0 might lead to a wrong estimate of the NB proliferation window (longer than it actually is because GMCs divide for longer). This is particularly important for the SEZ, where most clones in normalised division 10 and 11 are with ratio 1:0, thus compatible with both terminal division as well as GMC division.*

The graph in F does include all types of clones. We provide Supplemental Table 1, which shows the full dataset. Unfortunately, we do not have enough data to analyze only NB clones. We agree that the estimate of the NB proliferation window is coarse using this analysis method and could overrepresent the division time by one cell division. We will mention this in the discussion and

© 2026. Published by The Company of Biologists under the terms of the Creative Commons Attribution License (https://creativecommons.org/licenses/by/4.0/).

make sure that our results text is free from any overreaching claims about the precision of these measurements.

*To obtain an estimate of the timing of division, the authors normalise clone size to the size of the bigger clone in the abdomen. What happened to those samples where no abdominal clones were hit? Were they simply excluded from the analysis?*

From the analysis in Figure 2, we excluded the clones that were SEZ, thorax, or terminus only. They were rare. They are shown in Supplemental Table 1, which will now be added in our revision plan.

*It is proposed that in the thorax late temporal cohort neurons are not produced, yet the ts-MARCM experiment detects some 1:0 clones. What is the fate of these cells? Are they all derived from GMC division and therefore decoupled from the temporal identity window? Or is this a re-activation of division?*

Figure 2F shows at the inferred 11th NB3-3 division, 100% of thoracic clones are of the 1:0 type. This is an n=1 observation (Supplemental Table 1, row f-Jan20-2). When we look at the morphology of this thoracic EL, we can see that it is a fully differentiated neuron that crosses the midline and ascends to the CNS, which is similar to EL morphologies in A1, so we don't think it's a whole new cell type. We have no way of determining whether this neuron was derived from a GMC division. It is also possible that this is an infrequent event or a technical anomaly. To address the question of reactivation of the thoracic NB3-3 division, we plan to include a Supplemental Figure of EL number over developmental time (stages 11, 13, 15, 17) for segments SEZ3 to Te2. This is the same data that we mentioned to Reviewer 3. This will reveal the extent to which the thorax produces late-born ELs.

*"in A1, a majority of segments had one Notch OFF/B neuron that failed to label with Eve" does "the majority" in this sentence mean that there were cases where all B neurons were labelled with Eve? If yes, where would this stochasticity come from?*

Yes, "the majority" in this sentence means that there were cases where all B neurons were labeled by Eve. In Figure 3F, for segment A1, that number is four. In contrast, there are 6 cases where B neurons failed to label with Eve. We can only speculate about the origin of the stochasticity. It could be biological (e.g., low level of Eve expression) or technical (e.g., poor antibody penetration). We plan to mention this in the discussion.

*Additionally, there is no evidence that it's the first born NotchOFF neuron in A1 that does not express Eve. The authors should clarify where this speculation comes from.*

The evidence that the first-born Notch OFF neuron in A1 does not express Eve comes from our ts-MARCM data: "So far, our ts-MARCM analyses grouped segments into regions (Figure 2A-C), however, EL number varies on a segment-by-segment basis (Figure 1). Therefore, we looked for segment-by-segment differences in ts-MARCM data (Table S1). The only detectable difference was between A1 and the other abdominal segments: When both A1 and another abdominal segment were labeled in a single CNS, a majority had smaller A1 clones. These data suggest that the production of ELs by NB3-3 neuroblasts lags in A1 compared to A2-A7." We will add a representation of these data to the ts-MARCM figure. As we stated above, we will add a Supplemental Figure of EL number over developmental time (stages 11, 13, 15, 17) for segments SEZ3 to Te2, which could strengthen this point.

*When discussing trends shared with other phyla:*
*A- "In the mammalian spinal cord, more neurons are present in regions that control limbs (Francius et al., 2013). Analogously, EL numbers do not smoothly taper from anterior to posterior; instead, the largest number of ELs is found in two non-adjacent regions, SEZ and the abdomen." It's unclear what is the link between the figure in the mammalian spinal cord and the Drosophila embryo. The embryo doesn't even have limbs and the number of neurons measured here refer only to a single lineage, while there could be (and in fact there are) lineage-to-lineage differences that could depict a different scenario.*

Thank you for this comment. We will rewrite this sentence, "in the mammalian spinal cord, more neurons are present in regions that control limbs (Francius et al., 2013)" to more accurately reflect the data in the Francius paper, and make the parallel more explicit. We will say "the size of columns of V3, V1, V2a, V2b, and V0v neurons differ at brachial compared to lumbar levels in the developing spinal cord." This removes the confusion about limbs and somewhat mitigates the

© 2026. Published by The Company of Biologists under the terms of the Creative Commons Attribution License (https://creativecommons.org/licenses/by/4.0/).

concern about lineage-to-lineage differences, at least from the perspective of the spinal cord.

*B- The parallelism between V1 mouse neurons and EL Drosophila neurons is also unclear to me. The similarity in fold change across segments could be a pure coincidence and, from what I understand, the two cell types are not functionally linked.*

Thank you for this comment. We believe this is the sentence in question (sorry about no line numbers). "(3) In the mouse spinal cord, ~10-fold differences in molecular subtypes for V1 neurons (Sweeney et al., 2018). In *Drosophila*, NB3-3 neuroblasts show differences in EL number, depending on region, with similar fold changes, suggesting this trait is shared across phyla." The emphasis was intended to be on the fold-changes, not cell types.
Coincidence or not, it is parallel. We will update the sentence to say "(3) In the mouse spinal cord, ~10-fold differences in molecular subtypes for V1 neurons (Sweeney et al., 2018). Although V1 neurons are not direct homologs of EL neurons, the number also varies ~10- fold depending on the region. One possibility is that this trait is shared across phyla." And, we will remove the final part of the paragraph, which distracts from the point "Thus, for this study and future research, NB3-3 development now offers a uniquely tractable, detailed, and comprehensive model for studying how stem cells flexibly produce neurons."

*Minor comments:*
*I found the manuscript somewhat difficult to follow, even though I am familiar with both the model and the topic. For non-specialist readers, I expect it will be even more challenging. The presentation of the results often feels fragmented, at times resembling a sequence of brief statements rather than a continuous narrative. I would encourage the authors to provide more synthesis and interpretation, for example by summarising key findings, rather than listing in detail the number of neurons labelled in each segment for every experiment. This would make the results more accessible and easier to digest.*

Thank you for this comment. We will provide more synthesis and interpretation in results by summarizing key findings.

*From the way the MS is written it's not clear from the beginning that the work focuses exclusively on embryonic-born neurons. Since in Drosophila neuronal stem cells undergo two rounds of neurogenesis, one in the embryo and one in the larva, this omission could lead to confusion.*

Thank you for this comment. We will mention this in the abstract, introduction and discussion.

*In the abstract, what would be the other temporal cohorts generated in specific regions? (ref to: "In specific regions, NB3-3 neuroblasts produce additional types of temporal cohorts, including but not limited to the late-born EL temporal cohort.")*

In this manuscript, we use lineage tracing to identify four types of temporal cohorts– early-born Notch ON, early-born Notch OFF, late-born Notch ON, and late-born Notch OFF. This is now reflected in the revised abstract. ELs are early-born Notch OFF and/or late-born Notch OFF.

*This sentence in the introduction is inaccurate: "The Drosophila CNS is organized into an anterior hindbrain-like subesophageal zone (SEZ) and a posterior spinal cord-like nerve cord". The anterior hindbrain-like portion of the CNS is in fact the supraesophageal ganglion (or cerebrum), while the SEZ is a posterior-like region.*

Thank you. We will change this sentence to: "The *Drosophila* CNS is organized into a hindbrain-like subesophageal zone (SEZ) and a spinal cord-like nerve cord".

*Fig 1E: the encoding of the significance is not immediately clear. In the legend the 4 stars could also be arranged in the same way for clarity.*

Thank you. We will change it for clarity.

*Fig 2E legend: it is mentioned that B corresponds to a 1:4 clone, however the MARCM example is shown for C and it's a 1:5.*

Thank you. We will fix this.

*The occurrence of "undifferentiated" neurons in Th segments is in less than 10% of the clones, I wonder if this a stochastic or deterministic event and to what extent small cell bodies could just be the consequence of local differences in tissue architecture.*

© 2026. Published by The Company of Biologists under the terms of the Creative Commons Attribution License (https://creativecommons.org/licenses/by/4.0/).

Because we are using a stochastic technique, it is difficult for us to determine whether the occurrence of neurons with small somas is a stochastic or deterministic event. Several papers suggest neurons with small axons are found across insect species (Pearson and Fourtner, 1975; Burrows, 1996). Neurons with a small soma and short axons/ axonless are found in the *Drosophila* embryonic abdominal nerve cord (Lacin et al., 2009). In our unpublished work from the *Drosophila* nerve cord at a first instar larval stage, we found small somas with short axons in segment A1 (see Figure 4.6 below). This leads us to believe it is not a consequence of local tissue architecture.

**NOTE: We have removed unpublished data that had been provided for the referees in confidence.**

*Fig 2I: it's unclear what the purple means (I suppose it might be Eve expression) and why in J there should be one purple cell not labelled by the ts-MARCM when this is not present in H and I.*

Purple is Eve. We will add labels for stains used in H and I, and remove the extra purple cell from the illustration in J.

*"When synapses do occur, they are numerically similar from segment to segment". It's unclear where the evidence for this statement comes from, please clarify or remove the sentence.*

We calibrated our trans-Tango data against available connectomic data using segment A1 as a reference. We learned that the trans-tango method only identifies strongly (>15 synapses) connected neurons.

“First, we calibrated trans-Tango for use in larval *Drosophila*, focusing on segment A1, where connectome data are available (Wang et al., 2022). In the connectome, of the five early-born ELs in A1, three are strongly connected to CHOs (>15 synapses), two are weakly connected (<15 synapses), and late-born ELs are unconnected (0 synapses). In trans- Tango, in A1, ~2 ELs are labeled using IAV-GAL4 (Figure 6E). Similarly, in the connectome of the six late-born ELs in A1, three are strongly connected to proprioceptors, three are weakly connected, and the early-born ELs are unconnected. In trans-Tango, in A1, ~2 ELs are labeled using 0165-GAL4 (Figure 6F). These data suggest that the trans-Tango method identifies ELs strongly synaptically (>15 synapses) connected to somatosensory neurons.”

We will modify this sentence to say “when synapses do occur they are of similar strengths from segment to segment”

*"In SEZ2, NB3-3 divides 10 times (Figure 2F)". Figure 2F does not support this statement and Figure 7 shows 12 divisions. Possibly SEZ2 and 3 have been inverted in this statement, please clarify.*

Thank you for pointing this out. We will correct it!

***Referees cross-commenting***

*I agree with most of the comments/suggestions provided by the other two reviewers. In particular:*

*I agree with reviewer #1's comment about failure to express Eve being a mechanism for controlling neurons number, as this is a circular argument.*

We address this earlier and direct you to that text. Briefly, Eve is not just a marker, but a key differentiation gene for ELs.

*I agree with reviewer #2's concern about the use of the word "flexibility"; "heterogeneity" would be a more appropriate term, as I would associate the word "flexibility" to the ability of a single neuroblast in a single segment to produce neurons with different fates under, for example, unusual growth conditions. Here no genetic/epigenetic manipulations were performed to address flexibility and the observed (stereotypical) differences result from axial patterning.*

We will change this, thank you.

*As a note, Reviewer #1 asks about other temporal cohorts of EL neurons produced by other lineages, but these neurons are specifically generated from NB3-3.*

Thank you for adding this clarification.

*To generalise the observations reported in this study, the authors would need to focus on other*

© 2026. Published by The Company of Biologists under the terms of the Creative Commons Attribution License (https://creativecommons.org/licenses/by/4.0/).

*molecularly defined temporal cohorts or, more generally, on other lineages, which, however, are likely to adopt different combinations of mecahnisms to tune progeny number across segments.*

We agree that further studies are needed to assess the generalizability of our findings.

*Reviewer #4 (Significance (Required)):*

*In Drosophila melanogaster, the relationship between neural progenitors and their neuronal progeny has been studied in great detail. This work has provided a comprehensive description of the number of progenitors present in each embryonic segment, their molecular identities, the number of neurons they produce, and the temporal transcriptional cascades that couple progenitor temporal identity to neuronal fate.*
*This work adds to the existing knowledge a detailed characterisation of intersegmental differences in the pattern of proliferation of a single type of neuronal progenitor as well as in post-divisional fate depending on anterior-posterior position in the body axis (i.e. programmed cell death and Notch signalling activation). This is a first step towards understanding the cellular and molecular mechanisms underlying such differences, but it's not disclosing them.*

We have disclosed the cellular mechanisms– stem cell division duration and type, neural cell death, identity gene expression, and differentiation state –unless something else is envisaged by this comment. The molecular mechanisms are beyond the scope of this paper.

*That homologous neuroblasts can generate variable numbers of progeny neurons depending on their segmental position has been established previously. What this manuscript adds is the demonstration that these differences arise through a combination of altered division patterns and differential programmed cell death, thereby revealing a more complex and less predictable scenario than could have been anticipated from existing knowledge in other contexts. The advance provided by this study is therefore incremental, refining rather than overturning our understanding of how segmental diversity in neuroblast lineages is achieved.*

The key conceptual advances provided by this study are described in the General Statements section above. We don't overturn, but we advance the field.

*By touching on the general question of how progenitors generate diversity, this work could be of broad interest to developmental neuroscientists beyond the fly field. However, the way it is currently written does not make it very accessible to non-specialists.*

Thank you for this comment. We will endeavor to make it more accessible in the revised manuscript. Reviewer 3, an expert in vertebrate neurobiology, agreed that our work was of broad interest.

*My expertise: Drosophila neurodevelopment, nerve cord, cell types specification*

3. Description of the revisions that have already been incorporated in the transferred manuscript

Please insert a point-by-point reply describing the revisions that were already carried out and included in the transferred manuscript. If no revisions have been carried out yet, please leave this section empty.

With this Revision Plan, we submit a revised abstract, and a supplemental table 1. We plan to address every point raised by the reviewers.

4. Description of analyses that authors prefer not to carry out

*Please include a point-by-point response explaining why some of the requested data or additional analyses might not be necessary or cannot be provided within the scope of a revision. This can be due to time or resource limitations or in case of disagreement about the necessity of such additional data given the scope of the study. Please leave empty if not applicable.*

© 2026. Published by The Company of Biologists under the terms of the Creative Commons Attribution License (https://creativecommons.org/licenses/by/4.0/).

**Original submission**

First decision letter

MS ID#: dev.205389

MS Title: How serially homologous neuroblasts produce different temporal cohorts along the Drosophila larval body axis

Authors: Deeptha Vasudevan; Yi-wen Wang; Hannah Carr; Elise Paniel; Sean Corcoran; Chris Wreden; Elaine Kushkowski; Conor Lee-Smith; Ellie Heckscher
Article Type: Review Commons Transfer

Dear Dr Heckscher,

Thank you for sending your manuscript to Development through Review Commons.

I have now had the chance to review your documents, and we would like to invite you to revise your manuscript according to your revision plan. It will be important to clarify the ts-MARCM clonal data and to address the concern raised regarding Eve expression as a mechanism for controlling neuron numbers. Please also take note of the comments on the manuscript's accessibility. Once we receive the revised version, together with your point-by-point response to the referees, we will seek further input from the Review Commons referees. If you have any questions about this process, please do get in touch.

**First revision**

Author response to reviewers' comments

Reviewer Responses

Manuscript ID#: dev.205389R1

Review Commons ID: RC-2025-03130

Manuscript title: **How serially homologous neuroblasts produce different temporal cohorts along the *Drosophila* larval body axis**

Corresponding author(s): Ellie S. Heckscher

We thank all three reviewers for their feedback on the paper. Reviewers stated that the paper was of broad interest to developmental biologists and neurobiologists. Our revised manuscript has the following changes:

- We revised text across all sections of the manuscript and added schematics to all figure panels.
- We created two new figures, Figures 6 and 7 from data panels from older versions of Figures 2 and 3 to make our findings clearer.
- We added three new pieces of data to address reviewer comments: Notch pathway manipulation in Figure 3, time course analysis of EL number in stages 13, 15, and 17 in Figure 5, and analysis of clonal data between segments A1 and A2-A7 in Supplemental Figure 3.

In addition to these changes, we want to ensure that our two key conceptual contributions are clear. We clarify below along with our revised manuscript:

The first advance is that serially homologous neuroblasts follow a multimodal production model: In principle, stem cells can divide any number of times, from once to throughout the entire

© 2026. Published by The Company of Biologists under the terms of the Creative Commons Attribution License (https://creativecommons.org/licenses/by/4.0/). 

lifetime of the animal. And, on each division, a stem cell can generate either a proliferative daughter cell or a post-mitotic neuron. Together, therefore, there is a vast potential number of neurons any given stem cell could produce. From the literature on the vertebrate neocortex, we had the following models: (1) "random production" model, in which any number of neurons could be made by a stem cell; or (2) "unitary production" model, in which the same number of neurons (~eight) is produced by a stem cell regardless of context. Our data revealed an entirely new "multi-modal production" model, which could not have been predicted by prior literature. In the context of serially homologous neuroblasts arrayed along the *Drosophila* larval body axis, sets of five to seven neurons are produced in increments of one, two, or four. These increments correspond to units called temporal cohorts. Temporal cohorts are lineage fragments, or small set of neurons that share synaptic partners, making them lineage-based units of circuit assembly. Thus, in a multimodal production model, serially homologous stem cells produce different numbers of temporal cohorts depending on location. Our data advance the field by showing that stem cells produce circuit-relevant sets of neurons by adding or omitting temporal cohorts from a region, to meet regional needs.

Key to understanding the second advance is that there are multiple types of temporal cohorts: early-born Notch OFF, early-born Notch ON, late-born Notch OFF, and late-born Notch ON. One temporal cohort type, the early-born Notch OFF, is found in every segment, which we term the "fundamental" temporal cohort. The other temporal cohort types can be produced in various combinations depending on the stem cell division pattern and segmental location. In a result that could not have been predicted, we found that the fundamental temporal cohorts are refined both in terms of the number of neurons and their connectivity, depending on body region. In contrast, when other temporal cohort types are produced, they are not refined to the same degree.

The impact of this work is to advance how we think about stem cell-based circuit assembly.

*Reviewer #1 (Evidence, reproducibility and clarity (Required)):*

*Summary: The study by Vasudevan et al intends to address how serially homologous neural progenitors generate different numbers and types of neurons depending on their location along the body axis.*
*Investigation of full repertoire of neurogenesis for these progenitors necessitates a precise ability to track the fates of both progenitors and their neuronal progeny making it extremely difficult in vertebrate paradigm. The authors used NB3-3 in the developing fly embryo as a model to investigate the full extent of the flexibility in neurogenesis from a single type of serially homologous stem cell. Previous work showed NB3-3 generates neurons including lateral interneurons that can be positively labeled by Even-skipped, but detailed characterization of the NB3-3 lineage mainly focused on 3 segments during embryogenesis. The authors defined the number of EL neurons in all segments of the central nervous system in early larvae after the completion of circuit formation and carried out clonal analyses to determine the proliferation pattern of NB3-3. They described the failure to express Eve in Notch OFF/B neurons as a new mechanism for controlling the number of EL neurons and PCD limits EL neurons in terminal segments.*
Thank you! In addition to the contributions highlighted by the reviewer, we also showed that all segments have ELs with early-born molecular identities, but only a subset have ELs with late-born identities (Figure 4). And we showed that early-born temporal cohorts can be mapped into different circuits depending on the axial region (Figure 8).

*Major comments: The authors performed careful analyses of the NB3-3 lineage using EL neurons. My main concerns are limited applicability of their findings and lack of mechanisms as how NB3-3 generate various numbers of EL neurons. Their findings are exclusively relevant to the NB3-3 lineage despite their effort in highlighting that other NB lineages also generate temporal cohorts of EL neurons.*
Thank you for raising these points. First, to clarify, as Reviewer 4 also mentioned, NB3-3 is the only lineage to produce EL neurons. We clarified this in the introduction and results of the revised text.

We agree that our findings might not apply beyond the NB3-3 lineage. However, as this is the first study of its kind, it is impossible to know a priori to what extent the concepts surfaced here are generalizable. In our opinion, this speaks to the novelty and impact of the study. A contribution of our study is to motivate a need for future studies. We clarify this in our updated manuscript in the Discussion section.

Our manuscript provides cell biological mechanisms that explain how stem cells give rise to different numbers of EL neurons in different regions, including stem cell division duration and type, Notch pathway manipulations, neural cell death, identity gene expression, and differentiation state.

© 2026. Published by The Company of Biologists under the terms of the Creative Commons Attribution License (https://creativecommons.org/licenses/by/4.0/).

If the reviewer is interested in genetic or molecular mechanisms, this is an interesting point. Several prior studies using NB3-3 as a model (e.g., Tsuji et al., 2008, Birkholz et al., 2013, Baumgardt et al., 2014) have elucidated the genetic regulation of specific cell biological processes. We mention these in the updated Introduction and Discussion sections. However, these studies provided fragmentary insight with regard to serially homologous stem cell development along the body axis. A comprehensive understanding of how the NB3-3 lineage, or any other serially homologous lineage, develops was missing. This is what makes our study both novel and needed. Without an analysis that both examines every segment and assays multiple cell biological processes, we would have missed key insights: that there is a fundamental type of temporal cohort, and that neurons within the fundamental temporal cohort are selectively refined post-mitotically (See General Statements for more details).

*I disagreed with their conclusion that failure to express Eve as a mechanism for controlling EL neuron numbers when Eve serves as the marker for these neurons. Are there any other strategy to assess the fates and functions of these cells beside relying solely on Eve expression? I am not familiar with the significance of Eve expression on the functions of these neurons. Is it possible to perform clonal analyses of NB3-3 mutant for Eve and see if these neurons adopt different functionalities/identities?*
We agree that if Eve were only a marker, our logic would be circular. The Eve homolog, Evx1/2 is crucial for vertebrate interneuron cell fate (Moran-Rivard et al., 2001). Eve is critical for both the morphology and function of Even-skipped interneurons (Marshall et al., 2022). Hence, ELs cannot fully differentiate or incorporate into circuits without Eve. Thus, we use the failure to express Eve as a mechanism for controlling EL number. Furthermore, our prior study (Wang et al., 2022) showed that NB3-3 Notch OFF neurons in A1 that fail to express Eve have small soma and “stick-like” neurite projections that are typical of undifferentiated neurons. We added this context to the introduction and results of the revised manuscript.

*If NB3-3 in the SEZ continually generate GMCs based on the interpretation of clonal analyses and depicted in Fig. 2A, why is the percent of clones that are 1:0 virtually at or near 100% from division 6-11 shown in 2G?*
Admittedly, the ts-MARCM heat-shock-based lineage tracing experiments are inherently messy. This is part of the reason why we included the G-TRACE lineage tracing experiments in Figure 3. In Figure 3C, one can see that the number of Notch ON/A neurons in SEZ3 is equal to the number of ELs in that segment (Figure 1E). This is a second independent method that supports the assertion that in SEZ, NB3-3 stem cells continually generate GMCs. Given this independent observation, it leads us to believe that this question is most likely explained by technical issues inherent in ts-MARCM. These issues include but are not limited to: cell-type specific accessibility/success of heat-shock induced recombination; variably effective RNAi; and idiosyncrasies of the *EL-GAL4* line used to detect recombination events. If the question is why the data is only reported for division 6-11, the answer is that the ts-MARCM dataset, which included SEZ clones only used later heat-shock time points. We addressed this by adding a schematic of the two different datasets analyzed in Figure 2. In addition, Table S1 and S2 show all of the clonal data. We also included limitations in ts-MARCM in the Methods section to address this.

*The authors also indicate that NB3-3 in the abdomen directly generate Notch OFF/B cells that assume EL neuronal identity. In this scenario, shouldn't the percent of 1:0 clones be 100% in later divisions in Fig. 2G? Based on the number of clones in abdomen shown in Fig. 2E, I cannot seem to understand how the authors come to the percent of 1:0 clones shown in Fig. 2G*
We agree that one might expect the 12th division to be 100% 1:0 clones in the abdomen. Unfortunately, we didn’t sample that late in our dataset, and even when we sampled the inferred 11th division, we had a small sample size (Figure 2F in the current version). Other studies suggest that NB3-3 in the abdomen directly generates Notch OFF/B neurons (Baumgardt et al., 2014), which served as our starting point. We revised the text to make this clearer. As you can see from Figure 3C, there is only one NB3-3 Notch ON/ A neuron produced in each abdominal segment in comparison to the number of NB3-3 Notch OFF/B/EL neurons (Figure 1E). According to two independent assessments, Figure 3 and Baumgardt et al., 2014, the data support the conclusion that NB3-3 in the abdomen directly generates Notch OFF/B cells that assume EL identity for all but one of their divisions. Again, we believe technical issues make the ts-MARCM dataset messy. These limitations in ts-MARCM data are described in Methods section.

© 2026. Published by The Company of Biologists under the terms of the Creative Commons Attribution License (https://creativecommons.org/licenses/by/4.0/).

*There are many potentially interesting questions related to this study that can significantly broaden the impact of this study. For example, are other NB lineages that also generate distinct temporal cohorts of EL neurons display similar proliferation patterns (type 1 division in SEZ, early termination of cell division in thoracic segments and type 0 division in abdomen)?*
NB3-3 is the only lineage that makes ELs; Many lineages switch proliferation fates along the body axis. Previous studies have described how this switch in division patterns produces the wedge-shaped CNS: Cobeta et al., 2017. We clarify both points in the introduction and Discussion section of the revised manuscript respectively.

*Why does NB3-3 in the thoracic segment become quiescence so much sooner than SEZ and abdominal segments?*
NB3-3 in the thorax enters quiescence due to Hox genes and temporal transcription factors (Tsuji et al., 2008). In the revision, we clarify this point in the Introduction and Discussion.

*The authors' observations suggest that NB3-3 in SEZ and abdomen generate a similar number of EL neurons despite the difference in their division patterns (type 1 vs type 0). Are the mechanisms that promote EL neuron generate in NB3-3 in SEZ and abdomen the same? Anything else is known beside Notch OFF?*
We agree this is an interesting point. Previous work has detailed NB3-3 division patterns, showing Type 1 divisions in the thorax, and Type 1 to Type 0 switch in the abdomen (Baumgardt et al., 2014). However, the proliferation pattern of NB3-3 in the SEZ had not been addressed until our study. Figures 1E, 2 and 3 suggest the following: (1) SEZ proliferates for the duration of embryonic neurogenesis; (2) It produces a GMC on each division; (3) the GMC divides to produce one EL Notch OFF neuron and one Notch ON neuron. In our revised Figure 3, we included data from manipulating the Notch pathway using two mutants, *sanpodo*, which produces Notch OFF cells, and *numb*, which produces Notch ON cells (Skeath et al., 1998). We found that ELs in the SEZ are eliminated in *numb* mutants and that EL number in the SEZ is doubled in *sanpodo* mutants (Figure 3E and G). The other difference we know of between the SEZ, and abdomen is Hox gene expression. In Figure S2, we show that a subset of ELs in the SEZ express the anterior Hox genes, Sex combs reduced (Scr). The role of Hox genes in this lineage is an interesting question, as addressed in the discussion. This is an important future direction that merits in-depth study and is beyond the scope of what of this study is trying to accomplish.

*Minor commentsThe authors' writing style is highly unusual especially in the result section. There is an overwhelming large amount of background information in the result section but very thin description on their observations. The background information portion also includes previously published observations. Since the nature of this study is not hypothesis-driven, it is very confusing to read in many places and difficult to distinguish their original observations from previously published results and making. One easily achievable improvement is to insert relevant figure numbers into the text more often.*
Thank you for this comment. It is invaluable. In the revised manuscript, we have provided a more comprehensive introduction and presented the results more clearly. (We hope!). We revised all sections in this version of the manuscript, and created new figures, Figure 6 and 7 from data panels from old versions of Fig 2 and 3 to make our results clearer. We added three new pieces of data to address reviewer comments: Notch pathway manipulation in Figure 3, time course analysis of EL number in stages 13, 15, and 17 in Figure 5, and analysis of clonal data between segments A1 and A2-A7 in Supplemental Figure 3. We also inserted relevant figure numbers across sections. In responding to the reviewer's comments above, we are hopeful that the revised version has more clarity. Thank you again.

*Reviewer #1 (Significance (Required)):*

*The study by Vasudevan et al intends to address how serially homologous neural progenitors generate different numbers and types of neurons depending on their location along the body axis. Investigation of full repertoire of neurogenesis for these progenitors necessitates a precise ability to track the fates of both progenitors and their neuronal progeny making it extremely difficult in vertebrate paradigm. The authors used NB3-3 in the developing fly embryo as a model to investigate the full extent of the flexibility in neurogenesis from a single type of serially homologous stem cell. Previous work showed NB3-3 generates neurons including lateral interneurons that can be positively labeled by Even-skipped, but detailed characterization of the NB3-3 lineage mainly focused on 3*

© 2026. Published by The Company of Biologists under the terms of the Creative Commons Attribution License (https://creativecommons.org/licenses/by/4.0/).

*segments during embryogenesis. The authors defined the number of EL neurons in all segments of the central nervous system in early larvae after the completion of circuit formation and carried out clonal analyses to determine the proliferation pattern of NB3-3. They described the failure to express Eve in Notch OFF/B neurons as a new mechanism for controlling the number of EL neurons and PCD limits EL neurons in terminal segments.*
Because this text is the same as the summary, please see our response to that section.

*Reviewer #3 (Evidence, reproducibility and clarity (Required)):*

*In this manuscript, Vasudevan et al provide a detailed characterisation of the different numbers and temporal birthdates of Even-skipped Lateral (EL) neurons produced at in different segments from the same neuroblast, NB3-3. The work highlights the differences in EL neuronal generation across segments is achieved through a combination of different division patterns, failure to upregulate EL marker Eve and segment-specific program cell death. For neurons born within the same window and segment, the authors describe additional heterogeneity in their circuit formation. The work underscores the large diversity that the same neuroblast can generate across segments.*
Thank you!

*Major comments:*
*- Based on the ts-MARCM 1:0 clones representing 100% of the SEZ clones at any given inferred cell division, the authors conclude "NB3-3 neuroblasts generate proliferative daughter GMCs in the SEZ and thorax on most divisions". Figure 2G does not have any data for SEZ before inferred division 5, whereas there is data in other regions. The authors also state "In the SEZ and abdomen, ELs were labelled regardless of induction time." In reference to Fig 2F, which seems inaccurate given there are no SEZ clones before inferred division 5. There is no comment on this fact, which is surprising give their focus on temporal cohorts. The authors should explain this discrepancy, if known, or modify their statements to reflect the data.*
Thank you for raising this point. The reason is because we produced two ts-MARCM datasets. One had SEZ clones, the other did not. The dataset with SEZ clones used heat shock protocols only for later time points, because those were most informative. In response to this comment, we have clarified this point by adding schematics to the tsMARCM figure (Figure 2A). From the text “We analyzed a published ts-MARCM dataset (Wang et al., 2022) and a new dataset (Figure 2A, Table S1). In the Wang dataset, the entirety of neurogenesis was analyzed, but the SEZ was not imaged (Figure 2A). In our new dataset, we imaged the entire CNS but assayed only later neurogenesis (Figure 2A)”. The combined dataset includes 11 different heat shock protocols, 80 CNS, and 234 clones (Figure 2A, Table S2). In addition, Supplemental table 1 contains all the clonal data, as you can see experiments a-h lack SEZ data and experiments i-k contain SEZ data.

*- The temporal cohort (early-born vs late-born) identity is exclusively examined based on markers. Given the absence of SEZ clones from early NB3-3 divisions, a time course showing that the SEZ generate early-born Els or some other complementary method would be desirable.*
Thank you for raising this point. We show early-born versus late-born identity using markers in Figure 4. We conducted the time-course experiment as suggested and can confirm that there are early-born ELs in the SEZ at stage 13. We included additional images and quantifications for EL number at stages 13, 15, and 17 for segments SEZ2 to Te2 in the revision (Figure 5A-F).

*- The authors repeatedly refer to their work as showing how a stem cell type can have "flexibility". Flexibility would imply that NB3-3 from one segment could adopt a different behaviour (different division pattern, or cell death or connectivity) if it were placed in a different segment. This is not what is being shown. In my opinion, "heterogeneity" of the same neuroblast across different segments would be more appropriate.*
Thank you for this comment. We changed the wording to heterogeneity in the revision.

*Minor comments:*
*- Figure 2A depicts a combination of known data and conclusions from their own (mainly SEZ). The authors might consider editing the figure to highlight what is new. A possibility would be for figure A to be a diagram of the experimental design and their summary division pattern to be shown after the new data instead of being panel A.*

© 2026. Published by The Company of Biologists under the terms of the Creative Commons Attribution License (https://creativecommons.org/licenses/by/4.0/). 

Thank you for this suggestion. We changed panel 2A (which is now 2B in the revised figure) to show division patterns and labeling using ts-MARCM.

*- The authors state that they combined published ts-MARCM with their new one, which differed in a number ways that they list, but they don't specify which limitations are associated with the published vs new dataset. Could the authors please clarify?*
Supplemental Table 1 shows the complete combined datasets. In the first dataset, experiments a-h, the CNS was imaged at high resolution, but in a smaller region. The limitation is that the SEZ is missing. In the second dataset, i-k, inductions started at NB3-3's 5th division. The limitation is that we fail to sample early time points. This was a strategic decision. There were two possible scenarios: (1) in the SEZ, NB3-3 divided early, made GMCs, but both daughters expressed Eve. (2) in the SEZ, NB3-3 divided for the entirety of the embryonic neurogenesis, making GMCs, with only the Notch OFF daughters expressing Eve. Our data support (2). Only late heat shocks were needed to distinguish between these possibilities. As these experiments are labor-intensive, we focused our efforts on the later time points. We clarified this in the revised Methods.

*- The title refers exclusively to "temporal cohorts", which in the manuscript are defined quite narrowly and do not seem to apply to all segments.*
Thank you! This, in our opinion, is a central, not a minor point to raise, because the impact of this study involves temporal cohort biology. We outlined the essential concepts in Part 1 “general statements” section of this revision plan. We did not mean to use “temporal cohort” in a limited sense, and we can see how the writing of our results section led to this comment. We have revised this framing to include temporal cohorts all throughout the text.

*- Several cited references are missing from the Reference list at the end. Could the authors please double check this? (e.g. Matsushita, 1997; Sweeney et al., 2018)*
Thank you, we have fixed this.

*- Legend for figure 2 is a bit confusing, there is a "(A)" within the legend for (D), which indicates that segments A1-A7 are shown (this seems inaccurate, as it only goes to A6).*
Thank you, we have revised the figure legend.

*Reviewer #3 (Significance (Required)):*

*This study provides a comprehensive analysis of different cell biological scenarios for a neuroblast to generate distinct progeny across repeating axial units. The strength is the detailed and systematic approach across segments and possible scenarios: different division patterns, cell death, molecular marker expression. While it focuses on one specific neuroblast of the ventral nerve cord of Drosophila, the authors have done extensive work to place their findings and interpretation in the context of other cell types and across model organisms both in the introduction and discussion. This makes the work of interest for developmental biologists in general, neurodevelopment research in particular and those interested in circuit assembly, beyond their specialised community. This point of view comes from someone working in vertebrate CNS development.*
Thank you!

*Reviewer #4 (Evidence, reproducibility and clarity (Required)):*

*Summary*
*This manuscript addresses the question of how the number of neurons produced by each progenitor in the nervous system is determined. To address this question the authors use the Drosophila embryo model. They focus on a single type of neural stem cell (neuroblast), with homologues in each hemisegment along the anterior-posterior axis.*
*Using a combination of clonal labelling, antibody stainings, and blockade of programmed cell death, they provide a detailed description of segment-specific differences in the proliferation patterns of these neuroblasts, as well as in the fate and survival of their neuronal progeny.*
*Furthermore, by employing trans-synaptic labelling, they demonstrate that neurons derived from the same progenitor type receive distinct patterns of synaptic input depending on their segmental origin, in part due to their temporal window origin.*

© 2026. Published by The Company of Biologists under the terms of the Creative Commons Attribution License (https://creativecommons.org/licenses/by/4.0/).

*Overall this work shows that different mechanisms contribute to the final number and identity of the neuronal progeny arising from a single progenitor, even within homologous progenitors along the anterior posterior body axis.*
Thank you!

*Major Comments*
*I would suggest adding line numbers to the text for future submissions, this massively helps providing comments.*
Thank you for this comment. We have added line numbers to the revised manuscript. We also thank you for providing comments despite this oversight on our part. We appreciate your time and did not mean to make extra work.

*The authors propose that all neuroblasts produce the same type of temporal cohort (early born) and that, by changing the pattern of cell division, different temporal cohorts can be added. The way this this presented in the abstract sounds like an obvious thing, what would be the alternative scenario/s?*
We have included a revised abstract. The things that are obvious are: (1) changing a neuroblast's division pattern will change the number of neurons produced, and (2) if you have late-born neurons, the stem cell must at some point, have made early-born neurons. However, within those bounds is an extremely large parameter space. Each stem cell can choose to divide or not, and it can also choose to produce a proliferative daughter or not. The stem cell must navigate these choices at every division. The field had two models for what a stem cell might do – a "random production" model and a "unitary production" model. Our data support a third "multimodal production" model, which could not have been predicted based on prior literature or data. We expand on this idea in the introduction and discussion section.

Our revised abstract reads "Our results show that serially homologous neuroblasts in certain regions modify proliferation patterns, adding or omitting temporal cohorts, leading to extra neurons for specialized circuits. Four types of temporal cohorts are produced, but one type, which we term a "fundamental" temporal cohort, appears in every region. Neurons from this cohort are further refined post-mitotically, enabling regional circuit specialization."

*Here it's the late born neurons that lack in thoracic segments because of early NB quiescence, but it cannot be excluded that different neuroblast types adopt a different strategy.*
True. Neural development is complex. Other lineages could easily employ alternative strategies. We hope that our study presents a new conceptual framework that inspires future research.

*I found the ts-MARCM results confusing for 2 reasons:*
*1- It's not clear to me why there are so many single cell clones in div 3 and 4 in abdominal segments. This is not compatible with the division model depicted for abdominal segments, unless GMCs are produced in those division window and the MARCM hits the GMC, as also mentioned in the legend for G. This aspect is important because, either the previous model by Baumgardt et al. - please correct cit. currently Gunnar et al. 2026 - is wrong, or something strange happens in this experiment, or the relative temporal order is incorrect.*
Thank you for raising this point. Having multiple single-cell (i.e., 1:0) clones in divisions 3 and 4 is not precisely what would be predicted by the schematic in Figure 2B. In part because heat-shock-based recombination methods in fly are stochastic and inherently "messy", we also conducted a second set of lineage tracing experiments, as shown in Figure 3, using G-TRACE. Figure 3C shows one Notch ON/A neuron in each abdominal segment, suggesting there is only one GMC present during lineage progression. But Figure 3C's result does not localize the GMC to any particular division. One possibility is that the GMC is generated once, but randomly throughout lineage progression. This possibility is consistent with the idea that the relative temporal order is incorrect and suggests that Baumgardt is erroneous. However, the Baumgardt data are strong, so we do not favor this idea. A second possibility, which we favor, is that something strange happened in this experiment. Here is how we envision the strange occurrence: heterogeneity in the EL driver. Ts-MARCM's recombination timing dictates the upper limit for the number of cells within a clone. However, recombination is detected by GAL4. So, if the GAL4 driver for some reason detects fewer cells than one expects, then one would see unusually small clones as is the case in question. To detect Ts-MARCM recombination in Figure 2, we used the *EL-GAL4* driver. The *EL-GAL4* driver is an enhancer fragment, ~400KB, meaning that it does not capture the full regulatory context of the *eve* locus. In our experience (e.g.,

© 2026. Published by The Company of Biologists under the terms of the Creative Commons Attribution License (https://creativecommons.org/licenses/by/4.0/).

Manning et al., 2012), drivers using small enhancers tend to give highly-specific, but somewhat variable expression, and this is the case for *EL-GAL4* in our experience. We have updated the Methods section to discuss the ts-MARCM dataset and its limitations. And, we have corrected the citation to Baumgardt et al., 2014, not Gunnar. Thank you!

*2- In segments other than abdomen, it is quite rare to hit proper clones, it appears that only GMCs are hit by recombination, with very few exceptions. Could the author please provide an explanation for this or at least mention this aspect?*
This is true. We cannot explain it. It could have something to do with the RNAi cassettes that are used in ts-MARCM, because in the original paper they mention that RNAi can be differently regulated in GMCs versus neuroblasts (Yu et al., 2009). We have added this to the ts-MARCM limitations in the Methods section.

*It is also unclear whether in F the graph includes all types of clones (including 1:0 clones). This is important, because the timing of division for NBs and GMCs is different, and inclusion of 1:0 might lead to a wrong estimate of the NB proliferation window (longer than it actually is because GMCs divide for longer). This is particularly important for the SEZ, where most clones in normalised division 10 and 11 are with ratio 1:0, thus compatible with both terminal division as well as GMC division.*
The graph in Figure 2E (previously 2F) does include all types of clones. Supplemental Table 1 shows the full dataset. Unfortunately, we do not have enough data to analyze only NB clones. We agree that the estimate of the NB proliferation window is coarse using this analysis method and could overrepresent the division time by one cell division. We clarify this in the Methods and have updated our results text to be free from any overreaching claims about the precision of these measurements.

*To obtain an estimate of the timing of division, the authors normalise clone size to the size of the bigger clone in the abdomen. What happened to those samples where no abdominal clones were hit? Were they simply excluded from the analysis?*
From the analysis in Figure 2, we excluded the clones that were SEZ, thorax, or terminus only. They were rare. They are shown in Supplemental Table 1. We now mention this in the Methods.

*It is proposed that in the thorax late temporal cohort neurons are not produced, yet the ts-MARCM experiment detects some 1:0 clones. What is the fate of these cells? Are they all derived from GMC division and therefore decoupled from the temporal identity window? Or is this a re-activation of division?*
Figure 2E shows at the inferred 11th NB3-3 division, 100% of thoracic clones are of the 1:0 type. This is an n=1 observation (Supplemental Table 1, row f-Jan20-2). When we look at the morphology of this thoracic EL, we can see that it is a fully differentiated neuron that crosses the midline and ascends to the CNS, which is similar to EL morphologies in A1. So, we don't think it's a whole new cell type. We have no way of determining whether this neuron was derived from a GMC division. It is also possible that this is an infrequent event or a technical anomaly. To address the question of reactivation of the thoracic NB3-3 division, we included figures and quantification of EL number over developmental time (stages 13, 15, 17) for segments SEZ2 to Te2, now in Figure 5(A-F). This is the same data that we mentioned to Reviewer 3. This reveals that thoracic segments do not often produce late-born ELs, because EL number stays similar from stage 13 to stage 15. So, if reactivation does occur, it is rare.

*"in A1, a majority of segments had one Notch OFF/B neuron that failed to label with Eve" does "the majority" in this sentence mean that there were cases where all B neurons were labelled with Eve? If yes, where would this stochasticity come from?*
Yes, "the majority" in this sentence means that there were cases where all B neurons were labeled by Eve. Figure 6 in the revised manuscript shows the number of Notch OFF/B neurons that fail to express Eve. In Figure 6C and D, for segment A1, we see that in 4 cases (hemisegments), there are no Notch OFF/B neurons that fail to express Eve. In contrast, there are 6 cases where B neurons failed to label with Eve. We can only speculate about the origin of the stochasticity. It could be biological (e.g., low level of Eve expression) or technical (e.g., poor antibody penetration).

*Additionally, there is no evidence that it's the first born NotchOFF neuron in A1 that does not express Eve. The authors should clarify where this speculation comes from.*
The model in Figure 6A where the first-born Notch OFF/B fails to express Eve is from Wang et al., 2022. The evidence that there is a Notch OFF/ B Eve(-) neuron in A1 comes from our G-TRACE data in

© 2026. Published by The Company of Biologists under the terms of the Creative Commons Attribution License (https://creativecommons.org/licenses/by/4.0/). 

revised Figure 6. The evidence that it is the first-born Notch OFF/B neuron is from Supplemental Figure 3, using tsMARCM. When we looked for segment-by-segment differences in ts-MARCM data (Table S1), the only detectable difference was between A1 and the other abdominal segments. When both A1 and another abdominal segment were labeled in a single CNS, a majority had smaller A1 clones. These data suggest that the production of ELs by NB3-3 neuroblasts lags in A1 compared to A2-A7. This is now described in the results, under the section titled "In A1 and the terminus, a subset of early-born Notch OFF/B neurons do not express Eve".

*When discussing trends shared with other phyla:*
*A- "In the mammalian spinal cord, more neurons are present in regions that control limbs (Francius et al., 2013). Analogously, EL numbers do not smoothly taper from anterior to posterior; instead, the largest number of ELs is found in two non-adjacent regions, SEZ and the abdomen." It's unclear what is the link between the figure in the mammalian spinal cord and the Drosophila embryo. The embryo doesn't even have limbs and the number of neurons measured here refer only to a single lineage, while there could be (and in fact there are) lineage-to-lineage differences that could depict a different scenario.*
Thank you for this comment. We have now updated out discussion as follows: "To put our results into a broader phylogenetic context, we note that the NB3-3 lineage exhibits trends observed in other phyla. (1) In the spinal cord, p0 progenitors generate Evx(+) V0v neurons (EL analogs) and other neurons (Satou et al., 2012). In *Drosophila*, NB3-3 neuroblasts make Eve(+) ELs and other neurons (Figure 9). (2) Across species, the highest neuron numbers are found in the anterior CNS (Cobeta et al., 2017). The distribution of neuron numbers in the NB3-3 lineage follows this trend (Figure 9). (3) In the mouse spinal cord, there can be ~10-fold differences in molecular neuronal subtypes (Sweeney et al., 2018). Similar fold changes are found in various neuron types in the NB3-3 lineage (Figure 9). (4) In the mouse spinal cord, the largest numbers of a particular subtype are found in non-adjacent regions (Francius et al., 2013). In *Drosophila*, the largest number of ELs are found in non-adjacent SEZ and abdomen (Figure 9)."

*B- The parallelism between V1 mouse neurons and EL Drosophila neurons is also unclear to me. The similarity in fold change across segments could be a pure coincidence and, from what I understand, the two cell types are not functionally linked.*
Thank you for this comment. We agree that the parallelism is unclear, and we hope that the updated section (in the answer above) clarifies these points.

*Minor comments:*
*I found the manuscript somewhat difficult to follow, even though I am familiar with both the model and the topic. For non-specialist readers, I expect it will be even more challenging. The presentation of the results often feels fragmented, at times resembling a sequence of brief statements rather than a continuous narrative. I would encourage the authors to provide more synthesis and interpretation, for example by summarising key findings, rather than listing in detail the number of neurons labelled in each segment for every experiment. This would make the results more accessible and easier to digest.*
Thank you for this comment. We have provided more synthesis and interpretation in results by summarizing key findings in the revised manuscript.

*From the way the MS is written it's not clear from the beginning that the work focuses exclusively on embryonic-born neurons. Since in Drosophila neuronal stem cells undergo two rounds of neurogenesis, one in the embryo and one in the larva, this omission could lead to confusion.*
Thank you for this comment. We have clarified this in the introduction.

*In the abstract, what would be the other temporal cohorts generated in specific regions? (ref to: "In specific regions, NB3-3 neuroblasts produce additional types of temporal cohorts, including but not limited to the late-born EL temporal cohort.")*
In this manuscript, we use lineage tracing to identify four types of temporal cohorts– early-born Notch ON, early-born Notch OFF, late-born Notch ON, and late-born Notch OFF. This is now reflected in the revised abstract. ELs are early-born Notch OFF and/or late-born Notch OFF.

*This sentence in the introduction is inaccurate: "The Drosophila CNS is*

© 2026. Published by The Company of Biologists under the terms of the Creative Commons Attribution License (https://creativecommons.org/licenses/by/4.0/).

*organized into an anterior hindbrain-like subesophageal zone (SEZ) and a posterior spinal cord-like nerve cord". The anterior hindbrain-like portion of the CNS is in fact the supraesophageal ganglion (or cerebrum), while the SEZ is a posterior-like region.*
Thank you. We have changed this sentence to: “The *Drosophila* body and CNS are organized into four regions: the subesophageal zone (SEZ), thorax (Th), abdomen (A), and terminus (Te), and each region comprises multiple segments (Figure 1A)."

*Fig 1E: the encoding of the significance is not immediately clear. In the legend the 4 stars could also be arranged in the same way for clarity.*
Thank you. We have changed the asterisks to the same orientation in the figure and legends for clarity.

*Fig 2E legend: it is mentioned that B corresponds to a 1:4 clone, however the MARCM example is shown for C and it's a 1:5.*
Thank you. We have fixed this in Figure 2.

*The occurrence of "undifferentiated" neurons in Th segments is in less than 10% of the clones, I wonder if this a stochastic or deterministic event and to what extent small cell bodies could just be the consequence of local differences in tissue architecture.*
Because we are using a stochastic technique, it is difficult for us to determine whether the occurrence of neurons with small somas is a stochastic or deterministic event. Several papers suggest neurons with small axons are found across insect species (Pearson and Fourtner, 1975; Burrows, 1996). Neurons with a small soma and short axons/ axonless are found in the *Drosophila* embryonic abdominal nerve cord (Lacin et al., 2009). We updated this context in our results section.

In our unpublished work from the *Drosophila* nerve cord at a first instar larval stage, we found small somas with short axons in segment A1 (see Figure 4.6 below). This leads us to believe it is not a consequence of local tissue architecture.

**NOTE: We have removed unpublished data that had been provided for the referees in confidence.**

*Fig 2I: it's unclear what the purple means (I suppose it might be Eve expression) and why in J there should be one purple cell not labelled by the ts-MARCM when this is not present in H and I.*
This is now part of revised Fig.7A-C. Purple is Eve. We have added labels for stains used, and we removed the extra purple cell from the illustration.

*"When synapses do occur, they are numerically similar from segment to segment". It's unclear where the evidence for this statement comes from, please clarify or remove the sentence.*
We removed this sentence. We calibrated our trans-Tango data against available connectomic data using segment A1 as a reference. We learned that the trans-tango method only identifies strongly (>15 synapses) connected neurons. “First, we calibrated Trans-Tango for use in ELs, focusing on segment A1, where connectome data are available (Wang et al., 2022). For connectivity to CHOs, of the five early-born ELs in A1, three are strongly connected to CHOs (>15 synapses), two are weakly connected (<15 synapses), and late-born ELs are unconnected (0 synapses). In trans-Tango, in A1, ~2 ELs are labeled using IAV-GAL4 (Figure 8E). For connectivity to DBDs, of the six late-born ELs in A1, three are strongly connected to proprioceptors, three are weakly connected, and the early-born ELs are unconnected. In trans-Tango, in A1, ~2 ELs are labeled using 0165-GAL4 (Figure 8F). These data suggest that the Trans-Tango method identifies ELs that are strongly synaptically connected to CHO or DBD neurons.” We clarified the conclusions in the revised version of this section.

*"In SEZ2, NB3-3 divides 10 times (Figure 2F)". Figure 2F does not support this statement and Figure 7 shows 12 divisions. Possibly SEZ2 and 3 have been inverted in this statement, please clarify.*
Thank you for pointing this out. We now have an updated discussion section.

***Referees cross-commenting***

*I agree with most of the comments/suggestions provided by the other two reviewers.*
*In particular:*

© 2026. Published by The Company of Biologists under the terms of the Creative Commons Attribution License (https://creativecommons.org/licenses/by/4.0/).

*I agree with reviewer #1's comment about failure to express Eve being a mechanism for controlling neurons number, as this is a circular argument.*
We address this earlier and direct you to that text. Briefly, Eve is not just a marker, but a key differentiation gene for ELs. We have also clarified this in the introduction and results.

*I agree with reviewer #2's concern about the use of the word "flexibility"; "heterogeneity" would be a more appropriate term, as I would associate the word "flexibility" to the ability of a single neuroblast in a single segment to produce neurons with different fates under, for example, unusual growth conditions. Here no genetic/epigenetic manipulations were performed to address flexibility and the observed (stereotypical) differences result from axial patterning.*
We have changed this, thank you.

*As a note, Reviewer #1 asks about other temporal cohorts of EL neurons produced by other lineages, but these neurons are specifically generated from NB3-3.*
Thank you for adding this clarification.

*To generalise the observations reported in this study, the authors would need to focus on other molecularly defined temporal cohorts or, more generally, on other lineages, which, however, are likely to adopt different combinations of mecahnisms to tune progeny number across segments.*
We agree that further studies are needed to assess the generalizability of our findings.

*Reviewer #4 (Significance (Required)):*

*In Drosophila melanogaster, the relationship between neural progenitors and their neuronal progeny has been studied in great detail. This work has provided a comprehensive description of the number of progenitors present in each embryonic segment, their molecular identities, the number of neurons they produce, and the temporal transcriptional cascades that couple progenitor temporal identity to neuronal fate.*
*This work adds to the existing knowledge a detailed characterisation of intersegmental differences in the pattern of proliferation of a single type of neuronal progenitor as well as in post-divisional fate depending on anterior-posterior position in the body axis (i.e. programmed cell death and Notch signalling activation). This is a first step towards understanding the cellular and molecular mechanisms underlying such differences, but it's not disclosing them.*
We have disclosed the cellular mechanisms–stem cell division duration and type, Notch pathway manipulation, neural cell death, identity gene expression, and differentiation state. The molecular mechanisms are beyond the scope of this paper.

*That homologous neuroblasts can generate variable numbers of progeny neurons depending on their segmental position has been established previously. What this manuscript adds is the demonstration that these differences arise through a combination of altered division patterns and differential programmed cell death, thereby revealing a more complex and less predictable scenario than could have been anticipated from existing knowledge in other contexts. The advance provided by this study is therefore incremental, refining rather than overturning our understanding of how segmental diversity in neuroblast lineages is achieved.*
The key conceptual advances provided by this study are described in the General Statements section above. We don’t overturn, but we advance the field.

*By touching on the general question of how progenitors generate diversity, this work could be of broad interest to developmental neuroscientists beyond the fly field. However, the way it is currently written does not make it very accessible to non-specialists.*
Thank you for this comment. We have endeavored to make the revised manuscript more accessible by revising all sections, and by using panels from Fig 2 and 3 to make Fig 6 and 7. Reviewer 3, an expert in vertebrate neurobiology, agreed that our work was of broad interest.

*My expertise: Drosophila neurodevelopment, nerve cord, cell types specification*
Thank you again for your expert comments.

© 2026. Published by The Company of Biologists under the terms of the Creative Commons Attribution License (https://creativecommons.org/licenses/by/4.0/).

Second decision letter

MS ID#: dev.205389R1

MS Title: How serially homologous neuroblasts produce different temporal cohorts along the Drosophila larval body axis

Authors: Deeptha Vasudevan; Yi-wen Wang; Hannah Carr; Elise Paniel; Sean Corcoran; Chris Wreden; Elaine Kushkowski; Conor Lee-Smith; Ellie Heckscher
Article Type: Review Commons Transfer

Dear Dr Heckscher,

I have now received all the referees' reports on the above manuscript, and have reached a decision. The referees' comments are appended below.

As you will see, the referees express considerable interest in your work and acknowledge the improvements made in revision. Nevertheless, two substantive concerns require attention before the manuscript can be accepted. First, the temporal cohort framework needs careful consideration. The "early/late" dichotomy is not fully supported by the data, which show within-window heterogeneity, and the conceptual conflation of temporal identity with Notch-mediated hemilineage specification requires clarification. Second, the technical limitations of the ts-MARCM approach should be communicated clearly in the main text, particularly regarding the absence of SEZ early clones and the discrepancy at Line 146. You are also asked to address the absent 11F02 expression in SEZ2 and the weak statistical support for the birth-order claim at Line 305, and to correct the minor errors and nomenclature issues flagged by Reviewer 1.

If you are able to revise the manuscript along the lines suggested, which may involve further experiments, I will be happy to receive a revised version of the manuscript. Your revised paper will be re-reviewed by one or more of the original referees, and acceptance of your manuscript will depend on your addressing satisfactorily the reviewers' major concerns. Please also note that Development will normally permit only one round of major revision. If it would be helpful, you are welcome to contact us to discuss your revision in greater detail. Please send us a point-by-point response indicating your plans for addressing the referees' comments, and we will look over this and provide further guidance.

Please attend to all of the reviewers' comments and ensure that you upload both a 'clean' version of your Word file, along with a highlighted version clearly showing where you have made changes in the revised manuscript. Please avoid using 'Tracked changes' in Word files as these are lost in PDF conversion. I should be grateful if you would also provide a point-by-point response detailing how you have dealt with the points raised by the reviewers in the 'Response to Reviewers' box. If you do not agree with any of their criticisms or suggestions please explain clearly why this is so.

Reviewer 1

In this revised version of the manuscript the authors have significantly improved clarity and the manuscript is now much easier to parse.

Most of my previous concerns have been addressed convincingly and/or the authors have explicitly stated the limits of the study.

However I still have some concerns:

I have concerns regarding the rigidity of the temporal cohort definitions proposed by the authors. First, I find it conceptually problematic to separate neurons born within the same temporal window into different cohorts based on Notch signalling status, as temporal identity and Notch-dependent hemilineage specification represent distinct and orthogonal patterning mechanisms. More

© 2026. Published by The Company of Biologists under the terms of the Creative Commons Attribution License (https://creativecommons.org/licenses/by/4.0/).

importantly, the definition of "early" and "late" cohorts appears overly coarse. Statements such as "producing neurons in multiples of 5-7" imply discrete and homogeneous temporal blocks, yet the data do not fully support this simplification. For example, in Figure 4, early-born neurons in A1 show heterogeneity in the expression of markers such as Kr and Kn, suggesting that this population could be further subdivided into distinct temporal sub-cohorts.

In this context, the absence of 11F02 expression is used as a defining feature of early-born EL neurons; however, this binary distinction may mask underlying temporal complexity within the early window itself. Altogether, the data seem more consistent with a progressive temporal specification model rather than a strict early/late dichotomy.

The authors investigate molecular similarity across segments and conclude that temporal cohorts are molecularly similar. However, they fail to detect 11F02 expression in SEZ2, where late-born EL neurons are expected. This result leaves room for molecular divergence of temporal cohorts across regions and exceptions to the rule that temporal cohorts are molecularly similar across regions.

Lines 62-63 (related to a previously raised concern)- the addition of "body" compared to the previous version does not improve, if something it makes it more confusing: SEZ refers to the CNS, while Thorax and Abdomen are body regions. My initial concern was related to the absence of the brain (hindbrain) in the description of the CNS, beyond the SEZ and VNC segments. If they want to introduce the segmental organisation of the Drosophila embryo as well, I would suggest treating body parts and CNS separately in terms of nomenclature. For the CNS I'd talk about SEZ, thoracic segments, abdominal segments and terminal segments (also in Fig. 1a).

According to Fig.2A, it looks as if in this study 4 heat shock protocols were carried on, but according to Table S1, only protocols i, j and k have SEZ clones, so I understand these were the experiments performed newly for this manuscript. I would remove one lightning symbol from 2A and add the dataset source in Table S1

Line 146 refers to Fig. 2E for SEZ clones being generated only in late heat shock protocols, however there are SEZ clones already at normalised division 5, which I believe would be considered as part of the early temporal cohort. This likely means that either the normalisation method is not very accurate, or there is such a variability in the developmental progression of embryos that the different protocols don't mean much per se. The authors should provide an explanation for this. They already mentioned in the rebuttal letter that ts-MARCM experiments are messy, but all these dubious aspects undermine the credibility of the model.

Line 305: I personally don't see a clear trend in Figure S3, I counted 13 cases of A1A2-A7. Therefore the evidence supporting the statement that Eve- NotchOFF neurons are the first born is weak.

Line 186: should be Figure 3C.

Line 264: figure number missing

Line 303-304: please check the grammar, the sentence appears truncated.

Reviewer 2

The Vasudevan et al study is the revised version of a manuscript originally submitted to Review Commons. Their work intends to address how serially homologous neural progenitors generate different numbers and types of neurons depending on their location along the body axis. The implication of their work could extend well beyond invertebrate neural development and likely relevant to vertebrate neurogenesis. The authors have done a thoughtful job addressing reviewer's comments and contextualize their findings in a broadly applicable manner. The reviewer has two minor suggestions and enthusiastically support publication of this study.

Minor suggestions

© 2026. Published by The Company of Biologists under the terms of the Creative Commons Attribution License (https://creativecommons.org/licenses/by/4.0/). 

1. line119-120, "and/or changing whether they produce a proliferative or a post-mitotic daughter (herein, division type)". I would like to suggest that the authors consider adopting the descriptive termology (proliferation mode and type 0 division) used in the Baumgardt et al., Dev Cell 2014.

2. In the result section "NB3-3 neuroblasts produce different numbers of Notch ON/A neurons across regions", the "Notch ON/A neuron" and :Notch OFF/B neuron" nomenclatures are potentially confusing and difficult for some readers to correlate the description in the text to Fig. 3H. For example, the Notch ON/A neurons in wild-type embryos actually assume the fate of Notch OFF/B neurons in sanpodo-mutant embryos. The Notch OFF/B neurons in wild-type embryos actually assume Notch ON/A neuronal fate. Will it be possible to simply describe these two categories of neurons as A vs. B?

Reviewer 3

*Advance summary and potential significance to field*

The authors provide a detailed description of the numbers of neurons that NB3-3 neuroblasts produce at different segments. They go on to characterise whether the different number of neurons is a consequence of different division pattern, expression of the marker or apoptosis.

*Comments for the author*

1 - General comments

For this work to be relevant to other beyond the specific field, the manuscript should be made more accessible both conceptually and technically. Some examples:

Conceptual: (1) what makes these neurons across segments a "class"? They express one shared marker but could the authors elaborate on any functional similarities between NB3-3-derived neurons across segments. (2) time is continuos, what defines early and late born cohorts are for example drawn in "the model that arises from these data" in fig 2G.

Technical: the ts-MARCM technique and its analysis is still quite different to understand if one is not already familiar with it.

2- Specific

This in turn raises an important major point: it is hard to understand the technical limitations and put the authors' interpretation in context. I think it would be essential to (1) briefly explain what the "heat shock time relative to inferred NB3-3 division number" is and how it's calculated in the main text. This is essential to understand a major part of the manuscript. (2) Clearly acknowledge the several instances in which the ts-MARCM system has limitations, as discussed in the exchange with reviewers. This can't just be limited to the methods sections as the reader needs to understand the limitations. The fact that the Methods section on ts-MARCM is now 5 pages to include all the experimental idiosyncrasies and limitations is very concerning. If so many clarifications are needed, I worry if the conclusions of the authors are really justified.

It is still unclear how the authors draw conclusions from SEZ if there are no early clones. I find it confusing to plot distributions of percentages of clones (Fig 2E,F) across timepoints and regions. It's impossible to observe SEZ early as they were not assayed but to anyone looking at the figure you might think they were assayed but not detected.

---

**Second revision**

Author response to reviewers' comments

Hi Dr. Briscoe,

© 2026. Published by The Company of Biologists under the terms of the Creative Commons Attribution License (https://creativecommons.org/licenses/by/4.0/).

Thank you for handling our manuscript as editor. Please find our revised manuscript attached. Briefly, the updated manuscript has revised text based on reviewer comments, and 4 revised figures: Figure 2 (updated schematics for ts-MARCM and heat shock protocols), Figure 4 (updated with quantification of late-born marker Lin29), Supplementary Figure 2 (showing additional image panels of ELs expressing Lin29), and Supplementary Figure 3 (showing an updated pie chart of differences between A1 and A2-A7 EL clones in our ts-MARCM dataset, as well as additional images and quantifications of EL number in A1 and A2 for early developmental stages 11 and 12). The manuscript was also edited to adhere to the journal's word limit.

There are two sections below:

(A) Responses to editor comments and (B) Responses to reviewer comments.

## (A) Responses to editor comments

(1) First, the temporal cohort framework needs careful consideration. The "early/late" dichotomy is not fully supported by the data, which show within-window heterogeneity, and the conceptual conflation of temporal identity with Notch-mediated hemilineage specification requires clarification.

We did not want to remove the temporal cohort frame from this version of the manuscript, as most reviewers responded much more positively to this manuscript version that included it. So, we softened the language in the results and expanded the introduction (please see highlighted sections in the revised text: lines 64-90).

(2) Second, the technical limitations of the ts-MARCM approach should be communicated clearly in the main text, particularly regarding the absence of SEZ early clones and the discrepancy at Line 146.

We added a paragraph to the results section that better describes the dataset's production, our analysis methods, and briefly mentions the caveats (lines 131-194). We have revised Figure 2A to show schematics for ts-MARCM and 2B shows the various heat shock protocols, as the older version introduced some confusion. We have also revised Figure 2F and G to better illustrate that the combination of what is known about temporal cohorts in the system, already augmented by ts-MARCM data, yields a model that is tested in the remainder of the paper.

(3) You are also asked to address the absent 11F02 expression in SEZ2

We have added the point raised by reviewer 1, "This result leaves room for molecular divergence of temporal cohorts across regions" to the text.

We assayed a second late-born marker, Lin29 (Tsuji et al., 2008), and added quantifications of Lin29+ ELs as a panel (D) to Figure 4. This helps us demonstrate the extent to which SEZ2 is differs from other regions that produce late-born neurons.

(4) You are also asked to address the … weak statistical support for the birth-order claim at Line 305.

This is regarding Supplemental Figure 3, which shows differences between A1 and A2-A7 EL counts. The idea is that there are more ELs in A2 that A1 regardless of heat-shock time, so the production of ELs in A2-A7 leads that of A1 by 1 EL at all time points. We now quantify the data shown as a pie chart—to highlight that in approximately 50% of cases, A2-A7 EL numbers are greater than in A1, in ~25% of cases they are equal, and in ~25% of cases they are fewer. We think this reflects real biology rather than a technical aspect of ts-MARCM, because in Figure 5, you can see that at stage 13 and stage 15, there are often but not always fewer ELs in A1 compared to A2. To make this point, we

© 2026. Published by The Company of Biologists under the terms of the Creative Commons Attribution License (https://creativecommons.org/licenses/by/4.0/).

have added additional graphs in Supplemental Figure 3. In this new graph, we compare the total EL numbers in A1 and A2 per embryo at the early stages of development (stages 11 and 12). We also edited our language to say “a majority” rather than most. Nonetheless, these data are supportive of the idea the reason there is one less neuron in A1 compare to A2-A7, is that the first-born neuron from NB3-3 in segment A1 is not differentiated. We also noted in our main text that this was already the model in the field (Wang, et al, 2022), so we are supporting a prior hypothesis, not generating a new one.

(5) You are also asked to address … nomenclature issues flagged by Reviewer 1.

We have addressed these. Please see below and revised text.

**(B) Responses to Reviewer comments**

Reviewer 1: In this revised version of the manuscript the authors have significantly improved clarity and the manuscript is now much easier to parse.

Thank you for your feedback!

Most of my previous concerns have been addressed convincingly and/or the authors have explicitly stated the limits of the study.

Thank you!

However I still have some concerns:
I have concerns regarding the rigidity of the temporal cohort definitions proposed by the authors. First, I find it conceptually problematic to separate neurons born within the same temporal window into different cohorts based on Notch signalling status, as temporal identity and Notch- dependent hemilineage specification represent distinct and orthogonal patterning mechanisms.

We agree that Notch signaling and temporal identity are distinct and orthogonal patterning mechanisms. However, we did not introduce this concept. It was first introduced by Mark and Doe in their 2021 eLife paper. We have now included this information in the introduction to the paper. Please see our revised introduction (lines 64-90) for our exact text changes.

More importantly, the definition of "early" and "late" cohorts appears overly coarse. Statements such as "producing neurons in multiples of 5-7" imply discrete and homogeneous temporal blocks, yet the data do not fully support this simplification.

We agree that there is nothing intuitive about having sets of neurons in 5-7 and subdividing them into early and late. However, this is where the data in the field has taken us and others in a series of several papers (Wreden et al., 2017, Current Biology, Meng et al. 2019 eLife, Meng et al. 2020 eLife, Mark and Doe, eLife, Wang et al., 2022 eLife). We have now included more of a historical overview of the origin and current use of the term temporal cohort.

For example, in Figure 4, early-born neurons in A1 show heterogeneity in the expression of markers such as Kr and Kn, suggesting that this population could be further subdivided into distinct temporal sub-cohorts.

We agree that Kr and Kn expression are not uniform. The idea behind a temporal cohort is not that neurons are identical, only that they are more similar to each other than to any neuron in another temporal cohort. Indeed, we are fairly certain from connectome work that no two neurons in *Drosophila* larvae are identical morphologically or in terms of their synaptic inputs and outputs. We have added additional context about this in line 301-308.

© 2026. Published by The Company of Biologists under the terms of the Creative Commons Attribution License (https://creativecommons.org/licenses/by/4.0/).

In this context, the absence of 11F02 expression is used as a defining feature of early-born EL neurons; however, this binary distinction may mask underlying temporal complexity within the early window itself. Altogether, the data seem more consistent with a progressive temporal specification model rather than a strict early/late dichotomy.

This is an important point. But we argue these two models— early/late dichotomy, and progressive specification— are not in conflict and not a dichotomy. There can be two windows, and within those windows, there can be progressive temporal specification. One example, from Wang 2022, is that morphologically, within the early-born EL temporal cohort, axons are long for the earliest of early-borns, getting progressively shorter. Then, within the late-born temporal cohort, the earliest of the late-borns also has a long axon with axons getting progressively shorter.

Here is how we have amended the text in the introduction:

Temporal cohorts, a framework used in this study, are lineage fragments, or sets of 5-7 neurons born within a tight time window. Temporal cohorts were first identified in the NB3-3 lineage (Wreden et al., 2017) and comprise early-born and late-born Even-skipped (Eve)-Lateral (EL) neurons. NB3-3 is the only lineage that produces ELs (Bossing et al., 1996; Gunnar et al., 2016). All EL neurons require somatosensory stimuli to fire, making them sensory processing neurons (Heckscher et al., 2015). Generally, ELs are commissural and excitatory, like their counterparts in the vertebrate spinal cord, the commissural, excitatory, EVX-expressing (Eve homolog) V0v interneurons (Heckscher et al., 2015). The initial assumption was that all ELs were similar, but this was challenged because only late-born ELs express an 11F02 enhancer fragment (Heckscher et al., 2014). Independently activating the early-born and late-born ELs triggered escape and postural control behavioral responses, respectively (Wreden et al., 2017). Early-born ELs respond to vibrational stimuli, whereas late-born ELs respond to proprioceptive stimuli (Wreden et al., 2017). Despite some morphological differences, all five early-born ELs in segment A1 receive direct synaptic input from chordotonal (vibration-sensitive) sensory neurons. All six late-born ELs in A1, despite some morphological differences, received direct synaptic input from proprioceptive sensory neurons (Wang et al., 2022). Thus, temporal cohorts are lineage fragments of similar, but non-identical neurons that nonetheless share function and connectivity. Extending the idea of temporal cohorts to lineages beyond NB3-3, other groups of morphologically similar but not identical neurons united by similar birth timing, stem cell parent, and Notch status exhibit significantly similar connectivity patterns (Mark et al., 2021). Temporal cohorts and temporal cohort-like units are found in the Drosophila nerve cord and mushroom body (Meng et al., 2019; Meng et al., 2020; Wang et al., 2022; Mark et al., 2021; Baek and Mann, 2009; Kunz et al., 2012), as well as the mouse spinal cord and neocortex (Stam et al., 2012; Gao et al., 2014). Generally, the importance of temporal cohorts lies in Drosophila embryonic neuroblasts populating larval circuits by assembling temporal cohorts into circuit motifs (Wang et al., 2022). A significant gap in temporal cohort biology is how their numbers and types vary across the body axis.

And we removed the phrase "temporal cohort" from the final paragraph of the introduction, replacing it with the phrase "sets of 5-7 neurons." We made this same change for the section header **"NB3-3 neuroblasts produce different sets of 5-7 neurons in each region"** We have updated the final paragraph in that section to read "Extrapolating from what we know about NB3-3 temporal cohorts in segment A1 and the thorax, our data introduce a model in which, depending on the region, NB3-3 neuroblasts exhibit distinct proliferation patterns to generate different sets of temporal cohorts in each region (Figure 2G). Specifically, the SEZ might be comprised of four temporal cohorts—early-born Notch OFF/B (ELs), early- born Notch ON/A, late-born Notch OFF/B (ELs), and late-born Notch ON/A (Figure 2G). The thorax would have two temporal cohorts—early-born Notch OFF/B (ELs) and early-born Notch ON/A. We already know that A1, and likely the rest of the abdomen, has early-born Notch OFF/B and late-born Notch OFF/B temporal cohorts. In the terminus, there would only be an early-born Notch OFF/B temporal cohort. In this model, NB3-3 generates the early-born Notch OFF/B temporal cohort in every segment and other types in other segments."

The authors investigate molecular similarity across segments and conclude that temporal cohorts

© 2026. Published by The Company of Biologists under the terms of the Creative Commons Attribution License (https://creativecommons.org/licenses/by/4.0/).

are molecularly similar. However, they fail to detect 11F02 expression in SEZ2, where late-born EL neurons are expected. This result leaves room for molecular divergence of temporal cohorts across regions and exceptions to the rule that temporal cohorts are molecularly similar across regions.

We amended the title of the section to read “**Temporal cohorts are molecularly similar, but not identical, across regions”.** In addition, we added Lin-29 data to Fig.4 and Supplemental Figure 2. And now the final paragraph reads “In summary, our data support the idea that EL temporal cohorts across different regions are molecularly similar, but not identical. The observation that 11F02 does not label ELs in SEZ2 leaves room for molecular divergence of temporal cohorts across regions. Furthermore, the molecular differences in the number of Kr(+) and Kn (+) early-born ELs suggest that, within a temporal cohort, neurons are heterogeneous. Indeed, although prior connectome analysis showed statistically significant connectivity among early-born ELs of A1, the morphology of each early-born EL in A1 and its connectivity is distinct (Wang et al., 2020). Motivated by the observation of heterogeneous expression, we probe for post-mitotic differences between the ELs across segments in the sections below.”

Lines 62-63 (related to a previously raised concern)- the addition of "body" compared to the previous version does not improve, if something it makes it more confusing: SEZ refers to the CNS, while Thorax and Abdomen are body regions. My initial concern was related to the absence of the brain (hindbrain) in the description of the CNS, beyond the SEZ and VNC segments. If they want to introduce the segmental organisation of the Drosophila embryo as well, I would suggest treating body parts and CNS separately in terms of nomenclature. For the CNS I'd talk about SEZ, thoracic segments, abdominal segments and terminal segments (also in Fig. 1a).

We removed the word “body” and updated Fig. 1A

According to Fig.2A, it looks as if in this study 4 heat shock protocols were carried on, but according to Table S1, only protocols i, j and k have SEZ clones, so I understand these were the experiments performed newly for this manuscript. I would remove one lightning symbol from 2A and add the dataset source in Table S1

Thank you for raising this point. We have removed the current Fig 2A and replaced it with a schematic of ts-MARCM and added Fig.2B which depicts the 11 heat shock protocols we used and reflects Table S1 as you suggested. We hope this will clear up much of the remaining confusion. Thank you again for this helpful suggestion.

Line 146 refers to Fig. 2E for SEZ clones being generated only in late heat shock protocols, however there are SEZ clones already at normalised division 5, which I believe would be considered as part of the early temporal cohort. This likely means that either the normalisation method is not very accurate, or there is such a variability in the developmental progression of embryos that the different protocols don't mean much per se. The authors should provide an explanation for this. They already mentioned in the rebuttal letter that ts-MARCM experiments are messy, but all these dubious aspects undermine the credibility of the model.

Indeed, we find SEZ clones at division five. This is to be expected by the idea that in the SEZ NB3-3 continually divides to make ELs. Two possibilities are raised by the reviewer to explain this observation: (1) that the normalization is not very accurate, (2) or that there is so much variability that the experiments are meaningless. However, there is a third reason: the heat shock was delivered at normalized division 5, which is the case in question. We can see that the way we describe our heat shock protocols led to confusion, and so the suggested changes to Figure 2A and B should help clarify this.

Line 305: I personally don't see a clear trend in Figure S3, I counted 13 cases of A1A2-A7. Therefore the evidence supporting the statement that Eve- NotchOFF neurons are the first born is weak.

Supplemental Figure 3 shows differences between A1 and A2-A7 EL counts. The idea is that there are more ELs in A2 that A1 regardless of heat-shock time, so the production of ELs in A2- A7 leads

© 2026. Published by The Company of Biologists under the terms of the Creative Commons Attribution License (https://creativecommons.org/licenses/by/4.0/). 

that of A1 by 1 EL at all time points. We have quantified the data shown in a previous version of Fig S3 in a pie chart to highlight that in approximately 50% of cases, A2-A7 EL numbers are greater than in A1, in ~25% of cases they are equal, and in ~25% of cases they are fewer. We think this reflects real biology rather than a technical aspect of ts-MARCM, because in Figure 5, you can see that at stage 13 and stage 15, there are often but not always fewer ELs in A1 compared to A2. To make this point, we have added additional graphs, like the one currently shown in Supplemental Figure 3. Here, we compared the total EL numbers in A1 and A2 per embryo at the early stages of development (stages 11 and 12). We also edited our language to say "a majority" rather than most. Nonetheless, these data are supportive of the idea the reason there is one less neuron in A1 compared to A2-A7, is that the first-born neuron from NB3-3 in segment A1 is not differentiated. We also noted in our main text that this was already the model in the field (Wang, et al, 2022), so we are supporting a prior hypothesis, not generating a new one.

Line 186: should be Figure 3C.
Line 264: figure number missing
Line 303-304: please check the grammar, the sentence appears truncated.

We have fixed these errors. Thank you for catching them.

Reviewer 2: The Vasudevan et al study is the revised version of a manuscript originally submitted to Review Commons. Their work intends to address how serially homologous neural progenitors generate different numbers and types of neurons depending on their location along the body axis. The implication of their work could extend well beyond invertebrate neural development and likely relevant to vertebrate neurogenesis. The authors have done a thoughtful job addressing reviewer's comments and contextualize their findings in a broadly applicable manner. The reviewer has two minor suggestions and enthusiastically support publication of this study.

Thank you so much!

Minor suggestions

1. line119-120, "and/or changing whether they produce a proliferative or a post-mitotic daughter (herein, division type)". I would like to suggest that the authors consider adopting the descriptive termology (proliferation mode and type 0 division) used in the Baumgardt et al., Dev Cell 2014.

We updated the text (please see line 125-126 in revised version).

2. In the result section "NB3-3 neuroblasts produce different numbers of Notch ON/A neurons across regions", the "Notch ON/A neuron" and :Notch OFF/B neuron" nomenclatures are potentially confusing and difficult for some readers to correlate the description in the text to Fig. 3H. For example, the Notch ON/A neurons in wild-type embryos actually assume the fate of Notch OFF/B neurons in sanpodo-mutant embryos. The Notch OFF/B neurons in wild-type embryos actually assume Notch ON/A neuronal fate. Will it be possible to simply describe these two categories of neurons as A vs. B?

We updated the text to read as follows (line 260-274):

> We also assayed Notch ON/A neurons in the NB3-3 lineage by manipulating the Notch pathway, using *sanpodo* and *numb* mutants. In *sanpodo* mutants, all neurons adopt a Notch OFF/B fate, whereas in *numb* mutants they adopt the Notch ON/A fate (Skeath and Doe, 1998). We stained mutant embryos with Eve to assay phenotypes as in the prior work. First, these mutants functioned as previously reported: The Notch ON/A U motor neurons from the NB7-1 lineage were missing in *sanpodo* and doubled in *numb* (Figure 3D-F). Next, we examined the NB3-3 lineage in mutants. Across segments, the Notch OFF/B ELs were nearly absent in *numb*, confirming prior reports and extending them to the SEZ, thorax, and terminus

© 2026. Published by The Company of Biologists under the terms of the Creative Commons Attribution License (https://creativecommons.org/licenses/by/4.0/).

(Skeath and Doe, 1998) (Figure 3E). In *sanpodo*, the effect on EL number varied dramatically across regions, so we counted ELs (Figure 3F-G). In *sanpodo*, the SEZ and thorax have nearly twice as many ELs as controls (Figure 3F-G), which is consistent with a model in which all daughters of a GMC division become Notch OFF/B ELs, rather than half becoming Notch ON/B and the other half becoming Notch ON/A (Figure 3H). In the abdomen and terminus, there was only a small increase in EL production (Figure 3F-G), which is consistent with a model in which there is only one GCM division in these regions (Figure 3H).

Reviewer 3: SUMMARY OF THE ADVANCE MADE IN THIS PAPER AND ITS POTENTIAL SIGNIFICANCE TO THE FIELD

The authors provide a detailed description of the numbers of neurons that NB3-3 neuroblasts produce at different segments. They go on to characterise whether the different number of neurons is a consequence of different division pattern, expression of the marker or apoptosis.

SUGGESTIONS TO AUTHORS

1 - General comments
For this work to be relevant to other beyond the specific field, the manuscript should be made more accessible both conceptually and technically. Some examples:

Conceptual: (1) what makes these neurons across segments a "class"? They express one shared marker but could the authors elaborate on any functional similarities between NB3-3- derived neurons across segments.

We included the following text in the introduction to address this point (lines 67-76):

> NB3-3 is the only lineage that produces ELs (Bossing et al., 1996; Gunnar et al., 2016). All EL neurons require somatosensory stimuli to fire, making them sensory processing neurons (Heckscher et al., 2015). Generally, ELs are commissural and excitatory, like their counterparts in the vertebrate spinal cord, the commissural, excitatory, EVX-expressing (Eve homolog) V0v interneurons (Heckscher et al., 2015). The initial assumption was that all ELs were similar, but this was challenged because only late-born ELs express an 11F02 enhancer fragment (Heckscher et al., 2014). Independently activating the early-born and late-born ELs triggered escape and postural control behavioral responses, respectively (Wreden et al., 2017). Early-born ELs respond to vibrational stimuli, whereas late-born ELs respond to proprioceptive stimuli (Wreden et al., 2017).

(2) time is continuos, what defines early and late born cohorts are for example drawn in "the model that arises from these data" in fig 2G.

We describe how the field has approached the concept of temporal cohorts in the introduction. See the response to reviewer 1 for that text, or the main Introduction.

> More specifically, with regard to the model in 2G, we split the text into two separate paragraphs t Taken together therefor, these data suggest that the two major proliferation decisions— division type and division duration— are implemented combinatorially in the NB3-3 lineage to produce four different proliferation patterns, one in each CNS region. In the SEZ, we propose NB3-3s divide throughout neurogenesis, producing proliferative GMCs at each division and generating a lineage of ~24 neurons, 12 Notch ON/A and 12 Notch OFF/B (Figure 2H). In the thorax, NB3-3s divide only early in neurogenesis, producing proliferative GMCs at each division, thereby generating a lineage of ~12 neurons, 6 Notch ON/A and 6 Notch OFF/B (Figure 2H). In the abdomen, NB3-3s divide throughout embryogenesis, usually directly producing neurons. The lineage size is ~12 neurons; all except one are Notch OFF/B ELs (Figure 2H). In the terminus, we propose NB3-3s divide only early in neurogenesis (Figure 2H). The lineage size might be ~6 neurons, most of which are early-born Notch OFF/B ELs (Figure 2H).
> We pose this as a general model that we tested with additional experiments.

© 2026. Published by The Company of Biologists under the terms of the Creative Commons Attribution License (https://creativecommons.org/licenses/by/4.0/).

Taking this model and extrapolating from what we know about NB3-3 temporal cohorts in segment A1 and the thorax, our data raise the possibility that, depending on the region, NB3-3 neuroblasts exhibit distinct proliferation patterns to generate different sets of temporal cohorts in each region (Figure 2H). Specifically, the SEZ might be comprised of four temporal cohorts—early-born Notch OFF/B (ELs), early-born Notch ON/A, late-born Notch OFF/B (ELs), and late-born Notch ON/A (Figure 2H). The thorax would have two temporal cohorts—early-born Notch OFF/B (ELs) and early-born Notch ON/A (Figure 2H). We already know that A1, and likely the rest of the abdomen, has early-born Notch OFF/B and late-born Notch OFF/B temporal cohorts (Wang et al., 2022, Figure 2H). In the terminus, there would only be an early-born Notch OFF/B temporal cohort (Figure 2H). In this scenario, NB3-3 would the early-born Notch OFF/B temporal cohort in every segment and combinations of other types of temporal cohorts on a region-by-region basis.o help clarify our logic (lines 213-236)

Technical: the ts-MARCM technique and its analysis is still quite different to understand if one is not already familiar with it.

We apologize for not better describing ts-MARCM. We were trying to be brief to stay within word limits. But in this version, we have tried to explain it more thoroughly. Here is our updated main text (lines 131-194).

We used an inducible lineage-tracing approach, Twin-Spot Mosaic Analysis with a Repressible Cell Marker (ts-MARCM), to understand the dynamic production of EL during neurogenesis (Yu et al., 2009). In ts-MARCM, initially, every cell is heterozygous: one chromosome with an FRT site, UAS-GFP, and UAS-RFP-RNAi, and the other with an FRT site, UAS-RFP, and a UAS-GFP-RNAi. In heterozygous cells, neither GFP nor RFP can be produced (Figure 2A'). Heat shocks are applied to the animal, which deliver FLP recombinase to random cell sets (Figure 2A"). When a dividing cell recombines, daughter cells can become homozygous (Figure 2A"), with one daughter capable of producing GFP and the other RFP. All the descendant cells of each daughter cell will also be homozygous, generating the potential for "twin-spot clones" (Figure 2C). Herein, "clone" refers to a group of cells labeled by one recombination event. Because GFP and RFP are under UAS control, they must be expressed using a GAL4 line (Figure 2A"").

In this study, we used ts-MARCM because of its ability to capture the dynamic process of neurogenesis. However, these data can be messy. First, the timing of heat-shock- induced recombination sets the upper limit on the number of cells within a clone. But the lower limit can depend on other factors. There may be cell-type specificity because the same RNAi constructs can have different efficacies in GMCs versus neuroblasts (Yu et al., 2009), and certain cell types could respond differently to externally applied heat shock. Moreover, the GAL4 driver line used in each experiment can set the lower limit. Herein, we use *EL-GAL4*, a small enhancer fragment (~700 bp) that does not capture the full regulatory context of the *eve* locus. *EL-GAL4* drives highly specific, but somewhat variable, expression, sometimes labeling only a subset of cells (e.g., Manning et al., 2012). Thus, in some cases, clones detected with *EL-GAL4* may be smaller than expected.

In this study, we used ts-MARCM data produced by 11 different protocols (Figure 2B). In each protocol, embryos were collected for 2-3 hours and aged for 5 to 10 hours at 25- degrees (Figure 2B gray bars). Then they were heat-shocked for 20- 30 minutes (Figure 2B red bars). Embryos were grown to larval stages, then dissected and imaged. Part of our dataset (Table S1, experiments a-h) was produced for a prior study (Wang et al., 2022), which focused exclusively on segment A1. We re-analyzed them for this study and included all segments present in those images, which included from Th1 to Te2.
However, this dataset lacked information about the SEZ. So, we produced a second dataset (Table S1, experiments i-k), In this new dataset, we imaged all segments from the SEZ to the terminus (Table S1). However, for this second dataset, we biased heat shocks towards later in embryogenesis because our main question (see below) was whether the SEZ was abdomen-like (producing late-born ELs) or thoracic-like (not producing late-born ELs). Therefore, in the data presented below, we lack ts-MARCM experiments that included the SEZ for early neuroblast divisions. Combined, our data comprise 80 CNS and 234 clones. Every clone is listed in

© 2026. Published by The Company of Biologists under the terms of the Creative Commons Attribution License (https://creativecommons.org/licenses/by/4.0/).

Supplemental Table 1.
Despite this large dataset, there are notable gaps, which we discuss in more detail in the methods.

We used an internal reference to estimate when heat shocks were provided to the embryos to induce recombinase expression and initiate the lineage trace. This internal reference system operates on the developmental principle that, in short-germ- band insects like *Drosophila,* segments develop synchronously. So, it is reasonable to assume that neuroblasts are dividing at approximately the same time along the anterior- posterior axis, and there are no differences between left and right hemi-segments. We found that most CNSs had multiple clones (Figure 2D). For each CNS, we assessed the location and size of each clone. Because we know NB3-3 in the abdomen divides for the duration of embryogenesis (Gunnar et al. 2016), the number of EL labeled in the abdomen is directly related to the number of NB3-3 divisions yet to occur. If one or more clones were found within the abdomen, we used them to calculate the maximum abdominal clone size per CNS; this serves as our internal reference. The maximum abdominal clone size is a proxy for the earliest time at which an embryo could have been heat-shocked. We sorted each clone into bins based on its maximum abdominal clone size in its CNS. This binning generates the X-axis in Figure 2E-G and is labeled as "heat shock relative to inferred NB3-3 division number". Notably, because we used this method, we excluded the rare clones that were SEZ-, thorax-, or terminus-only from the analysis.

To determine the duration of division of NB3-3 in each region, we use this logic: A heat shock can generate a clone only when delivered to a dividing cell (Figure 2A, C), and never when delivered to a non-dividing cell. We counted the number of clones at each time point and region. In the abdomen, ELs were labeled at every inferred division (Figure 2E-F), as expected based on prior work (Gunnar et al. 2016). In the thorax and terminus, ELs were labeled only at early induction times, except for a single clone in the thorax produced in the inferred 11$^{th}$ division (see Methods) (Figure 2F). This was expected result for the thorax (Tsuji et al., 2008). In the SEZ, for which we have only late-induction data, ELs were labeled at all assayed time points (Figure 2F). This fits a general model in which NB3-3s in non-adjacent regions (abdomen and SEZ) divide for longer than in other non-adjacent regions (thorax and terminus) (Figure 2H).

2- Specific
This in turn raises an important major point: it is hard to understand the technical limitations and put the authors' interpretation in context. I think it would be essential to (1) briefly explain what the "heat shock time relative to inferred NB3-3 division number" is and how it's calculated in the main text. This is essential to understand a major part of the manuscript. (2) Clearly acknowledge the several instances in which the ts-MARCM system has limitations, as discussed in the exchange with reviewers. This can't just be limited to the methods sections as the reader needs to understand the limitations. The fact that the Methods section on ts-MARCM is now 5 pages to include all the experimental idiosyncrasies and limitations is very concerning. If so many clarifications are needed, I worry if the conclusions of the authors are really justified.

Thank you. Please see above for the text we added. It covers these suggestions. We use ts- MARCM to draw a model and use many experiments, not ts-MARCM alone to come to conclusions.

It is still unclear how the authors draw conclusions from SEZ if there are no early clones. I find it confusing to plot distributions of percentages of clones (Fig 2E,F) across timepoints and regions. It's impossible to observe SEZ early as they were not assayed but to anyone looking at the figure you might think they were assayed but not detected.

Thank you for this comment. It was helpful to know how we could further clarify this dataset. We added labels directly to the figure at locations where SEZ data are lacking and indicate which clones were not assayed (Fig 2F and 2G). And we added schematics in Fig 2B depicting heat shock protocols to illustrate our dataset and analysis.

© 2026. Published by The Company of Biologists under the terms of the Creative Commons Attribution License (https://creativecommons.org/licenses/by/4.0/).

Third decision letter

MS ID#: dev.205389R2

MS TITLE: How serially homologous neuroblasts produce different temporal cohorts along the Drosophila larval body axis

AUTHORS: Deeptha Vasudevan; Yi-wen Wang; Hannah Carr; Elise Paniel; Sean Corcoran; Chris Wreden; Elaine Kushkowski; Conor Lee-Smith; Ellie Heckscher

Dear Dr Heckscher,

I am happy to tell you that your manuscript has been accepted for publication in Development, pending our standard publication integrity checks.

© 2026. Published by The Company of Biologists under the terms of the Creative Commons Attribution License (https://creativecommons.org/licenses/by/4.0/).
